# Milk fat globule EGF and factor V/VIII domain containing (MFGE8) as a novel player in equine endometrial fibrosis

**DOI:** 10.1038/s41598-026-46595-7

**Published:** 2026-04-09

**Authors:** Elena zu Klampen, Gregor Neufeld, Claudia Klein

**Affiliations:** Institute of Farm Animal Genetics, Friedrich-Loeffler-Institut, Neustadt Am Rübenberge, Germany

**Keywords:** MFGE8, TGF-β1, CCN2, TAGLN, Equine endometrial fibrosis, Endometrosis, Cell biology, Diseases, Molecular biology

## Abstract

With increasing age, mares are frequently affected by endometrial fibrosis. Transforming growth factor beta 1 (TGF-β1) promotes fibrosis, while milk fat globule EGF and factor V/VIII domain containing (MFGE8) primarily exerts antifibrotic effects. We used in situ hybridization analysis (ISH) to investigate *MFGE8*, *TGFB1* and its downstream effectors cellular communication network factor 2 (*CCN2*) and transgelin (*TAGLN*) in equine endometrial fibrosis. Additionally, transcriptional effects of TGF-β1 and MFGE8 on equine endometrial fibroblasts in vitro were investigated by single cell sequencing. Fibrotic areas overexpressing *TGFB1* displayed increased *CCN2* and *TAGLN* expression. The vast majority of glandular areas overexpressing *MFGE8* were affected by fibrosis, confirming its relevance in equine endometrial fibrosis. Single cell sequencing following TGF-β1 exposure of fibroblasts confirmed its profibrotic effects. MFGE8 treatment effects were subtler, including decreased profibrotic leukemia inhibitory factor expression and an upregulation of interferon alpha and beta signaling in a subset of cells. In a fibroblast subpopulation, upregulation of phosphatase and tensin homolog signaling, indicated an antifibrotic effect of MFGE8. In conclusion, our study reaffirms the profibrotic effects of TGF-β1 and highlights MFGE8 as a novel player in equine endometrial fibrosis with our results suggesting an antifibrotic effect of MFGE8.

**Supplementary Information:**

The online version contains supplementary material available at 10.1038/s41598-026-46595-7.

## Introduction

As mares age, the likelihood of fibrotic degeneration, also referred to as endometrosis, increases^1^. The vast majority of mares older than 11 years display signs of chronic degenerative changes within the endometrium, with those changes being most often severe in mares older than 16 years. Properly functioning endometrial glands are crucial during early pregnancy through the production of histotroph, explaining the negative impact of endometrial fibrosis on fertility, as fibrotic changes primarily affect endometrial glands^2^. Fibrosis is the result of imbalanced mechanisms that orchestrate tissue repair and can affect almost any tissue in the body. Mechanisms of wound healing and the fundamental pathogenetic mechanisms of fibrosis are conserved to a certain extent across species and tissues^3^. The multifactorial nature and slow progression of fibrotic conditions make it difficult to elucidate their etiology and pathophysiology. Equine endometrial fibrosis is histomorphologically characterized by periglandular fibrosis, resulting in glands surrounded by multiple concentric layers of (myo-) fibroblasts and excess extracellular matrix (ECM), with multiple glands often forming so-called “nests”^4^. Histomorphological differentiation and glandular epithelial cell integrity may also be impaired^5^. Currently, there are no effective treatment options for equine endometrial fibrosis, and therapeutic strategies for other fibrotic diseases affecting humans are limited.

Myofibroblasts are a central player during the course of fibrotic degeneration. Myofibroblasts most commonly originate from normal fibroblasts through phenotypic transition^6^. Myofibroblasts are characterized by the expression of smooth muscle markers such as alpha-smooth muscle actin (α-SMA) and contribute to the progression of fibrosis through excess production of extracellular matrix components such as collagen and elastin^7,8^. In equine endometrial fibrosis, the presence of periglandular myofibroblasts has been reported^9^. Transforming growth factor beta 1 (TGF-β1) is a key cytokine in the development of fibrotic diseases, primarily by promoting myofibroblast differentiation and activation^10^ and endometrial tissue affected by fibrosis displays enhanced activity of TGF-β1^11^. Multiple other downstream effector molecules of TGF-β1, such as cellular communication network factor 2 (CCN2)^12^ and platelet-derived growth factors (PDGF)^13^ among others have been attributed a role in mediating the profibrotic effects of TGF-β1. Similarly, transgelin (*TAGLN*), an actin-binding protein and marker of smooth muscle cells, is induced by TGF-β1 and has been associated with fibrosis^14^.

Upon in situ hybridization we recently discovered the expression of milk fat globule EGF and factor V/VIII domain containing (*MFGE8*) surrounding endometrial glands affected by fibrotic changes (unpublished observation). The role of MFGE8 within the context of fibrotic changes in various organs has been studied quite extensively, with the majority of results revealing MFGE8 to exert antifibrotic effects^15–17^. MFGE8 consists of a C-terminal blood coagulation factor V and VIII-like discoidin C1 and C2 domain; the N-terminal side harbors a species-specific number of epidermal growth factor-like domains (EGF repeats) that contain an arginine-glycine-aspartic acid motif (RGD)^18^. The discoidin C1 and C2 domains can bind to phosphatidylserine on apoptotic cells^19^ or to collagens^15^, thereby mediating their uptake by macrophages that bind to the EGF repeat. In this manner, antifibrotic effects of MFGE8 have been described in post-infarct myocardial by promoting the uptake of apoptotic cells^20^ and pulmonary fibrosis via mediating the uptake of collagen^15^. Arteriosclerosis represents one notable exception to the antifibrotic nature of MFGE8, as MFGE8 promotes the proliferation of vascular smooth muscle cells and the osteogenic transdifferentiation during age-associated arterial wall remodeling^21^. The role of MFGE8 in the context of equine endometrial fibrosis has to the best of our knowledge not been investigated.

The aim of the present study was to investigate the roles of MFGE8 and TGF-β1 in the context of equine endometrial fibrosis. We hypothesized *MFGE8* to be expressed in fibrotic glands and exogenous MFGE8 to elicit a profibrotic transcriptional response in cultured endometrial fibroblasts. The expression of *MFGE8*, *TGFB1*, *CCN2,* and *TAGLN* was therefore examined through in situ hybridization in endometrial tissue. Single cell sequencing was used to determine the effects of MFGE8 and TGF-β1 on the transcriptome of equine endometrial fibroblasts in vitro. A single cell sequencing approach was chosen to account for potential heterogeneity among fibroblasts that might affect the transcriptional response to MFGE8 and/or TGF-β1.

## Results

### In situ hybridization analysis

#### Expression in non-fibrotic endometrium

**Fig. 1:**
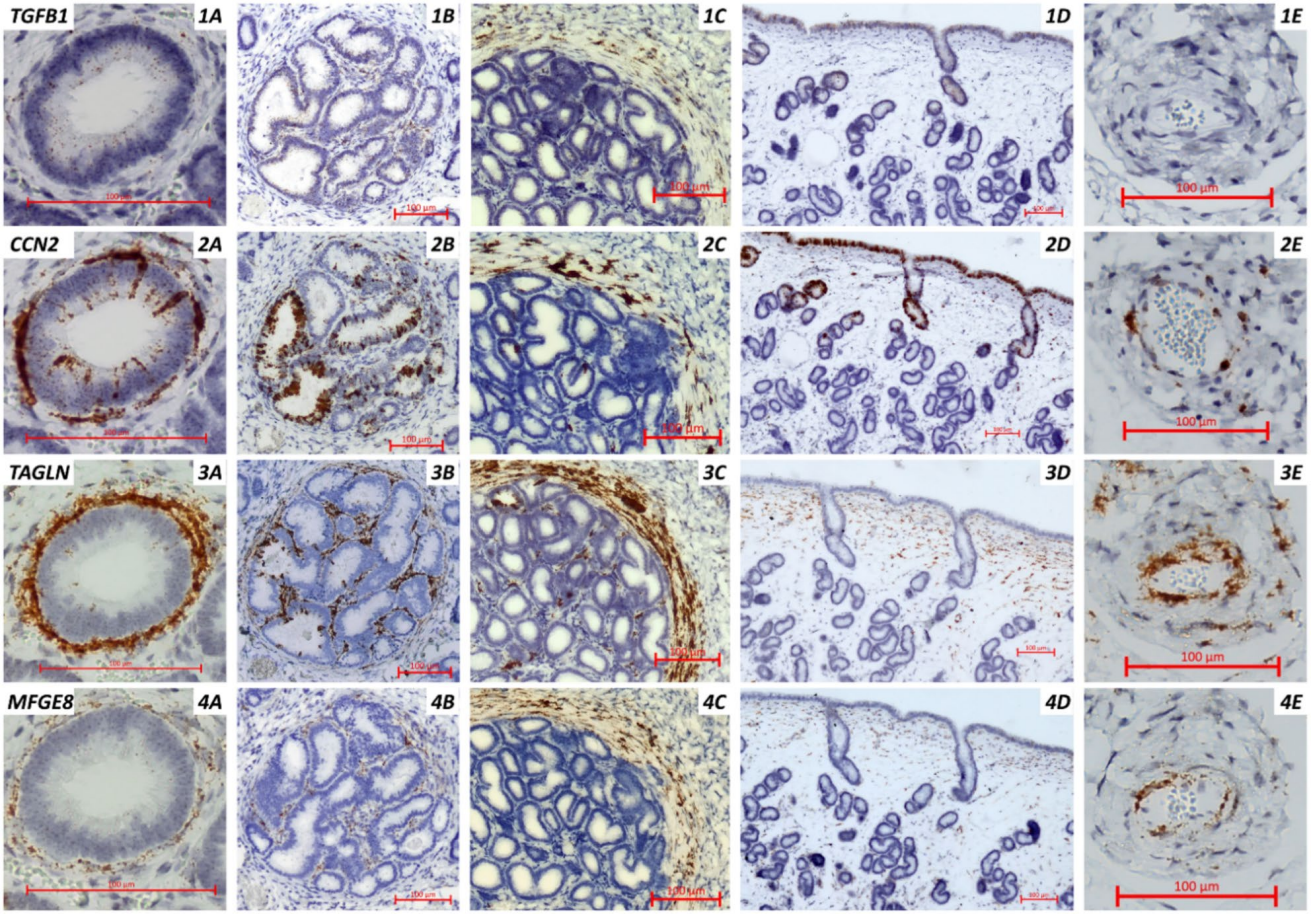
Exemplary localization of (1) *TGFB1*, (2) *CCN2*, (3) *TAGLN*, and (4) *MFGE8* expression in corresponding areas of equine endometrium (n = 8 mares) by in situ hybridization. Brown dots/areas indicate the expression of the respective transcript on sections counterstained with hematoxylin. (**A**) Single gland mildly affected by fibrosis with regular but sparse TGFB1 expression predominantly in the glandular epithelial cells, distinct expression of CCN2 in the glandular epithelial cells and the periglandular stromal cells, and overexpression of TAGLN and MFGE8 in the concentric periglandular stromal cells (**B**, **C**) Glandular nests severely affected by fibrosis with clear staining of all four genes, (**B**) predominantly in the central areas of the nest or (**C**) in the concentric layers of periglandular stromal cells. (**D**) Luminal epithelium and subluminal area with weak but regular epithelial expression of *TGFB1,* strong epithelial expression of *CCN2,* and subluminal stromal cell expression of *TAGLN* and *MFGE8*. (E) Vessel without *TGFB1* but with expression of *CCN2*, *TAGLN* and *MFGE8*. Bar 100 µm.

Distinct *CCN2* expression was visible in luminal epithelial cells and glandular epithelial cells located in close proximity to the luminal epithelium (except for one mare). This was not the case for the remaining glandular epithelial cells (Fig. 1 Supplementary Figure M1). Blood vessels were characterized by a strong expression of *TAGLN*, a clear expression of *CCN2,* and often by a marked expression of *MFGE8*. *TAGLN* and *MFGE8* expression was distinctly visible in stromal cells, with the strongest expression observed subluminally, gradually decreasing through the stratum compactum and becoming weaker in the deeper layers. Weak *TGFB1* expression was present in luminal and glandular epithelial cells (particularly subluminal) and more irregularly in stromal cells throughout the endometrium evident by regularly dispersed punctuate dot signals.

#### Expression in fibrotic endometrial glands

**Fig. 2:**
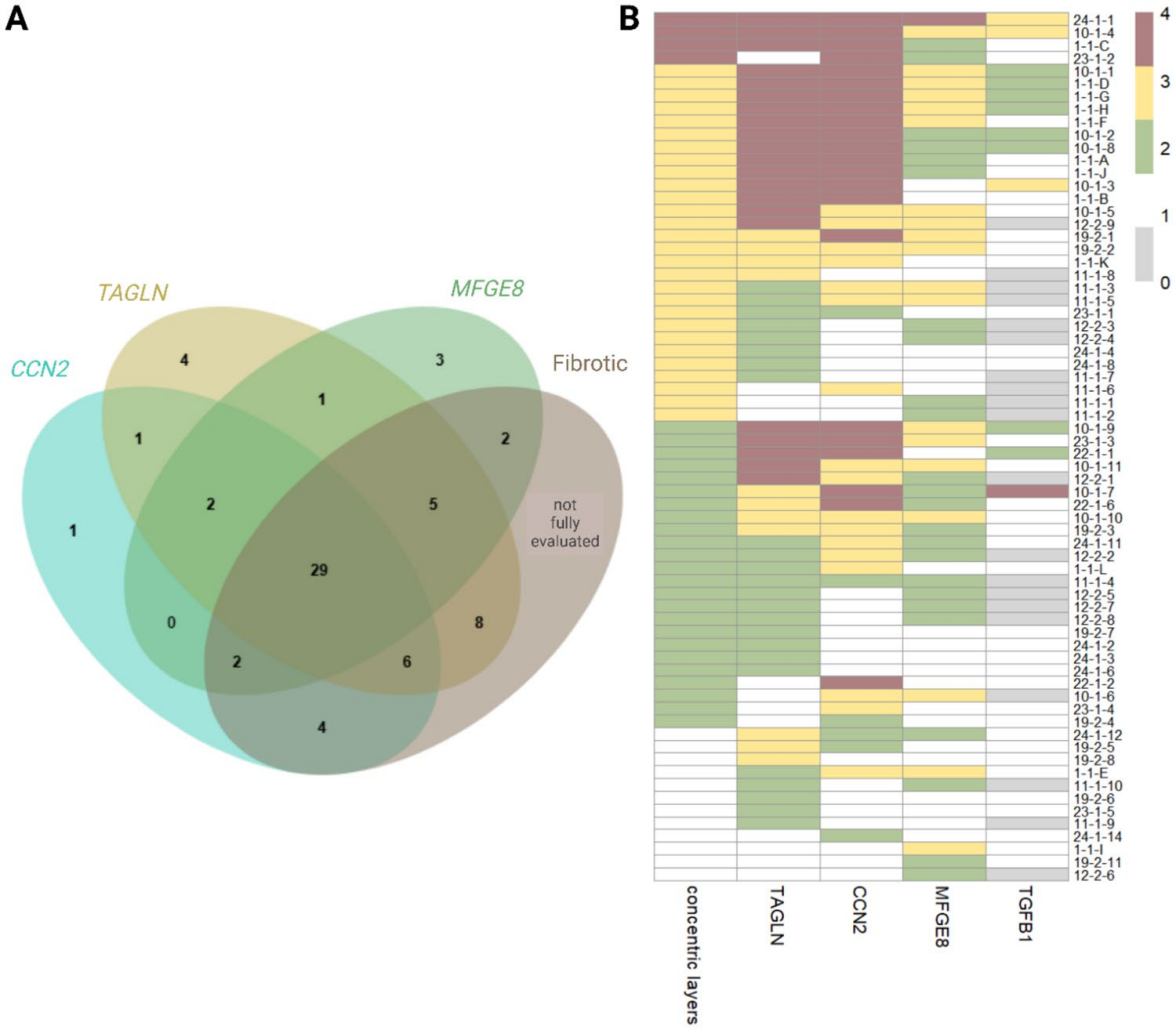
Co-expression of *TGFB1, CCN2*, *TAGLN*, and *MFGE8* in 68 endometrial glandular areas affected by fibrosis (n = 8 mares). (**A**) Venn diagram displaying results of the in situ hybridization analysis of the increased gene expression of *CCN2*, and/or *TAGLN*, and/or *MFGE8* in glandular areas affected by fibrosis. (**B**) Overview of the number of concentric fibroblasts layers and staining intensity of *TAGLN*, *CCN2*, *MFGE8* and *TGFB1*. The number of concentric layers is depicted as dark red ("4") for > 10 layers, yellow ("3") for 4 to 10 layers, green ("2") for 1 to 3 layers, and white ("1") for no layers. The staining intensity is denoted as red ("4") for intense, yellow ("3") for moderate, green ("2") for slightly more, and white ("1") for no difference compared to the basic gene expression. Grey (“0”) indicates areas which where not analyzed for this gene.

Sixty-eight glandular areas were examined, 56 of which displayed signs of fibrosis. Twenty-four glandular areas had 1 to 3 layers, 28 areas had 4 to 10 layers, and 4 areas had more than 10 layers of concentric fibroblasts. The remaining twelve glands presented no visible signs of fibrosis but were included in the analysis as they displayed slight or moderate staining for one or more of the genes investigated. Distinct fibrotic areas not displaying enhanced expression of one of the genes examined were infrequently present but were not included in the current analysis. Forty-four areas displayed increased MFGE8 staining, 38 of which were affected by fibrosis. The co-expression of the investigated genes and the corresponding histomorphologic evaluation of the area are depicted in Fig. 2 (for more details see Supplementary Figure M1 and Supplementary Table M2). *TGFB1* expression was determined for a smaller number of samples from 6 mares (n = 2 estrus, n = 4 diestrus; n = 3 category III, n = 2 category IIB, n = 1 category IIA; in total 48 glands), as the majority of glands affected by fibrosis did not display increased staining for *TGFB1* (mare #10 represented an exemption, with 7/10 fibrotic glands/nests expressing *TGFB1*). Instead, the expression and localization of the TGF-β1 downstream effector molecules *CCN2* and *TAGLN* were determined for all samples. In general, no clear cycle dependent pattern was discernable for fibrotic glands.

Increased expression of *CCN2* was present in 41/56 (73%), of *TAGLN* in 48/56 (86%), and of *MFGE8* in 38/56 (68%) of fibrotic glands. Expression of *CCN2* was evident in glandular epithelial cells and periglandular stromal cells, *TAGLN* expression was noted predominantly in periglandular stromal cells and occasionally in glandular epithelial cells, and *MFGE8* staining predominantly localized to periglandular stromal cells. Glandular areas displaying more than 10 layers of concentric fibroblasts (n = 4) all exhibited intense *CCN2* staining and slight to intense *MFGE8* staining, with 3 of 4 glands displayed intense staining for *TAGLN*. The 28 moderately fibrotic (4 to 10 layers) glandular areas exhibited 12/28 intense or 7/28 moderate staining for *CCN2*, 13/28 intense or 4/28 moderate *TAGLN* expression, and 11/28 moderate staining intensity for *MFGE8*.

Of the 48 glandular areas additionally investigated for *TGFB1*, 12 glands displayed increased staining. The evaluation of *TGFB1* expression by fibrotic areas was more difficult compared to the other targeted transcripts as glandular epithelial cells throughout the endometrium displayed low levels of *TGFB1* expression marked by regularly dispersed punctuate dot signals. *TGFB1* expression by glands was only categorized as increased if the punctuate staining dots were more pronounced than the expression in the remaining endometrium. With one exception, all glandular areas displaying an increased expression of *TGFB1* also exhibited intense staining for *CCN2* and *TAGLN*, and all—except for two areas—displayed weak to intense *MFGE8* staining.

### Single cell sequencing

**Fig. 3:**
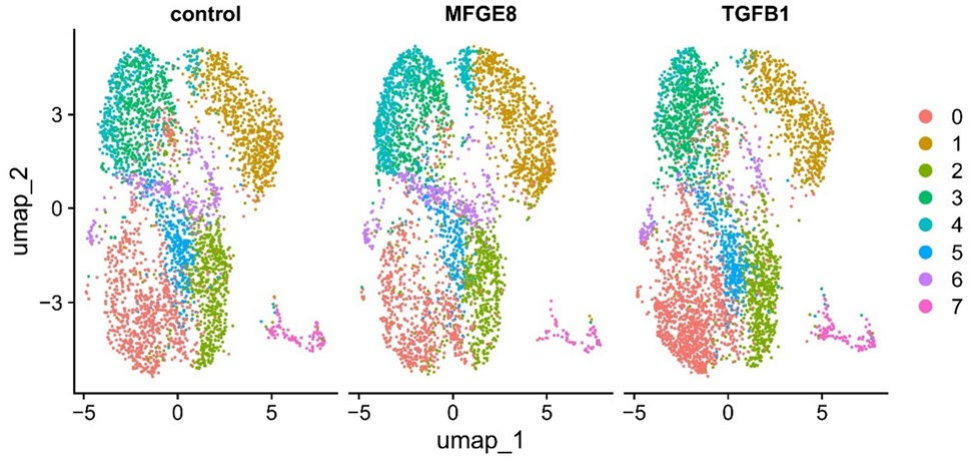
UMAP visualization of high dimensional single cell sequencing data for the control, MFGE8, and TGF-β1 (abbreviated as TGFB1 in this figure) treatment groups after integration. For each group, endometrial fibroblasts from different mares (n = 4) were pooled. In total, eight clusters (cluster 0–7) were present.

Initial alignment of single cell RNA-sequencing reads revealed an average of 152,130 reads per cell, with over 93% of reads being classified as within cells. The Barcode Rank Plot indicated no compromise in sample quality. Following the application of exclusion criteria, on average 955 cells per mare and treatment remained. A total of eight clusters were present and are displayed as color-coded UMAP plots in Fig. 3 and Supplementary Figure M2 (the number of cells per cluster is provided in Supplementary Table M3). All cells expressed *VIM* and *COL1A1*, while the expression of epithelial cell markers *EPCAM* and *CDH1* or the endothelial markers *PECAM* and *VWF* were negligible, confirming stromal cell identity.

#### Characterization of clusters within control, MFGE8, and TGF-β treated samples

**Fig. 4:**
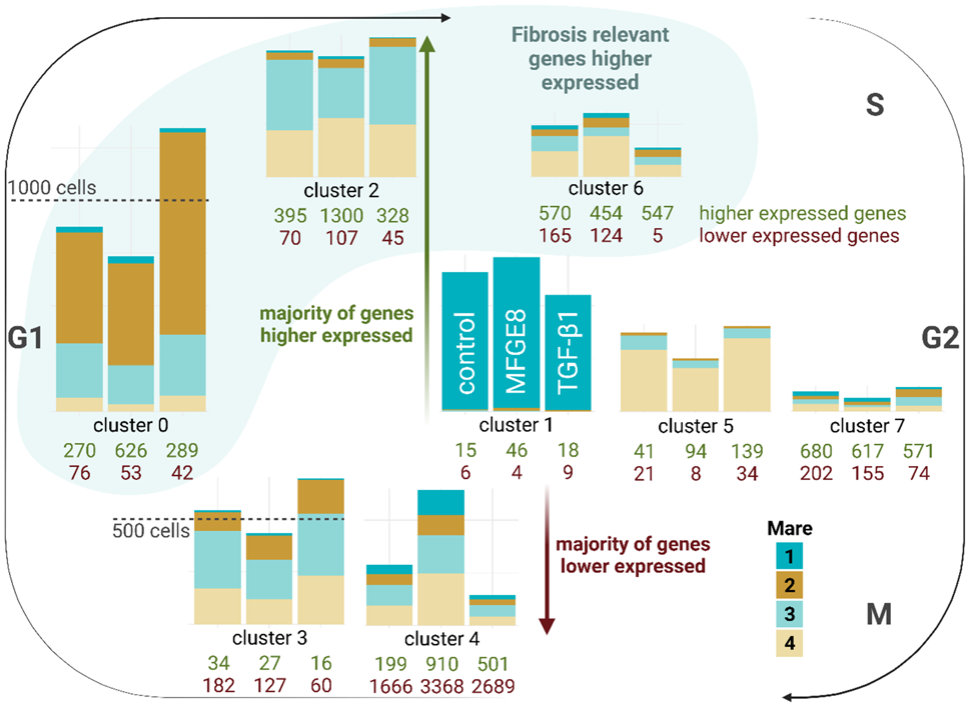
Heterogeneity of cultured fibroblasts isolated from equine endometrium (n = 4 mares), based on single cell sequencing analysis. The left column of each cluster represents the relative number of cells within the control group, the middle column represents the MFGE8 treatment group, and the right column represents the TGF-β1 treatment group. Upper green values below each column indicate the number of higher expressed genes, while lower red values indicate the number of lower expressed genes. Horizontal positioning of the clusters represents their concordance with a cell cycle-specific phase, based on Ingenuity Pathway Analysis.

**Fig. 5:**
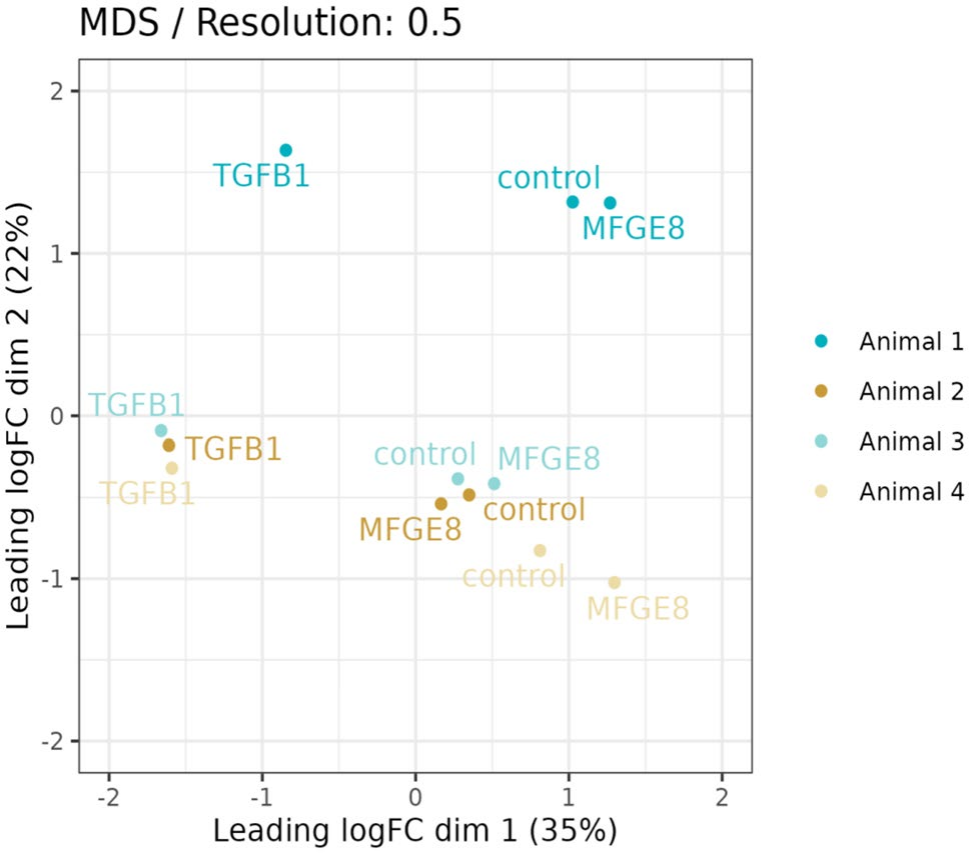
Effect of treatment and mare visualized in a multi-dimensional scaling plot. Equine endometrial fibroblast (n = 4 mares) were exposed to TGF-β1 (TGFB1) and MFGE8 for 24 h, followed by single cell sequencing.

Figure. 4 provides an overview of the number of transcripts differentially expressed in between clusters and a graphical overview of the main characteristics of these clusters (number of DEGs per cluster, presumptive cell cycle stage, and origin of cells based on mare identity). Supplementary Tables M4 M6 list all DEGs, and Supplementary Tables M7 to M9 provide details of the IPA pathway analysis. The MDS plot (Fig. 5) divides mare 1 and the remaining mares into two groups, accounting for 22% of the variability. This variability between mare 1 and the remaining mares is reflected in cluster 1, which is almost exclusively (98%) composed of cells from mare 1. Since cells were not labelled prior to pooling for single cell sequencing, is was not possible to assign individual samples to the individual mare they originated from following demultiplexing of sequencing results. For all treatment groups, cluster 7 contained cells in the DNA synthesis phase (S phase), gap 2 (G2) phase, or undergoing mitosis (as determined by IPA pathway analysis). Cells belonging to clusters 0 and 3 were characterized as being in the mitotic G1 phase and G1/S transition and cells belonging to clusters 1, 2, and 4 are most likely to contain cells in the G1 phase.

Within control samples, cells belonging to clusters 1 and 5 did not display any significant results upon pathway analysis. The most significant enriched pathway for cluster 2 was “SUMOylation of intracellular receptors.” Cluster 4 displayed the highest number of differentially expressed genes compared to the remaining clusters. The pathways “PTEN Signaling” and “PPARα/RXRα Activation” were significantly enriched. A large number of pathways was significantly underrepresented. Regarding fibrosis-relevant genes, clusters 0 and 6, and to a lesser extent cluster 2, were of interest. Cells belonging to cluster 6 were characterized by a considerable number of enriched pathways pertaining to fibrosis, such as “Hepatic Fibrosis/Hepatic Stellate Cell Activation,” “Collagen biosynthesis and modifying enzymes,” and “Wound Healing Signaling Pathway,” amongst others. Cluster 0 was positively associated with “Smooth Muscle Contraction.”

#### Differential gene expression analysis following MFGE8 and TGFB1 exposure

 Table 1 provides an overview of the number of transcripts differentially expressed in between treatments, Supplementary Tables M10 and M11 list all DEGs, and Supplementary Tables M12 and M13 provide details of the IPA pathway analysis. The MDS plot (Fig. 5) revealed the distance between control and MFGE8-treated samples within cell lines to be much smaller than the distance between control and TGF-β1-treated samples, reflecting the stronger effect of TGF-β1 versus MFGE8 on gene expression.

**Table 1:** Number of significantly higher and lower expressed genes per cluster after differential gene expression analysis following MFGE8 and TGF-β1 treatment. Each cluster from the treatment group was compared to the corresponding cluster from the control group. Cells in cluster 4 were highly responsive to MFGE8. Results from cultured equine endometrial fibroblasts (n = 4 mares).

		cluster							
		0	1	2	3	4	5	6	7
control vs MFGE8	higher	3	1	27	4	45	18	5	1
	lower	11	20	4	12	795	22	9	3
control vs TGF-β1	higher	505	37	491	407	333	455	243	296
	lower	506	49	619	455	628	484	303	258

Exposure to TGF-β1 significantly activated pathways related to collagen metabolism such as “Collagen biosynthesis and modifying enzymes” or “Assembly of collagen fibrils and other multimeric structures” in all clusters, although not all z-scores reached the threshold of 2. Fibronectin 1 (*FN1*) was significantly upregulated in all clusters except cluster 1 after TGF-β1 exposure. Likewise, the pathways “Complement Cascade” and “Interferon alpha/beta signaling” were significantly affected in all clusters, with all clusters displaying a z score less than −2 (except cluster 1 for “Interferon alpha/beta signaling”), indicating an inhibition of these pathways. Likewise, “Interferon gamma signaling” was significantly downregulated in clusters 2–5. Treatment with TGF-β1 furthermore led to a negative regulation of mitosis in cluster 7. Several growth factors were upregulated following exposure of cells to TGF-β1, such as *PDGFA* (cluster 4), *PDGFB* (clusters 0, 2, 3, and 5), *PDGFC* (all clusters except cluster 1), *TGFA* (clusters 2, 3, and 5), and *AREG* (all clusters, mean FC 16.06). *IGFBP3* was also upregulated with a mean FC of 43.37 in all clusters except cluster 6.

Following exposure to MFGE8, cluster 4 contained the highest number of differentially expressed transcripts compared to the remaining clusters (840 DEGs versus 22 ± 18 DEGs). Pathways such as “Wound Healing Signaling Pathway,” “Pulmonary Fibrosis Idiopathic Signaling Pathway,” “WNT/Ca + pathway,” and “RHO GTPase cycle” were significantly downregulated, while “PTEN Signaling” underwent a significant activation. Cluster 2 and 5 were characterized by a significant enrichment of “Interferon alpha/beta signaling” and cluster 1 by a significant enrichment of “Oxidative Phosphorylation.” Supplementary Figure M3 depicts detailed results of “PTEN signaling” for cluster 4 of both MFGE8 and TGF-β1 treated cells.

## Discussion

The present study investigated the expression of *MFGE8*, *TGFB1*, *CCN2,* and *TAGLN* in equine endometrium affected by fibrotic changes using in situ hybridization and determined the effects of MFGE8 and TGF-β1 on the transcriptional response of equine endometrial fibroblasts in vitro through single cell sequencing. Importantly, instead of using conventional tissue culture plastic (TCP), fibroblasts were cultured on collagen-coated hydrogels with a stiffness value of 25 kPa. TCP has an unphysiologically high stiffness, which promotes myofibroblast formation. In a recent study we demonstrated the effect of matrix stiffness on the gene expression of equine endometrial fibroblasts in vitro and their transcriptional response to TGF-β1^46^.

In situ hybridization revealed the vast majority of glandular areas overexpressing MFGE8 to be affected by fibrotic degeneration (86%), underlining its relevance in equine endometrial fibrosis. *TGFB1* was predominantly expressed in glandular epithelium and to a lesser extent in periglandular stromal cells, however, the enhanced expression in fibrosis-affected glands was inconsistent. Increased *TGFB1* expression was evident in 25% of examined glandular areas, all of which also highly expressed *TAGLN*, a smooth muscle marker, and *CCN2*, a well-known downstream effector molecule of TGF-β1. Vice versa, not all glands with an increased expression of *TAGLN* and/or *CCN2* displayed an elevated signal for *TGFB1*. The presence of fibrotic glands displaying only basic *TGFB1* expression in the presence of intense *CCN2* and *TAGLN* expression likely indicates that temporarily elevated levels of *TGFB1* during the active phases of fibrotic progression are sufficient to induce the more stable expression of its downstream molecules *CCN2* and TAGLN, which both revealed a high level of concordance. Distinct temporal expression patterns of TGF- β1 in the course of wound healing have previously been reported^22^. Our data indicate that *CCN2*, which is known to be regulated by TGF-β1^23^, is a suitable downstream effector molecule of TGF-β1 to be investigated in lieu of the temporarily elevated *TGFB1.* Likewise, our single cell sequencing results revealed an upregulation of *CCN2* following exposure of equine endometrial fibroblasts to TGF-β1 in four of eight clusters. *CCN2* expression is, like *TGFB1,* mainly evident in glandular epithelial cells, suggesting that the signals responsible for the progression of fibrosis may originate primarily from the epithelium and subsequently exert their influence on periglandular stromal cells. Interestingly, within fibrotic nests a high variation in epithelial *CCN2* expression of adjacent glands was observed, indicating a non-uniform activity in fibrosis progression. Increasing levels of CCN2 have been reported in fibrotic tissue of various organs^24,25^ and our data support a central role of CCN2 in equine endometrial fibrosis based on the observed expression patterns.

Transgelin (TAGLN), one of the earliest markers of smooth muscle differentiation^55,56^, was included in the analysis as a potential novel marker for myofibroblasts. *TAGLN*, like *CCN2*, is a TGFβ-inducible gene^26^, which was confirmed by our single cell sequencing data. Given that the vast majority of glands marked by intense expression of *TAGLN* also displayed intense staining for *CCN2* and vice versa, it appears that *TAGLN* expression is most prominent in fibrotic glands that are in a transcriptional active state of fibrotic progression. The observation that all fibrotic glands overexpressing *TGFB1* also highly expressed *TAGLN* and the fact that TAGLN is one of the earliest markers of smooth muscle differentiation^26^ and has been associated with other fibrotic diseases further supports this assumption^14,27^.

It is of interest to note that twelve glands without any noticeable signs of fibrosis, such as concentric layers of fibroblasts, excessive extracellular matrix deposition, or dilation, nevertheless displayed increased expression of one or more of the transcripts investigated. Presumably, these glands are in a very early stage of fibrotic transformation. One intention of examining the potential co-expression of *MFGE8* with known markers of fibrotic progression such as *CNN2* and a smooth muscle marker was to address the question of whether MFGE8 is a cause of fibrosis or a response to fibrotic degeneration in the context of equine endometrial fibrosis. In case of a profibrotic effect of MFGE8, similar to the actions of MFGE8 in atherosclerosis^28^, we would have expected to observe a considerable number of glands expressing *MFGE8* prior to the increased expression of *CNN2* and *TAGLN*. Such a staining pattern was observed only in three non-fibrotic (and two fibrotic) glands. On the other hand, such a staining pattern would also be present if *MFGE8* were to exert an antifibrotic action. It is therefore not possible to draw a conclusion on the role of MFGE8 in equine endometrial fibrosis solely based on the in situ expression pattern, which prompted us to investigate the transcriptional response of endometrial fibroblasts to MFGE8 in vitro.

Single cell sequencing revealed a distinct response of cells to MFGE8 and TGF-β1 and furthermore revealed a certain degree of heterogeneity of cultured fibroblasts. Since cells were not labelled prior to pooling for single cell sequencing, it was not possible to assign individual samples to the individual mare they originated from following demultiplexing of sequencing results. Differing Kenney Doig scores could be a possible explanation for cluster 1 being composed exclusively of cells from mare 1. Heterogeneity was furthermore in part attributable to the cell cycle, with cells undergoing S phase, G2 phase, and mitosis forming a distinct cluster. Given that the majority of clusters contain cells from all four mares, it is less likely that the Kenney and Doig score impacted the results of the differential gene expression analysis. Cells belonging to cluster 4 revealed the most distinct gene expression pattern, as reflected by the high number of transcripts differentially expressed compared to the remaining clusters. The unique character of cells belonging to cluster 4 was also reflected by a distinct response to MFGE8 exposure not observed in the remaining clusters. Whether this observed heterogeneity of cells is comparable to freshly isolated equine endometrial fibroblasts is unknown. Given that cells from all 4 mares contributed more or less equally to cluster 4 increases the likelihood that this heterogeneity is also present in vivo.

Exposure of equine endometrial fibroblasts to TGF-β1 resulted in the transcriptional alteration of various transcripts encoding for members of the collagen family, Likewise, the ECM protein FN1 was upregulated after TGF-β1 exposure, which contributes to the excessive matrix deposition in fibrosis in various organs and is a potential target for antifibrotic treatment^29^, confirming previous studies which demonstrated an up-regulation of collagen and *FN1* expression in equine endometrial fibroblast in response to TGF-β1 exposure^30^. Transcriptional alteration of collagen cross-linking lysyl oxidase (*LOX*), members of metalloproteinase gene families (*MMPs* and *ADAMTS*) or their inhibitors, such as tissue inhibitor of metalloproteinases 1 (*TIMP1*) and testican 3 (*SPOCK3*) further confirms the impact of TGF-β1 on the course of fibrosis by influencing ECM remodeling^31,32^.

Cluster 1, composed almost exclusively of cells belonging to mare 1, comprises a subpopulation of cells more resistant to the profibrotic effects of TGF-β1, as evidenced by the lower number of differentially expressed genes compared to the remaining clusters. This deviation is potentially caused by the downregulation of “IL-17A Signaling in Fibroblasts”. Interleukin 17 A has previously been suggested to promote fibrotic-related processes in the equine endometrium^33^, while the inhibition of IL-17A signaling has been associated with a beneficial effect in lung and liver fibrosis in humans^34^.

Profibrotic effects of TGF-β1 are known to be mediated by platelet-derived growth factors (PDGF)^35^, which is reflected by an increase in *PDGFA*, *PDGFB,* and *PDGFC* transcript abundance in several clusters in the current study. TGF-α (structurally different from TGF-β) was upregulated in three clusters and has been associated with pulmonary fibrosis, in part through its effects on ECM regulation^36^. Amphiregulin (*AREG*), an autocrine growth factor for fibroblasts, displayed increased transcript abundance in all clusters (mean FC 16.06). The profibrotic effects of TGF-β1-induced overexpression of AREG have previously been described to drive pulmonary^37^ and hepatic fibrosis^38^ in humans, likewise in equine endometrium AREG has been implemented with dysregulated remodeling in fibroblasts^39^. Thus, the profibrotic effects of TGF-β1 likely are based not only on the direct effects of TGF-β1 itself, but also on its induction of other profibrotic growth factors.

Insulin-like growth factor binding protein-3 (IGFBP3) was highly upregulated (mean FC 43.37) in all but one cluster following TGF-β1 exposure. Similarly, exposure of WI-38 normal human lung fibroblasts to TGF-β1 for 24 h revealed IGFBP3 to be a highly induced transcript^40^, a phenomenon also observed in cardiac fibroblasts^41^. Pilewski, et al.^42^ described higher levels of IGFBP3 in fibrotic lung tissue and proposed that IGFBP3 contributes to fibrosis through its ability to stimulate the production of extracellular matrix components such as fibronectin and collagen type I in normal primary adult lung fibroblasts.

Exposure of endometrial fibroblasts to TGF-β resulted in significant inhibition of the complement cascade in every cluster. This finding is somewhat surprising as the complement cascade has been implicated as a mediator of pulmonary fibrosis. Gu, et al.^43^ demonstrated that TGF-β1 reduces the complement inhibitory proteins “cluster of differentiation 46” and “55” (CD45, CD55) in lung epithelial cells, the absence of which activates the complement cascade, followed by enhanced TGF-β1 expression due to a downregulation of SMAD7, an antagonist of TGF-β1. However, the downregulation of, e.g., C1S and SERPING1 following TGF-β1 present in our data were, to a lesser extent, also present in fetal foreskin fibroblasts^43^. This might be reflective of the fact that even though general mechanisms of fibrosis pathogenesis are conserved, there are distinct differences between tissue and cell types.

Exposure to TGF-β1 led to a downregulation of type-I interferon signaling in all but one cluster. Primary human airway fibroblasts have a reduced ability to produce the type-1 interferons alpha (IFN-α) and beta (IFN-β)upon prolonged exposure to TGF-β1^44^. Similarly, a reduction in type-II interferon (interferon gamma, IFN-γ) signaling was present in four clusters following exposure to TGF-β1. The actions of TGF-β and interferon signaling on diverse cellular functions often have opposing effects, as evidenced by an antagonistic effect on collagen synthesis: TGF-β stimulates collagen synthesis, while IFN-γ exerts an inhibitory effect on collagen synthesis^45^. Similarly, in lung fibroblasts, IFN-γ treatment inhibited TGF-β1-induced effects such as increasing collagen production via the signal transducer STAT-1^46^. In a bleomycin-induced mouse model of pulmonary fibrosis, the increase of TGF-β1 in epithelial cells and the ECM was diminished by IFN-β treatment, reflecting a potential inhibitory effect of this type-I interferon on the progression of fibrotic changes^47^. The downregulation of type-I and type-II interferon signaling following TGF-β1 exposure reflects the profibrotic state induced by TGF-β1, as evidenced by the activation of pathways related to collagen metabolism, and likely allows for an increased production of collagen.

The presumptive antifibrotic nature of MFGE8 was mostly restricted to cells belonging to cluster 4, the cluster that displayed the highest number of differentially expressed genes following exposure to MFGE8. Pathways such as “Pulmonary Fibrosis Idiopathic Signaling Pathway” were significantly downregulated, pointing towards an antifibrotic effect of MFGE8. Pathway analysis further revealed “PTEN signaling” to be significantly enriched in cluster 4, reflected by a lower expression of *AKT3* and *MAPK1* amongst other transcripts. Phosphatase and tensin homolog (PTEN) has been attributed multiple roles in the context of fibrosis, such as promoting fibroblast apoptosis during collagen matrix contraction^48^, the inhibition of myofibroblast differentiation in vitro^49^, and the inhibition of type I and type III collagens and alpha smooth muscle actin expression in dermal fibroblasts^50^. Two studies have reported that the loss of PTEN in vivo promotes pulmonary fibrosis^49,51^ and in mice MFGE8 exerts antifibrotic effects in the course of liver fibrosis^52^. A closer look at the PTEN signaling pathway following the exposure to MFGE8 revealed the activation of PTEN signaling to result in a predicted inhibition of cell migration, cell growth, cell cycle progression, and actin cytoskeleton regulation. It is therefore likely that MFGE8 exerts an antifibrotic effect via PTEN signaling in the current study. IPA analysis revealed “PTEN signaling” to be also significantly enriched, but below the z-score threshold of 2, in cells belonging to cluster 4 following treatment with TGF-β1. A closer look at the PTEN signaling pathway however revealed the effects to be opposite from those of MFGE8, i.e. resulting in the predicted activation of cell migration, cell growth, cell cycle progression, and actin cytoskeleton regulation, while the prediction for apoptosis resulted in inhibition.

Cells belonging to cluster 4 were furthermore characterized by a negative regulation of the non-canonical WNT/Ca^2^⁺ pathway following exposure to MFGE8, both of which have been implicated in the pathogenesis of fibrotic conditions. While canonical WNT signaling is increased in patients suffering from idiopathic pulmonary fibrosis^53^, non-canonical WNT pathways and their complex interactions with the canonical pathway have been less studied in the context of fibrosis. The negative association with the WNT/Ca^2^⁺ pathway was, among other reasons, based on the downregulation of non-canonical *WNT5B*. WNT5B has been demonstrated to imitate a TGF-β1-induced fibroblast activation by increasing fibronectin, α-smooth muscle actin, and CCN2 expression in lung fibroblasts^54^. In synovial mesenchymal stem cells, WNT5B contributed to fibrosis by upregulation of collagen I expression^55^. The MFGE8-induced reduction of *WNT5B* supports an antifibrotic effect of MFGE8. MFGE8 exposure also had a negative impact on the RHO GTPase cycle in cluster 4 and. RHO GTPase signaling mediates fibroblast to myofibroblast transition^56^ and inhibition of RHO GTPase signaling has been discussed as a potential treatment option for fibrotic diseases^57^. These findings align with an antifibrotic effect of MFGE8.

In contrast to TGF-β1, exposure of fibroblasts to MFGE8 led to an upregulation of “Interferon alpha/beta signaling” in cluster 2 and 5, which matches previously published results for primary human intestinal myofibroblasts that responded to MFGE8 exposure with an increase in interferon alpha and beta signaling and a reduced production of ECM^58^. Since increased type-I interferon signaling demonstrated inhibitory effects on fibrosis^47^, this finding supports further an antifibrotic role of MFGE8.

Following MFGE8 exposure, leukemia inhibitory Factor (LIF) transcript abundance was reduced in five clusters. LIF, a member of the interleukin 6 family, is a pleiotropic cytokine that has been associated with fibrosis. In murine and human renal fibrotic lesions enhanced expression of LIF can be observed and LIF overexpression leads to renal fibrosis, whereas the knockdown of its receptor (LIFR) reduces the occurrence of renal fibrosis^59^. In fibrotic foci of idiopathic pulmonary fibrosis-affected tissue, co-expression of LIF and its corresponding receptor has been reported, and the profibrotic effects of TGF-β1 on pulmonary fibroblasts can be blocked in vitro through blocking LIFR^60^. The reduced expression of LIF following MFGE8 treatment might therefore be of antifibrotic nature. The reduced expression of LIF following MFGE8 treatment might therefore have an antifibrotic nature. A structurally truncated form of MFGE8, NP-011, has already been successfully tested to counteract the profibrotic effects of TGF-β1 in vitro and in vivo models of liver^17^ and pulmonary fibrosis^61^, and was recently tested in a phase 1 clinical trial^62^.

The expression of *TNFAIP3* (TNF alpha-induced protein 3 or A20), a zinc finger and ubiquitin-editing enzyme, was reduced in five clusters following MFGE8. Reduced levels of TNFAIP3 have been observed in skin and lung tissue affected by fibrotic changes, and fibroblasts exposed to TNFAIP3 in vitro display a reduced profibrotic response^63^. The lower expression of TNFAIP3 in the present study might therefore hint at a profibrotic effect of MFGE8.

The effects of MFGE8 were less pronounced compared to TGF-β1. Our data revealed opposing effects of MFGE8 and TGF-β1 on interferon beta signaling and their predicted effects in the context of PTEN signaling in a subpopulation of fibroblasts. Competing effects of both molecules have been previously reported, for example, MFGE8 binding to αvβ3 integrin receptors blocks the activation of latent TGF-β1^64^ and leads to a reduction in TGF-β1 receptor expression^52^. This raises the possibility that MFGE8 may be more effective in inhibiting other profibrotic effects, rather than having strong intrinsic antifibrotic effects. Moreover, effects beyond the transcriptional level require a different experimental setup. A further limitation was the limited number of animals used for single cell sequencing, the inclusion of samples from mares with differing Kenney and Doig categories, and failure to collect samples from mares with a synchronized cycle. A potential effect of endometrial category or individual variability cannot be fully excluded.

## Conclusion

In summary, our study confirms TGF-β1 as a driver of fibrosis in the equine endometrium. An increase in *TGFB1* expression was consistently associated with an increased expression of the profibrotic *CCN2* and the smooth muscle gene *TAGLN*. *TAGLN* displays potential as a promising myofibroblast marker in equine endometrial fibrosis. The profibrotic effects of TGF-β1 demonstrated by single cell sequencing included alterations in ECM remodeling, inhibition of type I and type II interferon signaling, and increased expression of growth factors such as PDGFs. Moreover, *IGFBP3* and the growth factor *AREG* were increased following TGF-β1 treatment in equine endometrial fibroblasts, the upregulation of which has recently been associated with equine endometrial fibrosis.

The vast majority of *MFGE8*-overexpressing glandular areas were affected by fibrotic degeneration, confirming the relevance of MFGE8 in equine endometrial fibrosis. The effects of MFGE8 treatment demonstrated by single cell sequencing were subtler compared to TGF-β1 and revealed a subpopulation of fibroblasts that responded with increased PTEN signaling, coupled with a reduction in WNT/Ca^2^⁺ pathway and RHO GTPase cycle signaling, overall indicating an antifibrotic effect of MFGE8. Additionally, MFGE8 was associated with an increase in interferon type I signaling, and downregulation of profibrotic *LIF*.

Future studies should consider the potential competing effects of both MFGE8 and TGF-β1 to further delineate the potential antifibrotic nature of MFGE8 in equine endometrial fibrosis.

## Materials and methods

### In situ hybridization

Surplus endometrial tissue samples obtained from cycling mares within the course of breeding soundness examinations was donated to research and utilized in the present study (Keros Embryo Transfer Center, Dr. Denis Necci). Experimental protocols were approved by the institutional animal welfare committee and were carried out in accordance with the German Animal Welfare Act. Tissues were immediately fixed in 10% neutral buffered formalin for 24 h and transferred to 70% ethanol prior to paraffin embedding. From the formalin-fixed, paraffin-embedded (FFPE) samples, sections of 5 µm were cut in series using a microtome (HM325 manual microtome, Thermo Scientific, MA, USA) and mounted on Superfrost plus slides (Superfrost®Plus, VWR International BVBA, Belgium). Biopsies from eight mares (n = 2 in estrus, n = 6 in diestrus) aged 15 to 20 years with varying degrees of fibrotic alterations were selected for further analysis by in situ hybridization. In situ hybridization was performed using RNAScope® technology (Advanced Cell Diagnostics, CA, USA). Reagents were used as recommended by the manufacturer. Custom-designed probes targeting *MFGE*8 (XM_023650232.2), *TGFB1* (XM_005596086.4), TAGLN (NM_001110134.1), and *CCN2* (XM_023651101.2) were implemented. Slides were baked at 60 °C for 1 h followed by deparaffinization. Target retrieval was carried out using Target Retrieval buffer for 15 min at ~ 98–100 °C followed by applying hydrogen peroxide solution for 10 min at room temperature. Protease pretreatment was carried out for 20 min at 40 °C. A hydrophobic barrier was drawn around the tissue, followed by target probe hybridization at 40 °C for 2 h. Signal amplification steps were carried out using AMP1–AMP6 at 40 °C as described by the manufacturer followed by HRP based diaminobenzidine (DAB) color development for 10 min. Slides were briefly immersed hematoxylin, dehydrated and a coverslip was mounted using a xylene-based, non-aqueous medium. Stained sections were evaluated by light microscopy (Axioscope 5, Zeiss, Germany, Plan-Apochromat 20x/0.80 Ph2 M27). Areas with increased expression of at least one gene were analyzed using a semi-quantitative four-level grading scale from “none or basic” (1) to “slight” (2), “moderate” (3), and “intense” (4). “None or basic” implies there was no detectable signal at all or staining intensity was similar to the remaining non-fibrotic endometrial tissue. “Slight” indicates slightly more staining in the corresponding glandular areas compared to the remaining areas. “Intense” was used to describe a level of staining so pronounced that individual stained spots were indistinguishable from each other, while “moderate” staining was judged to be in between “mild” and “intense.” Concentric layers of fibroblasts were recorded in accordance with Schoon, Schoon and Klug^65^ and classified into “no” (1), “mild” (2), “moderate” (3), and “severe” (4) fibrosis based on the scheme by Kenney and Doig^37,66^. A total of 68 glands were evaluated.

### Single cell sequencing

#### Isolation of endometrial fibroblasts

Endometrial fibroblasts were kindly provided by the Institute of Animal Reproduction and Food Research, Polish Academy of Sciences, Olsztyn (Dr. Anna Szóstek-Mioduchowska) and were obtained from mares with healthy endometria or solely mild fibrotic alterations in the absence of inflammatory cells (n = 4; mid-luteal phase, Kenney and Doig category I n = 3 and category IIA n = 1) using abattoir material (No experimental procedure was performed on live animals). Fibroblast isolation and classification of estrous cycle stage were performed as described by Szóstek-Mioduchowska, et al.^67^. Once reaching 90% confluence, fibroblasts were cryopreserved and shipped to our laboratory on dry ice. To obtain sufficient quantities of cells for subsequent experiments, cells were then passaged onto collagen (collagen type I, rat tail, Sigma-Aldrich) coated TCP flasks using our standard cell culture medium consisting of Dulbecco’s modified Eagle’s medium high glucose (DMEM-HXA, Capricorn Scientific, Germany) supplemented with 2 mM L-glutamine (Capricorn Scientific), 0.1 mM 2-mercaptoethanol (Sigma-Aldrich), 1 × non-essential amino acids (Capricorn Scientific), 0.1 mM sodium pyruvate (Sigma-Aldrich), penicillin 200 units/ml, streptomycin 0.2 mg/ml (Capricorn Scientific) supplemented with 10% FBS (sterile-filtered, Capricorn Scientific) in a humidified atmosphere at 38 °C with 5% CO₂ and passaged at 80% confluency prior to the start of the single cell sequencing experiment.

#### Single cell sequencing

Equine endometrial fibroblasts (passage 3) were seeded onto collagen-coated hydrogels 12-well plates (SW12-COL coated with collagen type I, rat tail, Softwell™ from Matrigen® LLC, CA, USA) at a seeding density of 100,000 cells per well and incubated for 24 h in a humidified atmosphere at 38 °C and 5% CO₂ using our standard cell culture medium described above supplemented with 10% FBS. To mitigate the potential effects of FBS, the medium was subsequently replaced with a medium containing 4% FBS for 24 to 48 h. From 48 to 72 h (treatment phase), cells were incubated using cell culture medium containing 0.1% BSA (fraction V, Thermo Fisher Scientific, MA, USA) instead of FBS and received either (1) no further supplementation (control), (2) were supplemented with 10 ng/ml human recombinant TGF-β1 (PeproTech, NJ, USA), or (3) were supplemented with 10 ng/ml mouse recombinant MFGE8 (R&DSystems, MN, USA). Following 24 h of incubation, cells were detached by incubation with 0.05% trypsin–EDTA (Capricorn Scientific) and mechanical scraping of the hydrogel. Cell viability was confirmed using trypan blue live-dead staining (Supplementary Table M1) and cells were counted utilizing a Neubauer chamber. For each treatment group, cells from the four different mares (50,000 cells per mare) were pooled for further processing. Suspensions were subjected to three centrifugation steps (300 RCF, 4 min) followed by resuspension in 0.04% BSA (Ultrapure™, Thermo Fisher Scientific) in PBS. A final filtration through 40 µm and 20 µm cell strainers (PluriSelect, Germany) was performed. Viability and cell number were confirmed by trypan blue staining. Single-cell solutions containing 700 to 1,200 cells per microliter were processed for single cell sequencing using the Chromium Next GEM Single Cell 3’Reagent Kits v3.1, Dual Index (10 × Genomics, CA, USA) following the manufacturer’s instructions. Resulting libraries were subjected to paired-end (100 bp) sequencing as multiplexed pools on a NovaSeq 6000 using an S4 Reagent Kit v1.5 4XP (290 pM, 200 cycles; Illumina, CA, USA).

#### Processing of single cell sequencing reads

Data integrity of FASTQ files was confirmed by md5sum testing and data quality was checked using FastQC^68^. The reference genome EquCab3.0.109 and the associated general transfer format file were downloaded from Ensemble (www.ensembl.org). Genome indexing was performed using the Cell Ranger mkref tool (v.7.1.0, 10 × Genomics), and the Cell Ranger count tool (v.7.1.0) was used for alignment to the reference genome. Using Souporcell^69^, pooled data sets for each treatment group were separated into the four individual animals, and doublets were excluded from further analysis. Cells with at least 20,000 unique molecular identifiers (UMIs), at least 3,500 genes, and containing less than 4% mitochondrial genes were kept for further analyses. Data sets were then merged into a single Seurat object and normalized using Seurat^70^. Following the integration of data (Seurat v.5), visualization was performed through the generation of uniform manifold approximation and projection (UMAP, resolution of 0.5) and multi-dimensional scaling (MDS) plots. Differential gene expression analysis was conducted in edgeR 4.2.2. Differentially expressed genes (DEGs) were determined (1) in between clusters for control and each of the treated samples with the aim to characterize heterogeneity amongst fibroblasts and (2) in between treatments (control vs. MFGE8 and control vs. TGF-β1). Transcripts displaying a false discovery rate (FDR) of less than 0.05 and a fold change of greater than +/- 2 were considered differentially expressed. Ingenuity pathway analysis (IPA; Qiagen, Hildesheim, Germany) was used to predict downstream effects. Pathways exhibiting a z-score above 2 or below −2 and a significant -log(p-value) greater than 1.3 (corresponding to < 0.05) were considered biologically relevant.

## Supplementary Information

Supplementary Information 1.

Supplementary Information 2.

Supplementary Information 3.

Supplementary Information 4.

Supplementary Information 5.

Supplementary Information 6.

Supplementary Information 7.

Supplementary Information 8.

Supplementary Information 9.

Supplementary Information 10.

Supplementary Information 11.

## Data Availability

The sequencing data generated in this study have been deposited to Gene Expression Omnibus (GSE302877). To review GEO accession GSE302877: Go to https://www.ncbi.nlm.nih.gov/geo/query/acc.cgi?acc=GSE302877 Enter token gtydgagarhajhip.
