# Supplementary material for: Milk fat globule EGF and factor V/VIII domain containing (MFGE8) as a novel player in equine endometrial fibrosis
Source: Sci Rep. 2026 Apr 9;16:12045. doi: 10.1038/s41598-026-46595-7 (PMC13069092; doi:10.1038/s41598-026-46595-7)
Supplement: Supplementary file 11 — Supplementary Information 11. [file 41598_2026_46595_MOESM11_ESM.pdf]

Supplementary Table M1 Quality management (A) before and (B) after single cell sequencing of pooled equine endometrial fibroblasts (n = 4 mares).

(A) Sample preparation for scRNA-seq	*control*	*TGF-β1*	*MFGE8*
Cell viability (trypan blue live-dead staining) prior to loading into the ChromiumTM Controller	98.78%	95.60%	96.48%
Viable cell concentration prior to loading into the Chromium™ Controller *(cells/µL)*	1200	900	1000
Total volume of cell suspension loaded into the ChromiumTM Controller	9.6	12.8	11.6
Total number of viable cells loaded into the ChromiumTM Controller *(µL)**	11520	11520	11600
Targeted cell number	7000	7000	7000

**Cell count and viability were determined with trypan blue staining in a Neubauer chamber.*

(B) *Sequencing results*	*control*	*TGF-β1*	*MFGE8*	*mean*
Cells				
Estimated Number of Cells	4,384	5,124	4,413	4,640
Fraction Reads in Cells	95.1%	93.8%	93.8%	94.2%
Mean Reads per Cell	185,404	122,825	148,160	152,130
Mean UMI Counts per Cell	48,210	41,135	38,298	42,548
Median Genes per Cell	6,405	6,095	5,835	6,112
Total Genes Detected	18,252	18,270	18,106	18,209
Sequencing	***control***	***TGF-β1***	***MFGE8***	***mean***
Number of Reads	812,813,140	629,357,026	653,829,903	698,666,690
Number of Short Reads Skipped	0	0	0	0
Valid Barcodes	98.1%	97.8%	98.0%	98.0%
Valid UMIs	99.7%	99.7%	99.7%	99.7%
"Sequencing Saturation"	66.0%	53.3%	64.2%	61.2%
Q30 Bases in Barcode	95.8%	96.2%	96.1%	96.0%
Q30 Bases in RNA Read	93.2%	93.9%	93.9%	93.7%
Q30 Bases in UMI	95.2%	95.6%	95.5%	95.4%

Supplementary Figure M2 In situ hybridization results

Mare	Age	Cycle Phase	Category	Area	Concentric Layers	*TAGLN*	*CCN2*	*MFGE8*	*TGFB1*
1	15 - 18	estrus	III	1-1-D	3 (4 to 10)	4 (intense)	4 (intense)	3 (moderate)	2 (slight)
1	15 - 18	estrus	III	1-1-G	3 (4 to 10)	4 (intense)	4 (intense)	3 (moderate)	2 (slight)
1	15 - 18	estrus	III	1-1-H	3 (4 to 10)	4 (intense)	4 (intense)	3 (moderate)	2 (slight)
1	15 - 18	estrus	III	1-1-C	4 (> 10)	4 (intense)	4 (intense)	2 (slight)	1 (none or basic)
1	15 - 18	estrus	III	1-1-A	3 (4 to 10)	4 (intense)	4 (intense)	2 (slight)	1 (none or basic)
1	15 - 18	estrus	III	1-1-B	3 (4 to 10)	4 (intense)	4 (intense)	1 (none or basic)	1 (none or basic)
1	15 - 18	estrus	III	1-1-F	3 (4 to 10)	4 (intense)	4 (intense)	3 (moderate)	1 (none or basic)
1	15 - 18	estrus	III	1-1-J	3 (4 to 10)	4 (intense)	4 (intense)	2 (slight)	1 (none or basic)
1	15 - 18	estrus	III	1-1-K	3 (4 to 10)	3 (moderate)	3 (moderate)	1 (none or basic)	1 (none or basic)
1	15 - 18	estrus	III	1-1-L	2 (1 to 3)	2 (slight)	3 (moderate)	1 (none or basic)	1 (none or basic)
1	15 - 18	estrus	III	1-1-E	1 (none)	2 (slight)	3 (moderate)	3 (moderate)	1 (none or basic)
1	15 - 18	estrus	III	1-1-I	1 (none)	1 (none or basic)	1 (none or basic)	3 (moderate)	1 (none or basic)
10	20	estrus	III	10-1-4	4 (> 10)	4 (intense)	4 (intense)	3 (moderate)	3 (moderate)
10	20	estrus	III	10-1-3	3 (4 to 10)	4 (intense)	4 (intense)	1 (none or basic)	3 (moderate)
10	20	estrus	III	10-1-7	2 (1 to 3)	3 (moderate)	4 (intense)	2 (slight)	4 (intense)
10	20	estrus	III	10-1-1	3 (4 to 10)	4 (intense)	4 (intense)	3 (moderate)	2 (slight)
10	20	estrus	III	10-1-2	3 (4 to 10)	4 (intense)	4 (intense)	2 (slight)	2 (slight)
10	20	estrus	III	10-1-8	3 (4 to 10)	4 (intense)	4 (intense)	2 (slight)	2 (slight)
10	20	estrus	III	10-1-9	2 (1 to 3)	4 (intense)	4 (intense)	3 (moderate)	2 (slight)
10	20	estrus	III	10-1-6	2 (1 to 3)	1 (none or basic)	3 (moderate)	3 (moderate)	0 (not present)

Mare	Age	Cycle Phase	Category	Area	Concentric Layers	*TAGLN*	*CCN2*	*MFGE8*	*TGFB1*
10	20	estrus	III	10-1-5	3 (4 to 10)	4 (intense)	3 (moderate)	3 (moderate)	1 (none or basic)
10	20	estrus	III	10-1-10	2 (1 to 3)	3 (moderate)	3 (moderate)	3 (moderate)	1 (none or basic)
10	20	estrus	III	10-1-11	2 (1 to 3)	4 (intense)	3 (moderate)	3 (moderate)	1 (none or basic)
11	19	diestrus	IIB	11-1-1	3 (4 to 10)	1 (none or basic)	1 (none or basic)	2 (slight)	0 (not analysed)
11	19	diestrus	IIB	11-1-2	3 (4 to 10)	1 (none or basic)	1 (none or basic)	2 (slight)	0 (not analysed)
11	19	diestrus	IIB	11-1-3	3 (4 to 10)	2 (slight)	3 (moderate)	3 (moderate)	0 (not analysed)
11	19	diestrus	IIB	11-1-5	3 (4 to 10)	2 (slight)	3 (moderate)	3 (moderate)	0 (not analysed)
11	19	diestrus	IIB	11-1-6	3 (4 to 10)	1 (none or basic)	3 (moderate)	1 (none or basic)	0 (not analysed)
11	19	diestrus	IIB	11-1-7	3 (4 to 10)	2 (slight)	1 (none or basic)	1 (none or basic)	0 (not analysed)
11	19	diestrus	IIB	11-1-8	3 (4 to 10)	3 (moderate)	1 (none or basic)	1 (none or basic)	0 (not analysed)
11	19	diestrus	IIB	11-1-4	2 (1 to 3)	2 (slight)	2 (slight)	2 (slight)	0 (not analysed)
11	19	diestrus	IIB	11-1-10	1 (none)	2 (slight)	1 (none or basic)	2 (slight)	0 (not analysed)
11	19	diestrus	IIB	11-1-9	1 (none)	2 (slight)	1 (none or basic)	1 (none or basic)	0 (not analysed)
12	17	diestrus	IIB	12-2-3	3 (4 to 10)	2 (slight)	1 (none or basic)	2 (slight)	0 (not analysed)
12	17	diestrus	IIB	12-2-4	3 (4 to 10)	2 (slight)	1 (none or basic)	2 (slight)	0 (not analysed)
12	17	diestrus	IIB	12-2-9	3 (4 to 10)	4 (intense)	3 (moderate)	3 (moderate)	0 (not analysed)
12	17	diestrus	IIB	12-2-1	2 (1 to 3)	4 (intense)	3 (moderate)	2 (slight)	0 (not analysed)
12	17	diestrus	IIB	12-2-2	2 (1 to 3)	2 (slight)	3 (moderate)	2 (slight)	0 (not analysed)

Mare	Age	Cycle Phase	Category	Area	Concentric Layers	*TAGLN*	*CCN2*	*MFGE8*	*TGFB1*
12	17	diestrus	IIB	12-2-5	2 (1 to 3)	2 (slight)	1 (none or basic)	2 (slight)	0 (not analysed)
12	17	diestrus	IIB	12-2-7	2 (1 to 3)	2 (slight)	1 (none or basic)	2 (slight)	0 (not analysed)
12	17	diestrus	IIB	12-2-8	2 (1 to 3)	2 (slight)	1 (none or basic)	2 (slight)	0 (not analysed)
12	17	diestrus	IIB	12-2-6	1 (none)	1 (none or basic)	1 (none or basic)	2 (slight)	0 (not analysed)
19	16	diestrus	IIB	19-2-1	3 (4 to 10)	3 (moderate)	4 (intense)	3 (moderate)	1 (none or basic)
19	16	diestrus	IIB	19-2-2	3 (4 to 10)	3 (moderate)	3 (moderate)	3 (moderate)	1 (none or basic)
19	16	diestrus	IIB	19-2-3	2 (1 to 3)	3 (moderate)	3 (moderate)	2 (slight)	1 (none or basic)
19	16	diestrus	IIB	19-2-4	2 (1 to 3)	1 (none or basic)	2 (slight)	1 (none or basic)	1 (none or basic)
19	16	diestrus	IIB	19-2-7	2 (1 to 3)	2 (slight)	1 (none or basic)	1 (none or basic)	1 (none or basic)
19	16	diestrus	IIB	19-2-11	1 (none)	1 (none or basic)	1 (none or basic)	2 (slight)	1 (none or basic)
19	16	diestrus	IIB	19-2-5	1 (none)	3 (moderate)	2 (slight)	1 (none or basic)	1 (none or basic)
19	16	diestrus	IIB	19-2-6	1 (none)	2 (slight)	1 (none or basic)	1 (none or basic)	1 (none or basic)
19	16	diestrus	IIB	19-2-8	1 (none)	3 (moderate)	1 (none or basic)	1 (none or basic)	1 (none or basic)
22	16	diestrus	IIA	22-1-1	2 (1 to 3)	4 (intense)	4 (intense)	1 (none or basic)	2 (slight)
22	16	diestrus	IIA	22-1-2	2 (1 to 3)	1 (none or basic)	4 (intense)	1 (none or basic)	1 (none or basic)

Mare	Age	Cycle Phase	Category	Area	Concentric Layers	*TAGLN*	*CCN2*	*MFGE8*	*TGFB1*
22	16	diestrus	IIA	22-1-6	2 (1 to 3)	3 (moderate)	4 (intense)	2 (slight)	1 (none or basic)
23	16	diestrus	IIB	23-1-2	4 (> 10)	1 (none or basic)	4 (intense)	2 (slight)	1 (none or basic)
23	16	diestrus	IIB	23-1-1	3 (4 to 10)	2 (slight)	2 (slight)	1 (none or basic)	1 (none or basic)
23	16	diestrus	IIB	23-1-3	2 (1 to 3)	4 (intense)	4 (intense)	3 (moderate)	1 (none or basic)
23	16	diestrus	IIB	23-1-4	2 (1 to 3)	1 (none or basic)	3 (moderate)	1 (none or basic)	1 (none or basic)
23	16	diestrus	IIB	23-1-5	1 (none)	2 (slight)	1 (none or basic)	1 (none or basic)	1 (none or basic)
24	15	diestrus	III	24-1-1	4 (> 10)	4 (intense)	4 (intense)	4 (intense)	3 (moderate)
24	15	diestrus	III	24-1-4	3 (4 to 10)	2 (slight)	1 (none or basic)	1 (none or basic)	1 (none or basic)
24	15	diestrus	III	24-1-8	3 (4 to 10)	2 (slight)	1 (none or basic)	1 (none or basic)	1 (none or basic)
24	15	diestrus	III	24-1-2	2 (1 to 3)	2 (slight)	1 (none or basic)	1 (none or basic)	1 (none or basic)
24	15	diestrus	III	24-1-3	2 (1 to 3)	2 (slight)	1 (none or basic)	1 (none or basic)	1 (none or basic)
24	15	diestrus	III	24-1-6	2 (1 to 3)	2 (slight)	1 (none or basic)	1 (none or basic)	1 (none or basic)
24	15	diestrus	III	24-1-11	2 (1 to 3)	2 (slight)	3 (moderate)	2 (slight)	1 (none or basic)
24	15	diestrus	III	24-1-12	1 (none)	3 (moderate)	2 (slight)	2 (slight)	1 (none or basic)
24	15	diestrus	III	24-1-14	1 (none)	1 (none or basic)	2 (slight)	1 (none or basic)	1 (none or basic)

Supplementary Table M3 Number of endometrial fibroblasts per cluster (n = 4 mares).

Treatment	Mare	Cluster							
		**0**	**1**	**2**	**3**	**4**	**5**	**6**	**7**
**Control**	**1**	26	650	8	8	44	2	20	21
	**2**	528	4	35	90	51	14	33	16
	**3**	254	6	334	272	97	67	71	22
	**4**	67	0	223	171	95	291	121	35
**MFGE8**	**1**	33	712	14	12	118	0	22	19
	**2**	483	14	42	114	97	11	45	15
	**3**	185	3	237	187	182	33	43	9
	**4**	34	1	280	120	246	207	192	21
**TGFB1**	**1**	20	544	3	7	21	0	10	10
	**2**	959	7	42	162	27	12	35	35
	**3**	289	1	369	292	53	45	37	43
	**4**	75	0	249	232	43	347	56	26

Supplementary Figure M1 In Situ Hybridization Images

Mare 10-1

	Concentric layers	*TAGLN*	*CCN2*	*MFGE8*	*TGFB1*
10-1-1	4 to 10	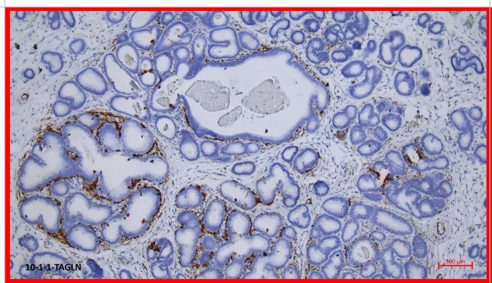 *TAGLN*, intense	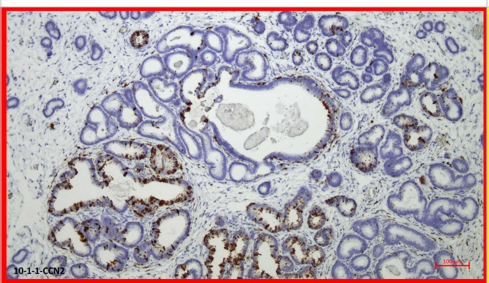 *CCN2*, intense	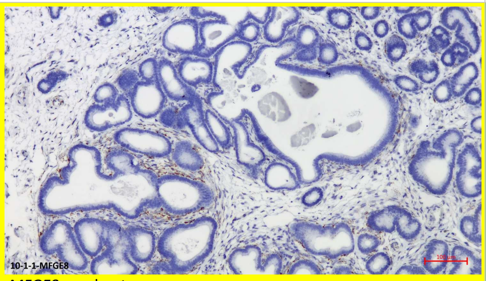 *MFGE8*, moderate	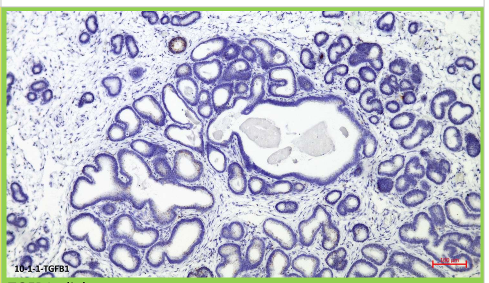 *TGFB1*, slight
10-1-2	4 to 10	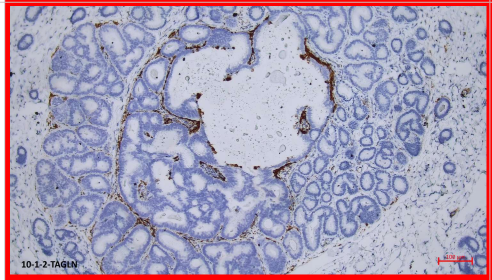 *TAGLN*, intense	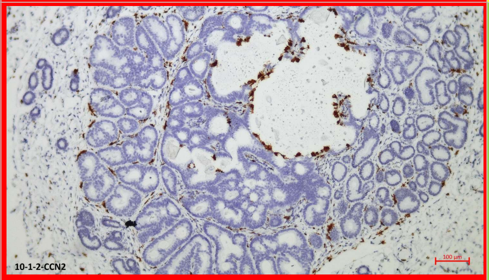 *CCN2*, intense	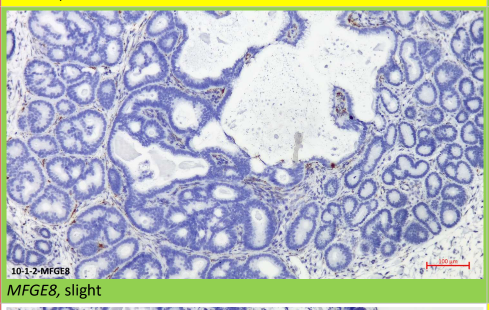 *MFGE8*, slight	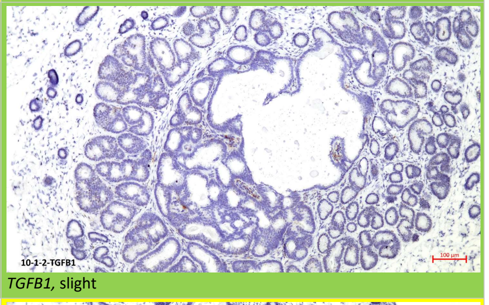 *TGFB1*, slight
10-1-3	4 to 10	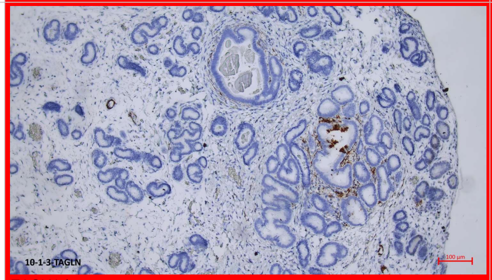 *TAGLN*, intense	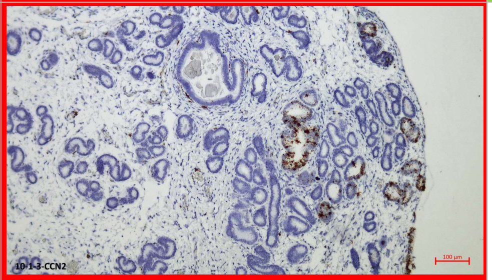 *CCN2*, intense	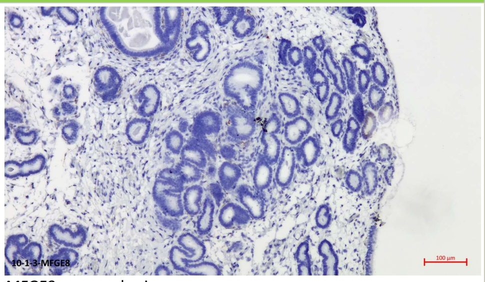 *MFGE8*, none or basic	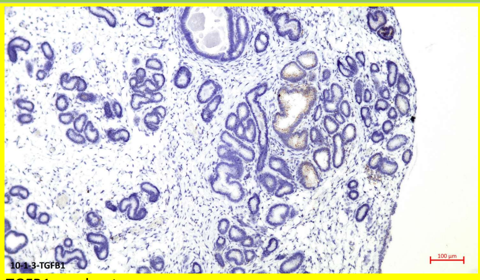 *TGFB1*, moderate
10-1-4	> 10	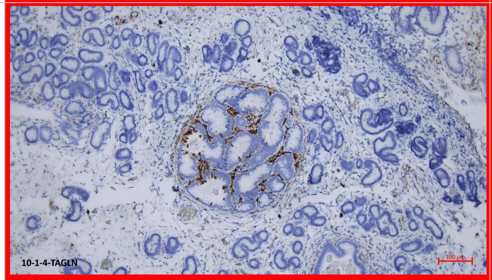 *TAGLN*, intense	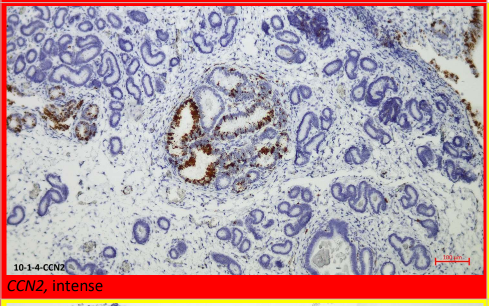 *CCN2*, intense	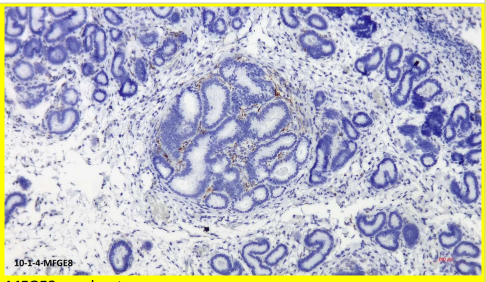 *MFGE8*, moderate	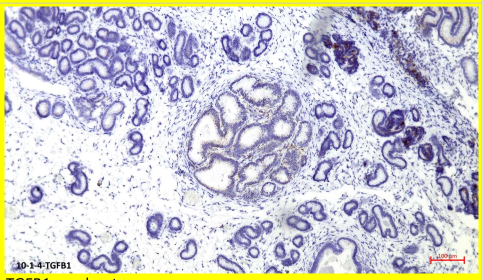 *TGFB1*, moderate
10-1-5	4 to 10	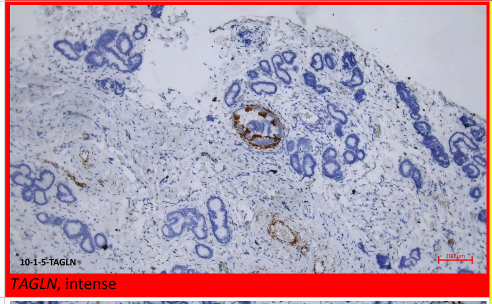 *TAGLN*, intense	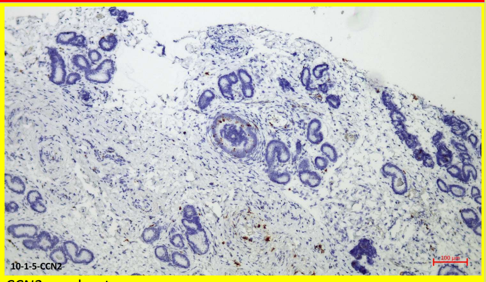 *CCN2*, moderate	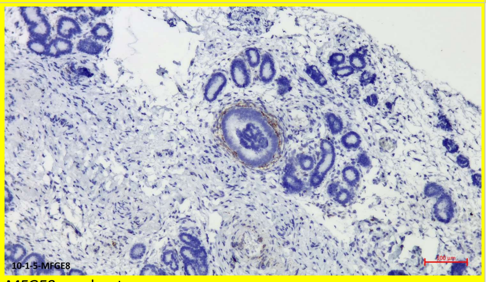 *MFGE8*, moderate	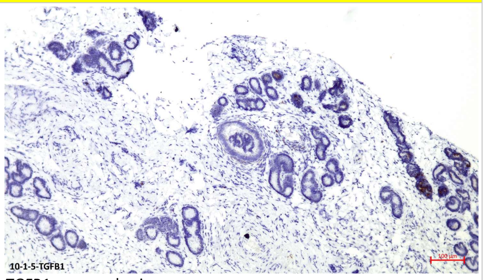 *TGFB1*, none or basic
10-1-6	1 to 3	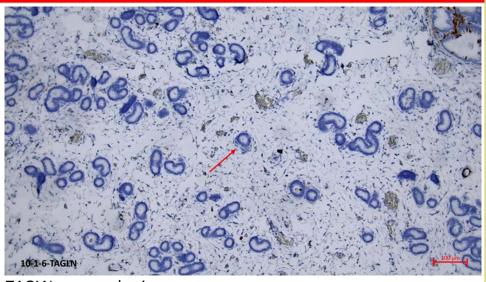 *TAGLN*, none or basic	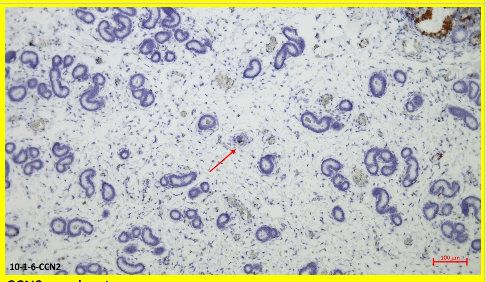 *CCN2*, moderate	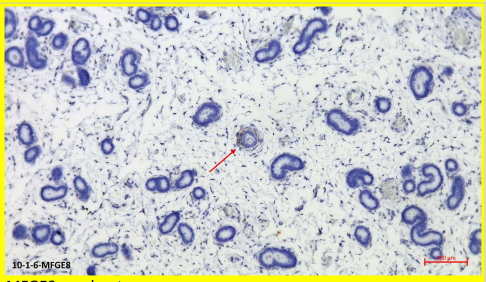 *MFGE8*, moderate	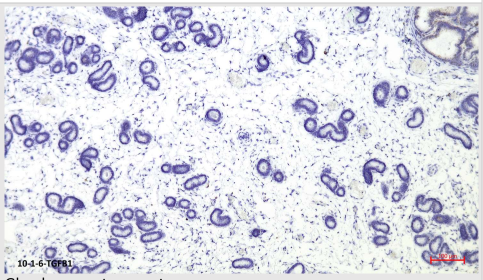 Gland area not present.
10-1-7	1 to 3	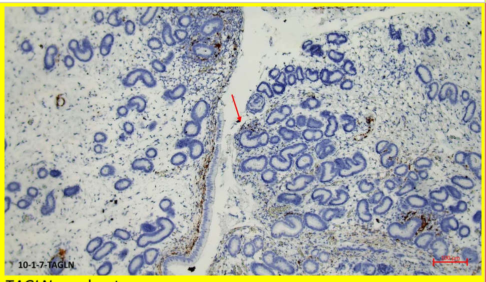 *TAGLN*, moderate	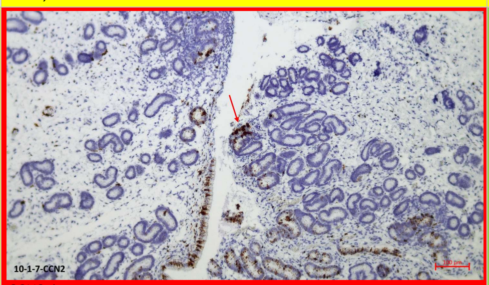 *CCN2*, intense	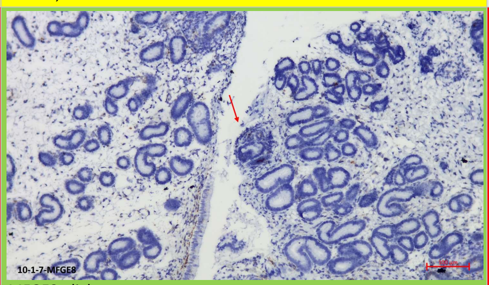 *MFGE8*, slight	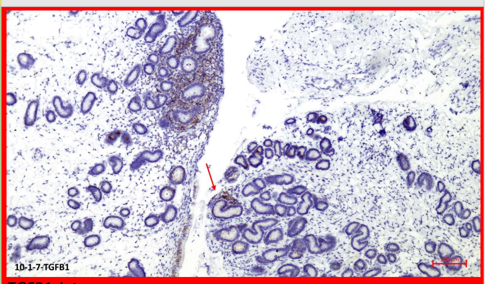 *TGFB1*, intense
10-1-8	4 to 10	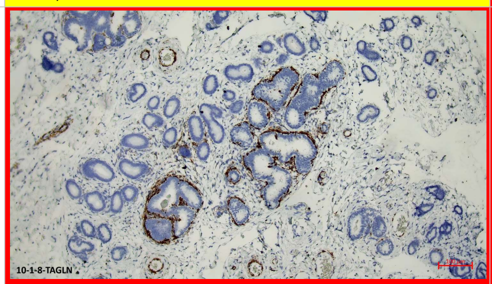 *TAGLN*, intense	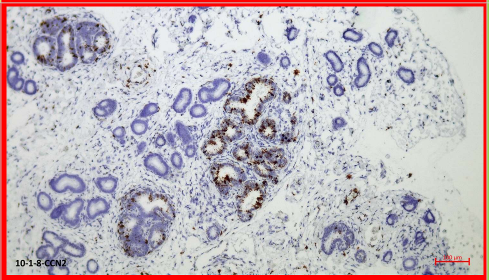 *CCN2*, intense	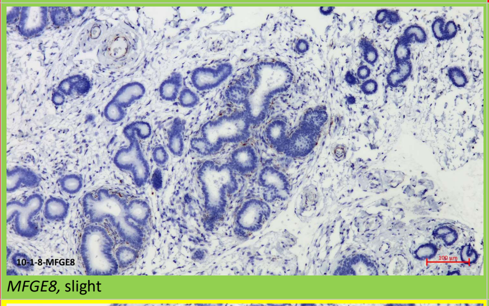 *MFGE8*, slight	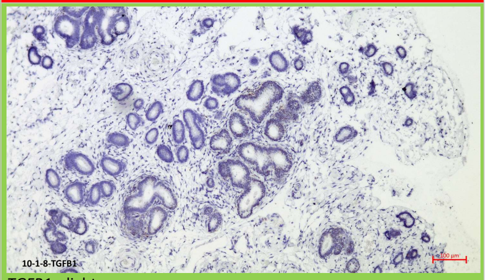 *TGFB1*, slight
10-1-9	1 to 3	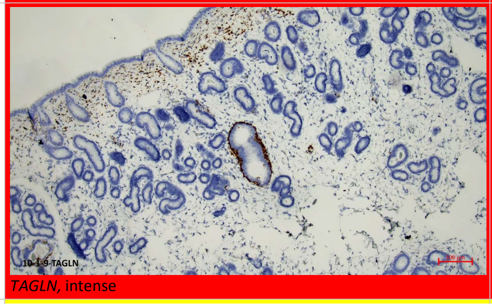 *TAGLN*, intense	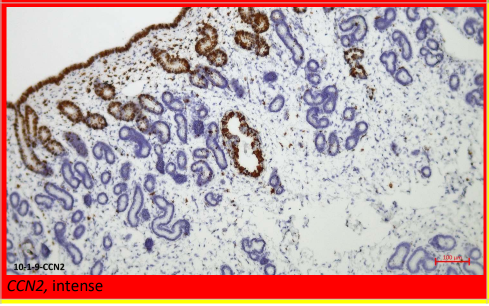 *CCN2*, intense	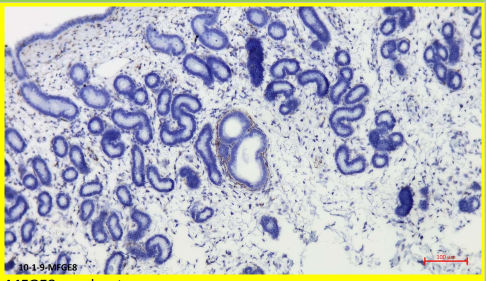 *MFGE8*, moderate	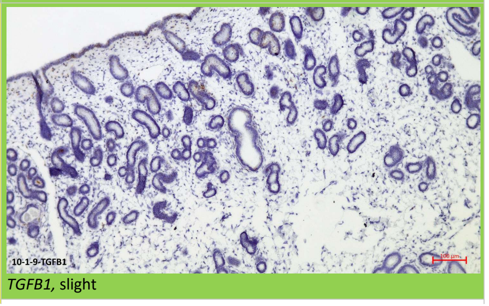 *TGFB1*, slight

10-1-10	1 to 3	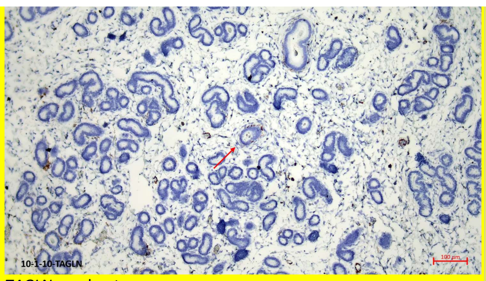 *TAGLN*, moderate	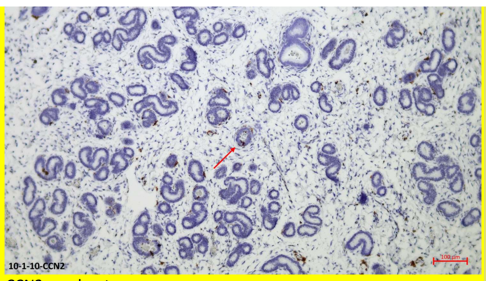 *CCN2*, moderate	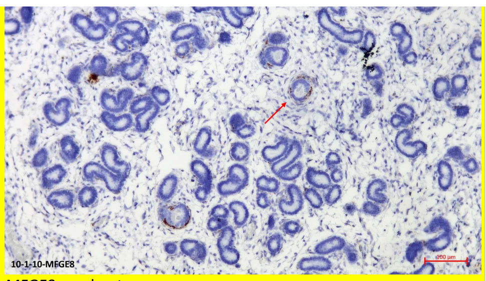 *MFGE8*, moderate	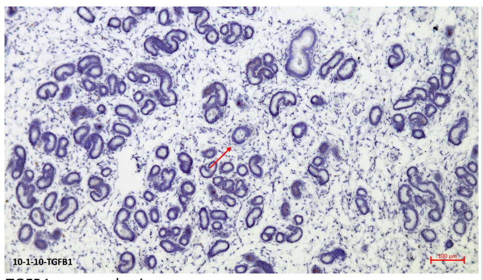 *TGFB1*, none or basic
10-1-11	1 to 3	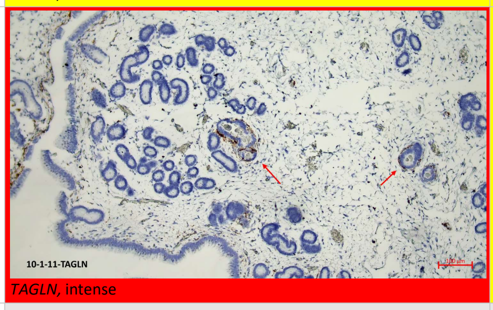 *TAGLN*, intense	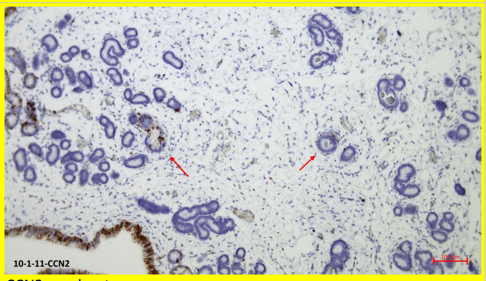 *CCN2*, moderate	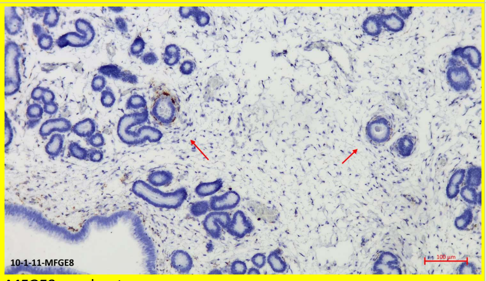 *MFGE8*, moderate	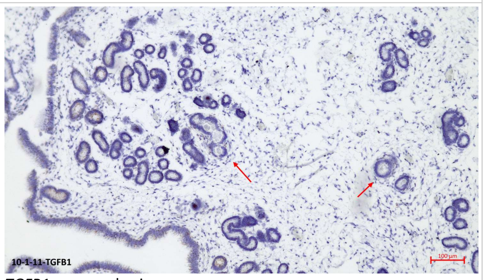 *TGFB1*, none or basic
10-1-12	none	Gland area not present.	Gland area not present.	Gland area not present.	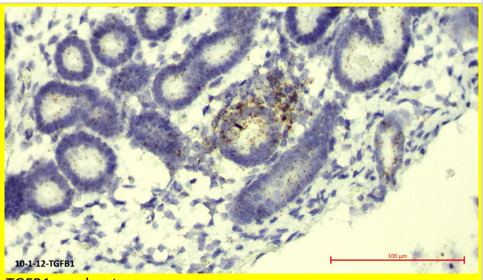 *TGFB1*, moderate

Mare 1-1

	Concentric layers	*TAGLN*	*CCN2*	*MFGE8*	*TGFB1*
1-1-A	4 to 10	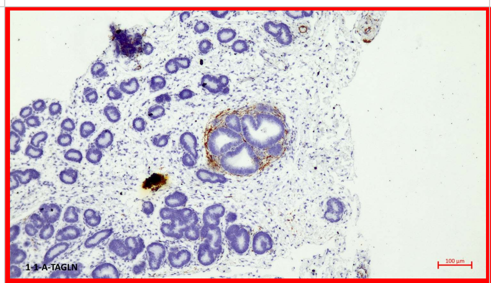<br>*TAGLN*, intense	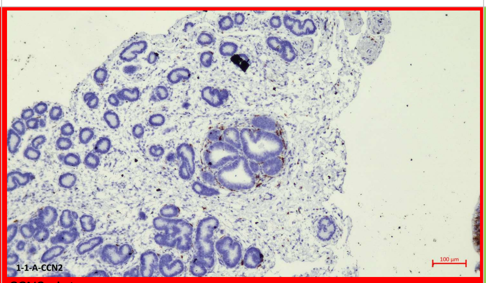<br>*CCN2*, intense	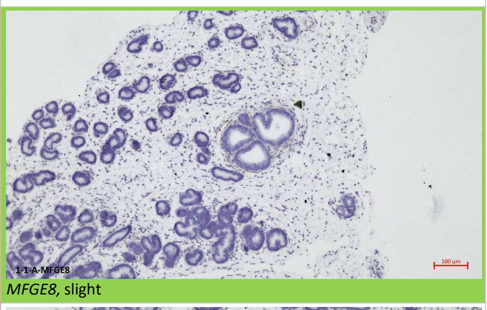<br>*MFGE8*, slight	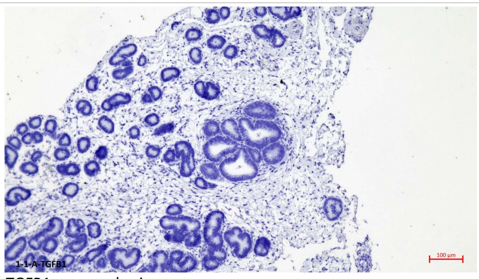<br>*TGFB1*, none or basic
1-1-B	4 to 10	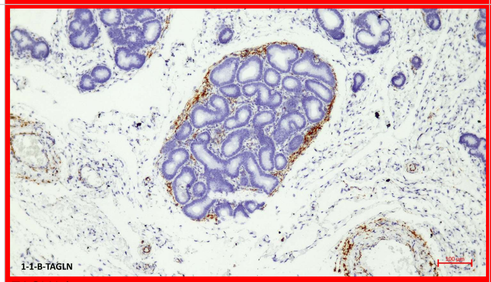<br>*TAGLN*, intense	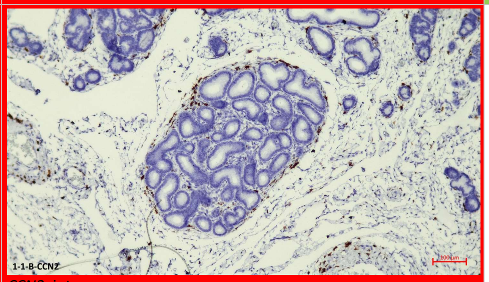<br>*CCN2*, intense	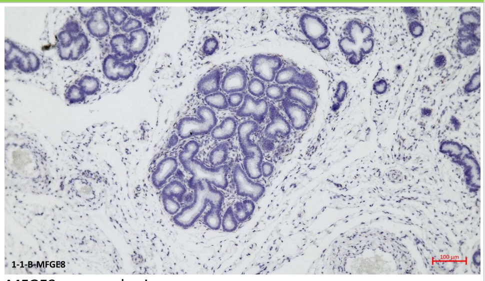<br>*MFGE8*, none or basic	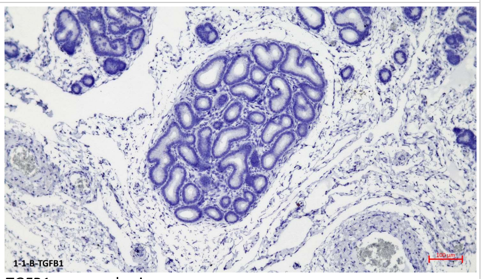<br>*TGFB1*, none or basic
1-1-C	>10	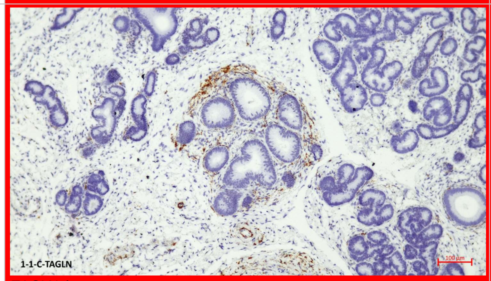<br>*TAGLN*, intense	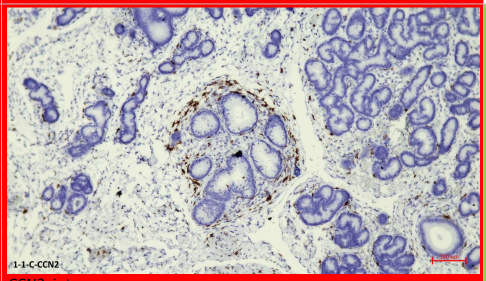<br>*CCN2*, intense	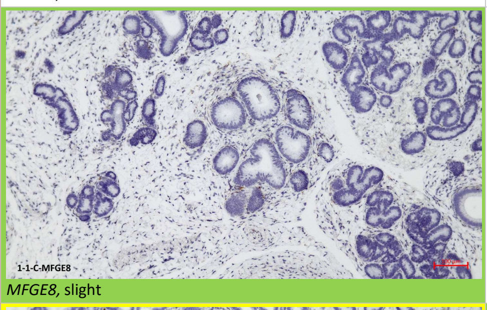<br>*MFGE8*, slight	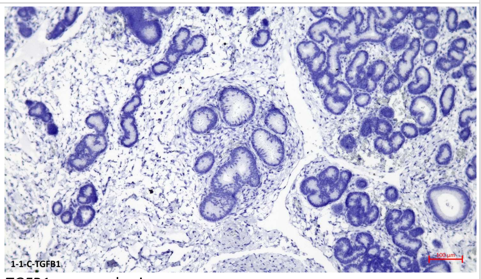<br>*TGFB1*, none or basic
1-1-D	4 to 10	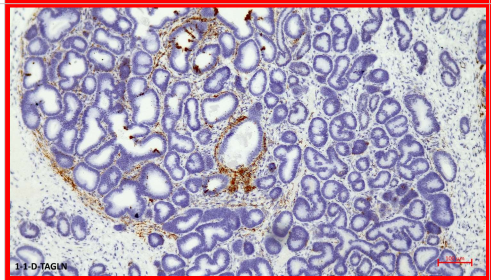<br>*TAGLN*, intense	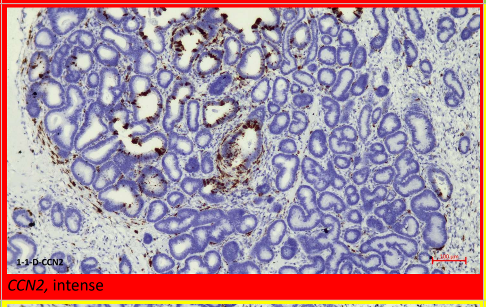<br>*CCN2*, intense	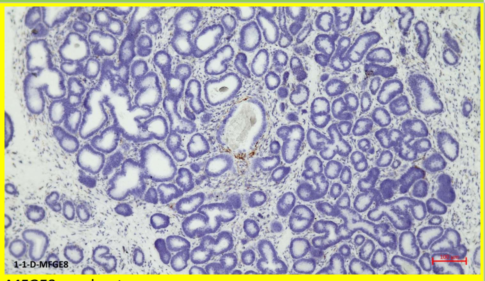<br>*MFGE8*, moderate	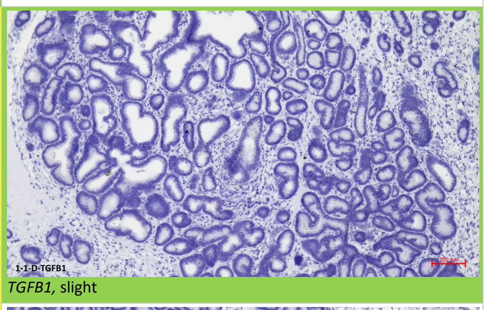<br>*TGFB1*, slight
1-1-E	none	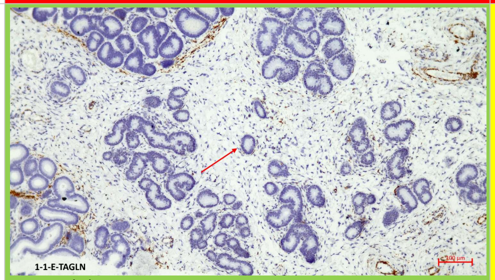<br>*TAGLN*, slight	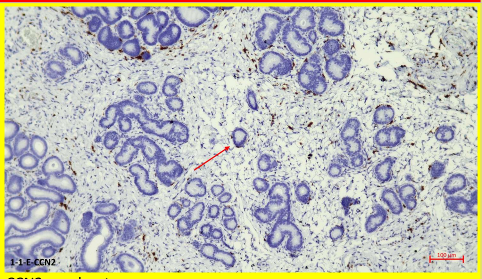<br>*CCN2*, moderate	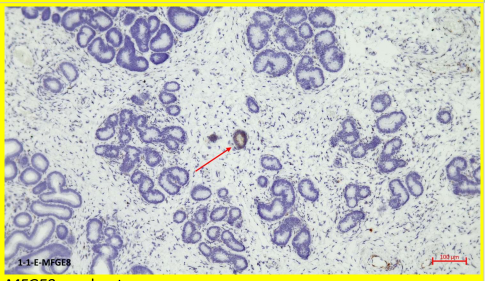<br>*MFGE8*, moderate	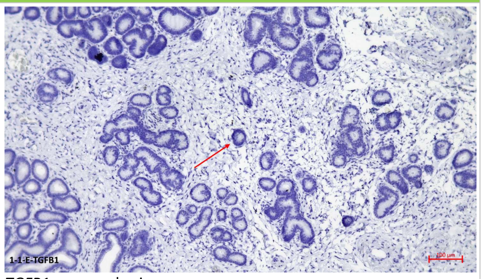<br>*TGFB1*, none or basic
1-1-F	4 to 10	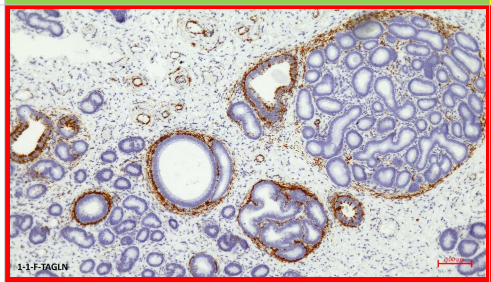<br>*TAGLN*, intense	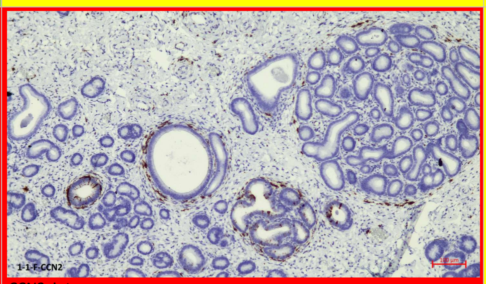<br>*CCN2*, intense	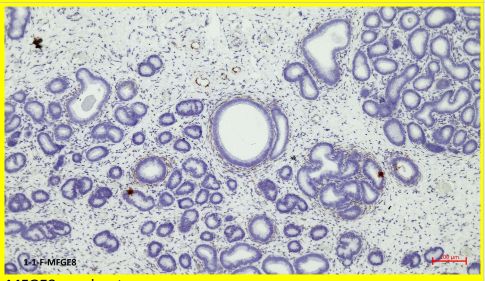<br>*MFGE8*, moderate	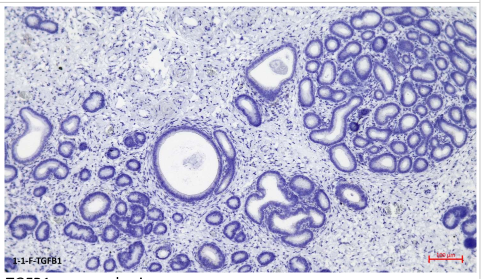<br>*TGFB1*, none or basic
1-1-G	4 to 10	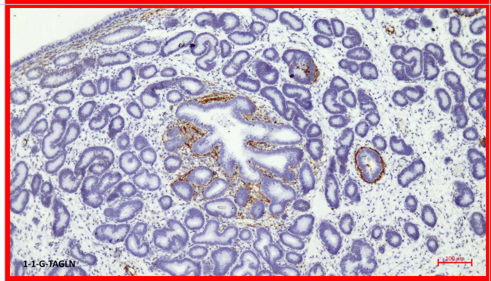<br>*TAGLN*, intense	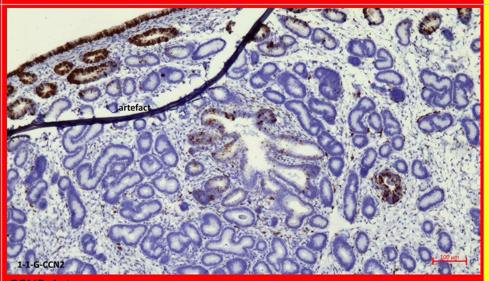<br>*CCN2*, intense	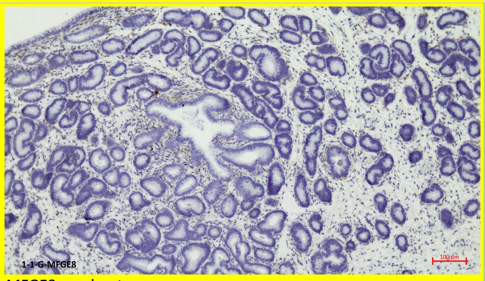<br>*MFGE8*, moderate	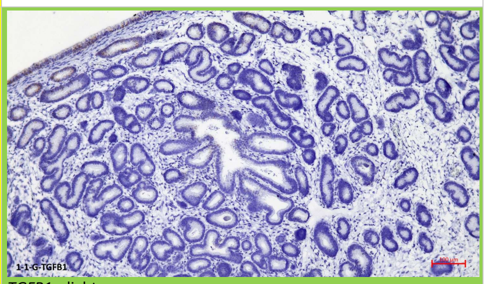<br>*TGFB1*, slight
1-1-H	4 to 10	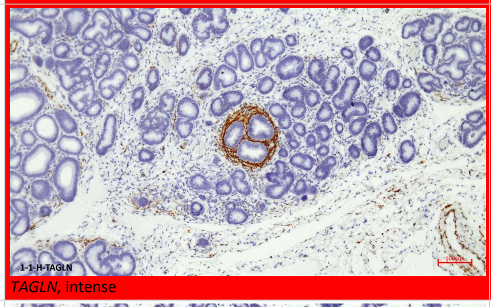<br>*TAGLN*, intense	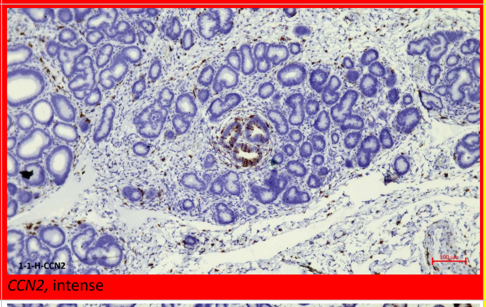<br>*CCN2*, intense	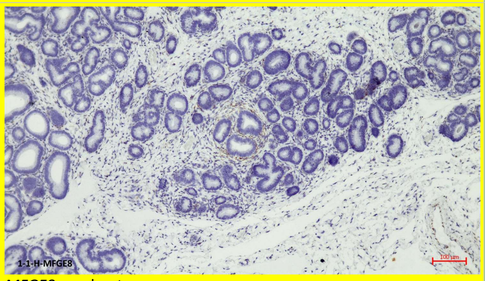<br>*MFGE8*, moderate	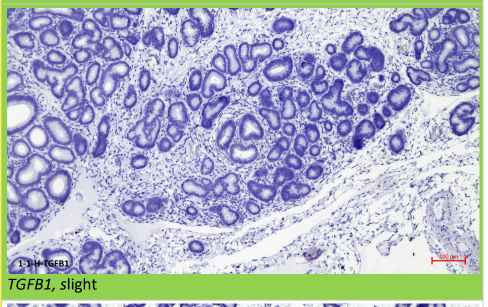<br>*TGFB1*, slight
1-1-I	none	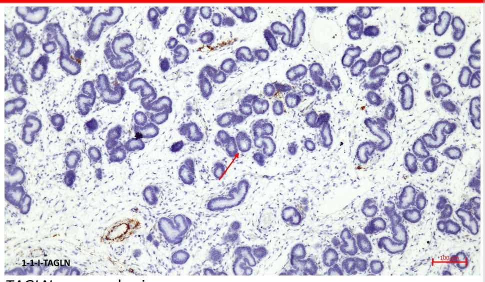<br>*TAGLN*, none or basic	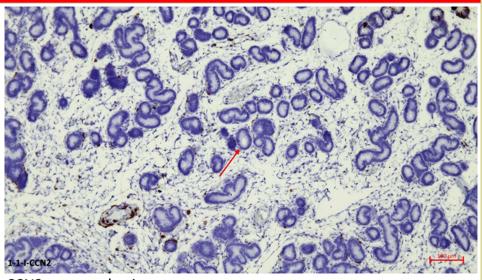<br>*CCN2*, none or basic	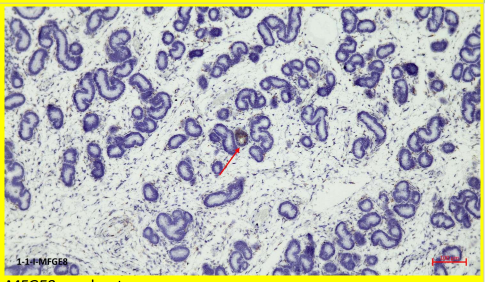<br>*MFGE8*, moderate	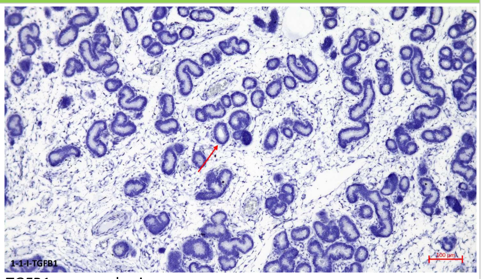<br>*TGFB1*, none or basic

1-1-J	4 to 10	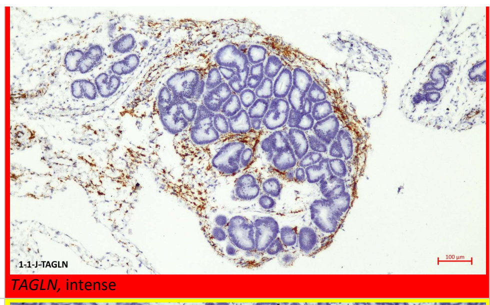 *TAGLN*, intense	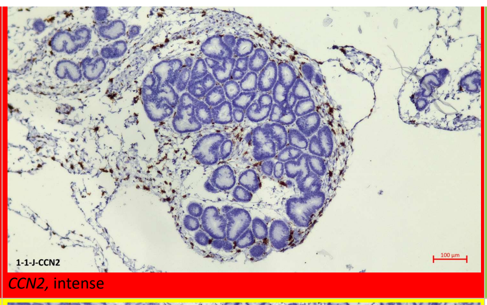 *CCN2*, intense	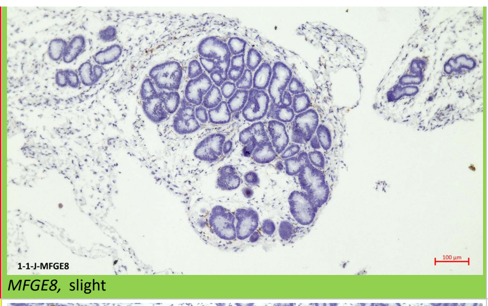 *MFGE8*, slight	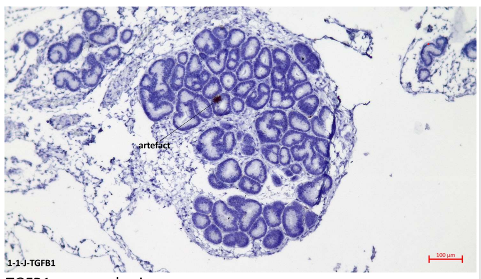 *TGFB1*, none or basic
1-1-K	4 to 10	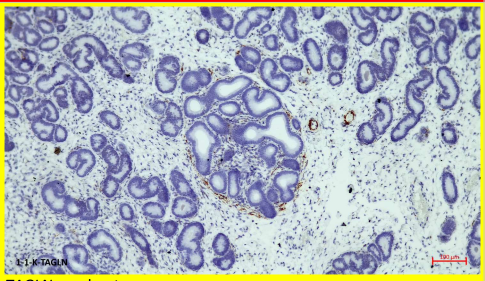 *TAGLN*, moderate	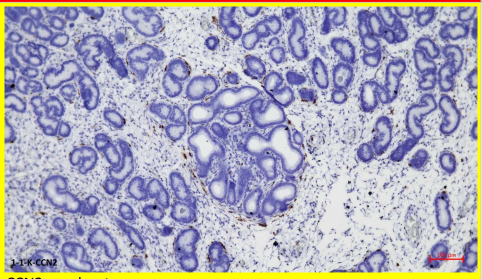 *CCN2*, moderate	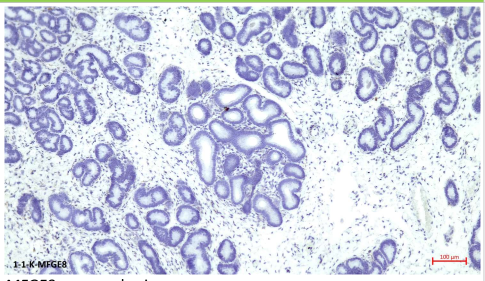 *MFGE8*, none or basic	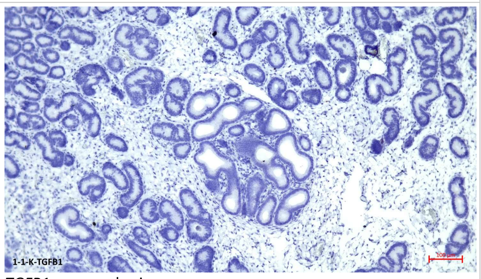 *TGFB1*, none or basic
1-1-L	1 to 3	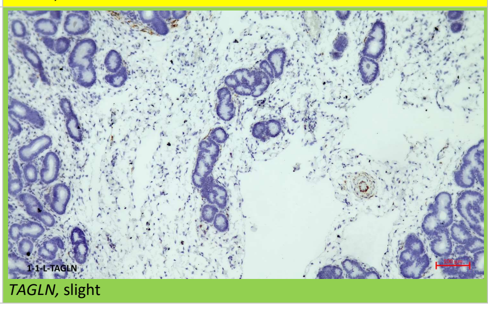 *TAGLN*, slight	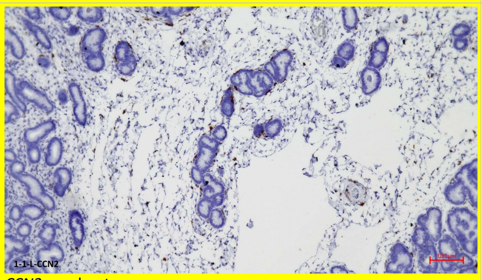 *CCN2*, moderate	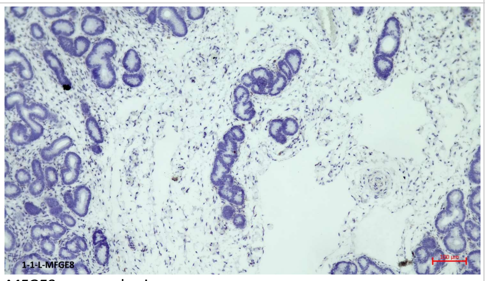 *MFGE8*, none or basic	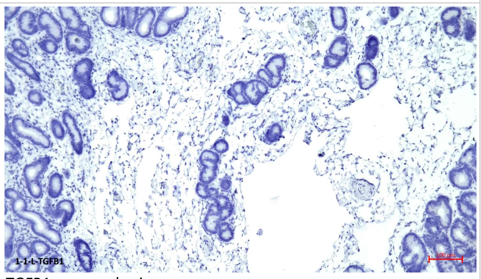 *TGFB1*, none or basic

Mare 24-1

	Concentric layers	*TAGLN*	*CCN2*	*MFGE8*	*TGFB1*
24-1-1	> 10	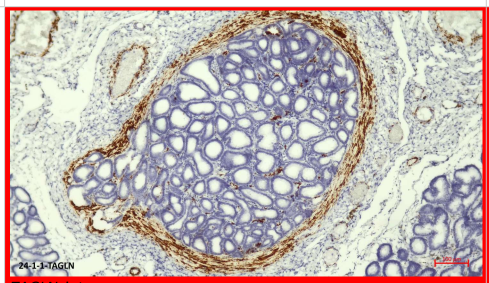 *TAGLN*, intense	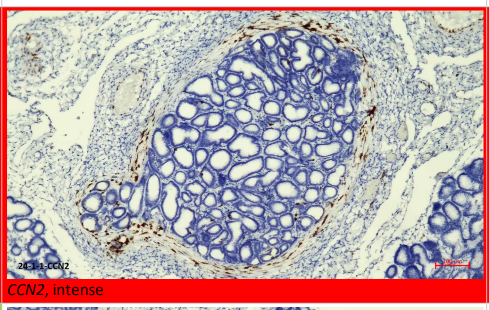 *CCN2*, intense	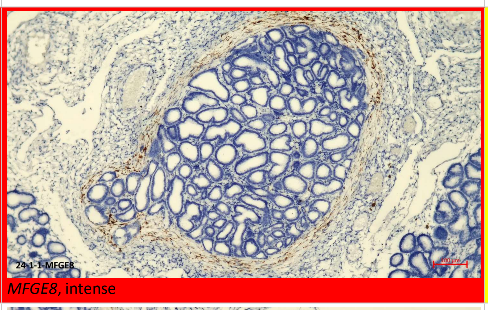 *MFGE8*, intense	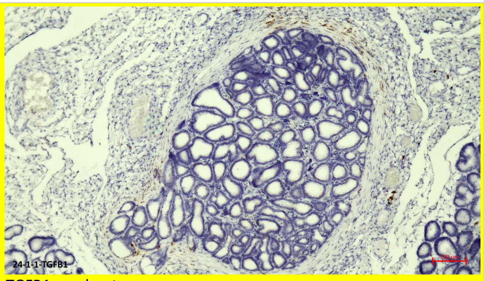 *TGFB1*, moderate
24-1-2	1 to 3	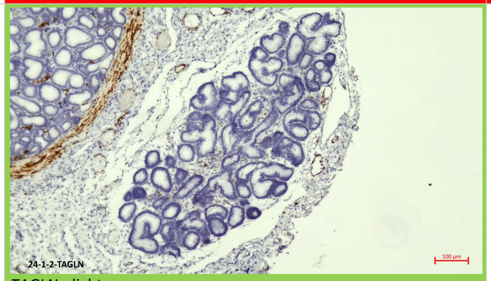 *TAGLN*, slight	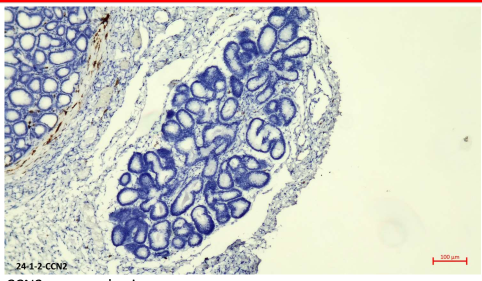 *CCN2*, none or basic	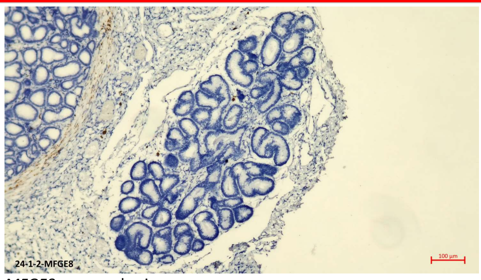 *MFGE8*, none or basic	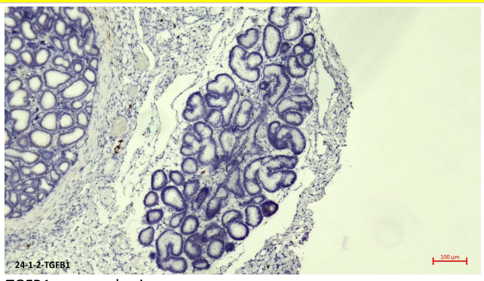 *TGFB1*, none or basic
24-1-3	1 to 3	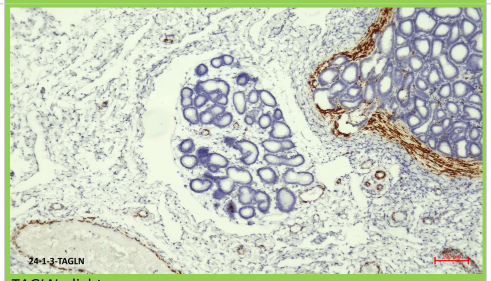 *TAGLN*, slight	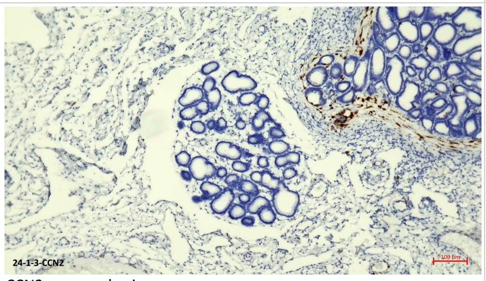 *CCN2*, none or basic	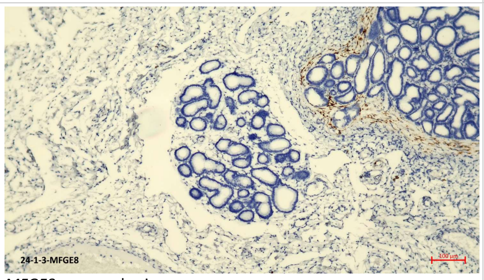 *MFGE8*, none or basic	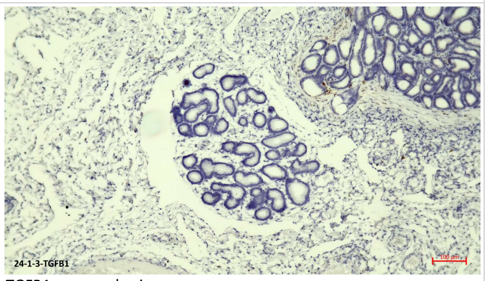 *TGFB1*, none or basic
24-1-4	4 to 10	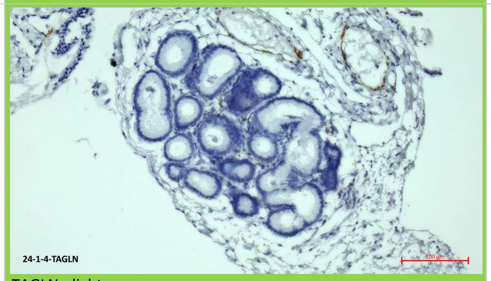 *TAGLN*, slight	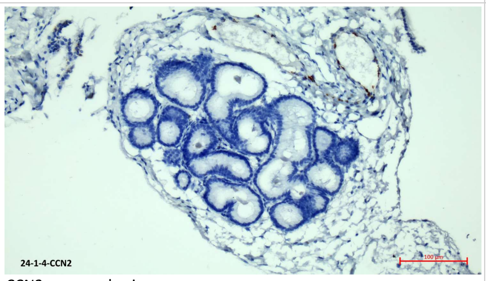 *CCN2*, none or basic	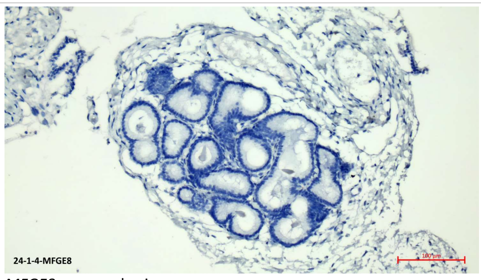 *MFGE8*, none or basic	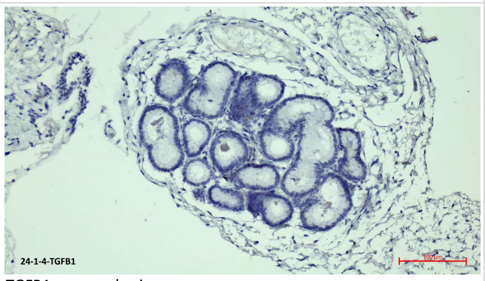 *TGFB1*, none or basic
24-1-6	1 to 3	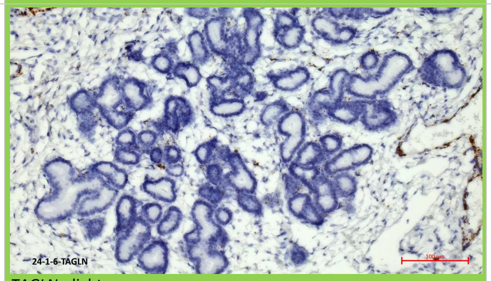 *TAGLN*, slight	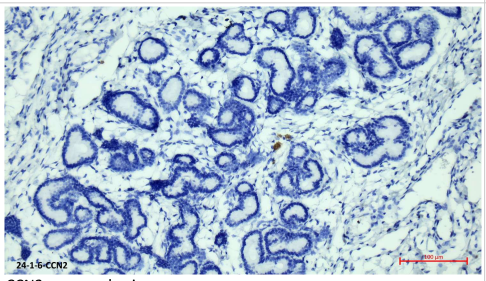 *CCN2*, none or basic	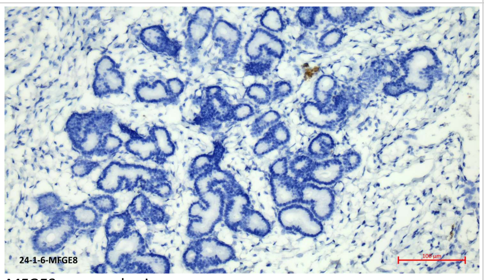 *MFGE8*, none or basic	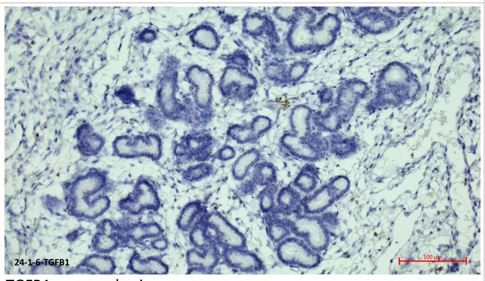 *TGFB1*, none or basic
24-1-8	4 to 10	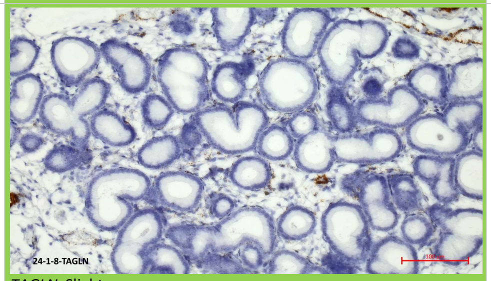 *TAGLN*, Slight	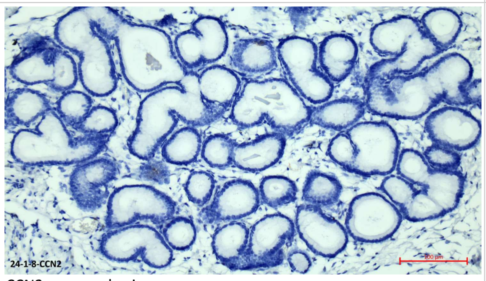 *CCN2*, none or basic	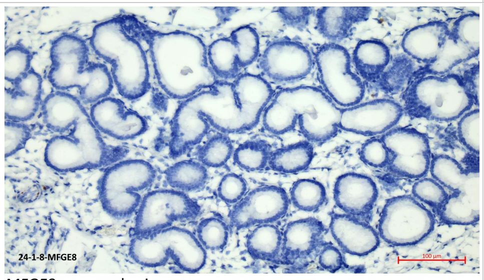 *MFGE8*, none or basic	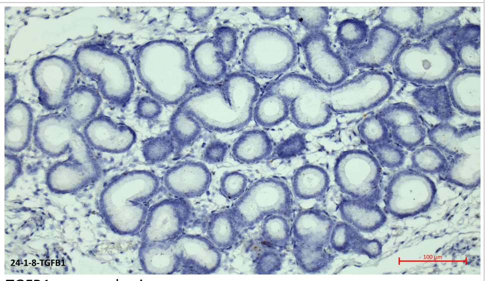 *TGFB1*, none or basic
24-1-11	1 to 3	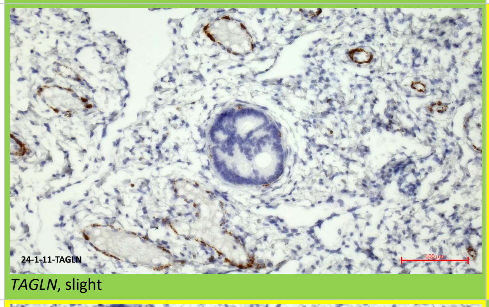 *TAGLN*, slight	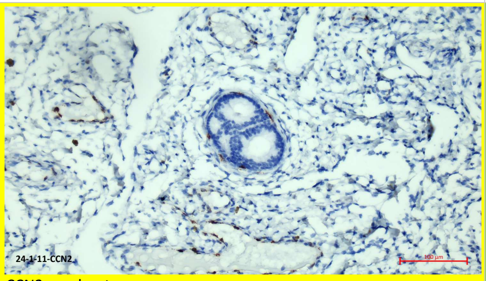 *CCN2*, moderate	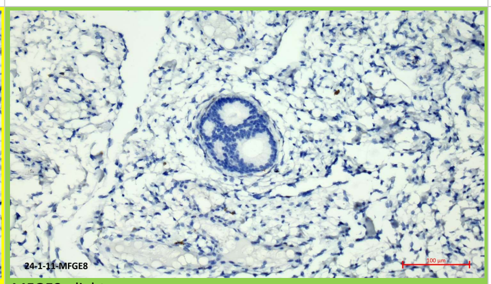 *MFGE8*, slight	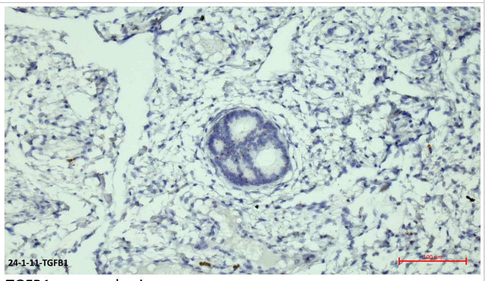 *TGFB1*, none or basic
24-1-12	none	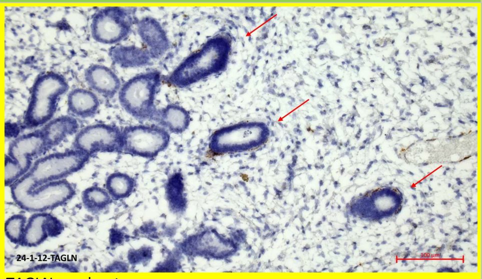 *TAGLN*, moderate	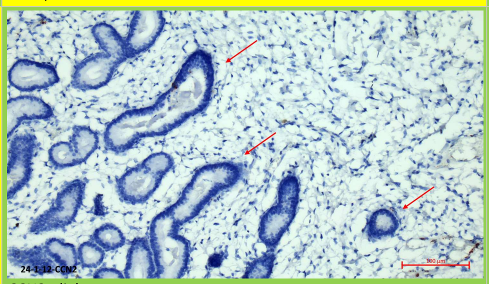 *CCN2*, slight	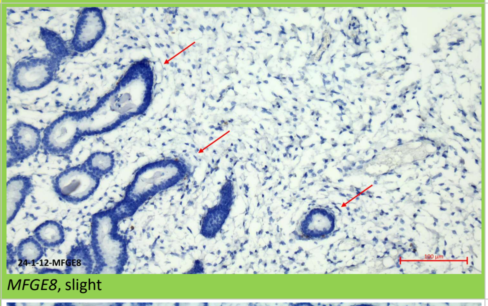 *MFGE8*, slight	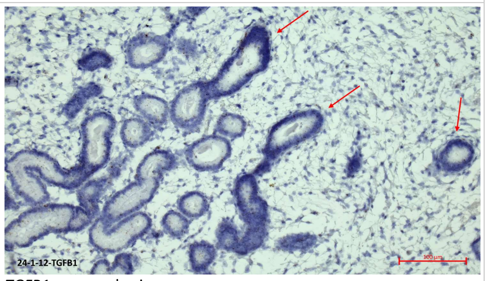 *TGFB1*, none or basic
24-1-14	none	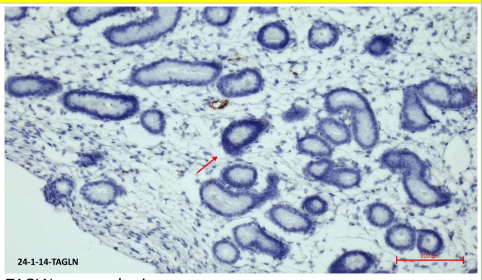 *TAGLN*, none or basic	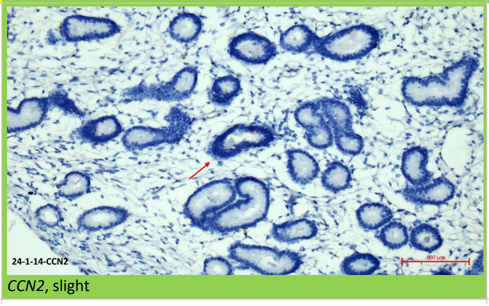 *CCN2*, slight	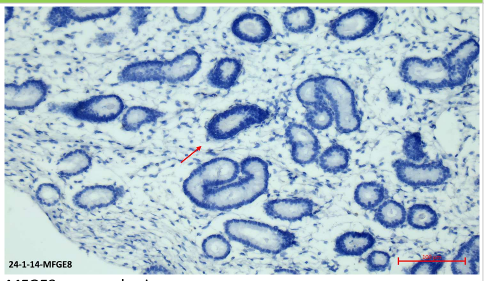 *MFGE8*, none or basic	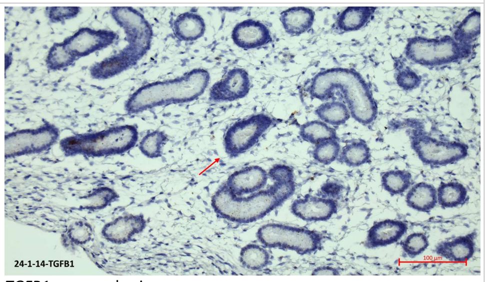 *TGFB1*, none or basic

		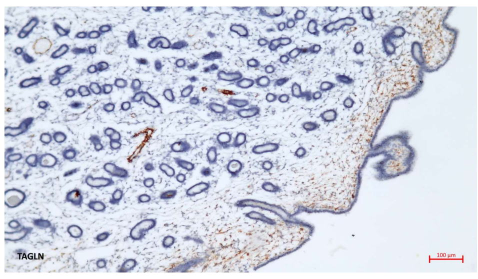 TAGLN<br>100 µm	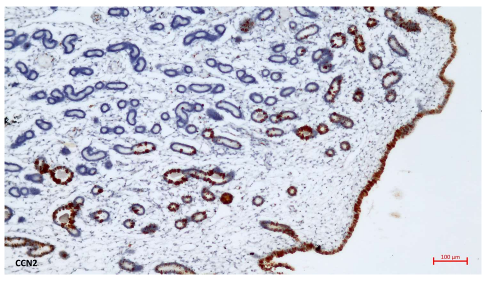 CCN2<br>100 µm	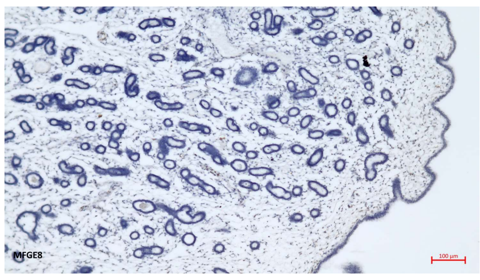 MFGE8<br>100 µm	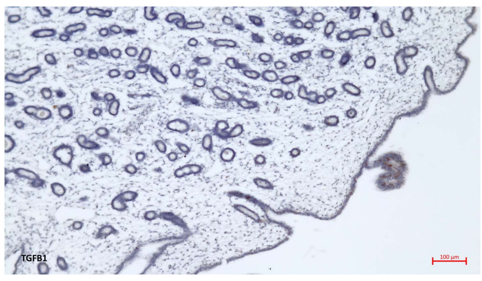 TGFB1<br>100 µm

Mare 23-1

	Concentric layers	*TAGLN*	*CCN2*	MFGE8	*TGFB1*
23-1-1	4 to 10	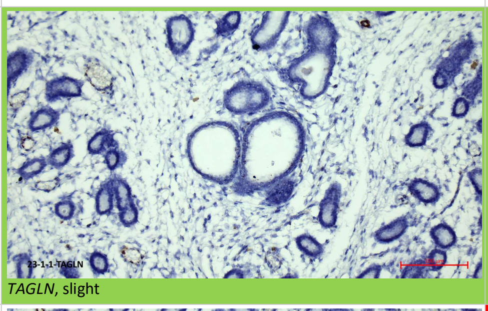 *TAGLN*, slight	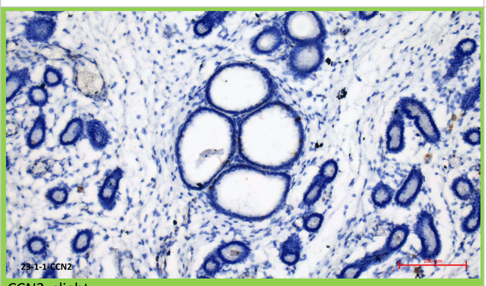 *CCN2*, slight	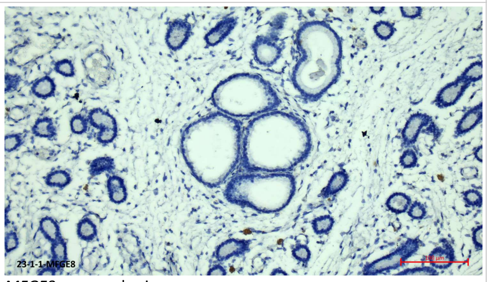 *MFGE8*, none or basic	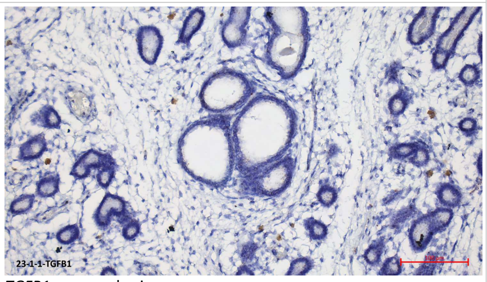 *TGFB1*, none or basic
23-1-2	More than 10	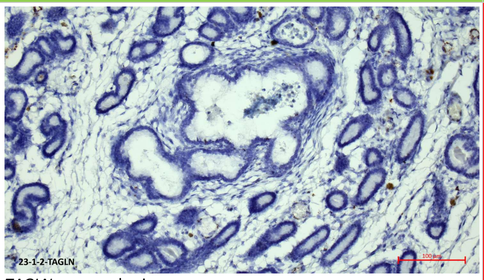 *TAGLN*, none or basic	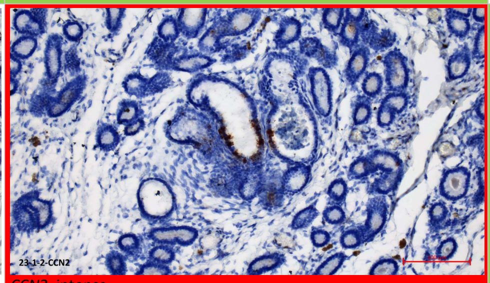 *CCN2*, intense	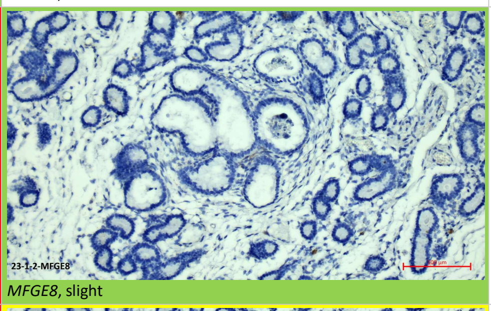 *MFGE8*, slight	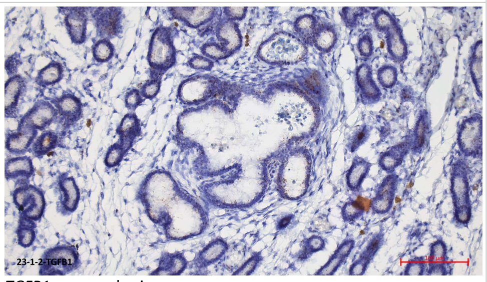 *TGFB1*, none or basic
23-1-3	1 to 3	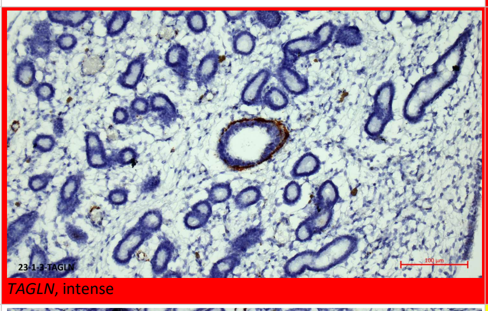 *TAGLN*, intense	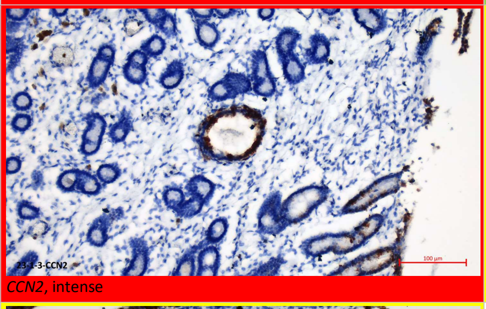 *CCN2*, intense	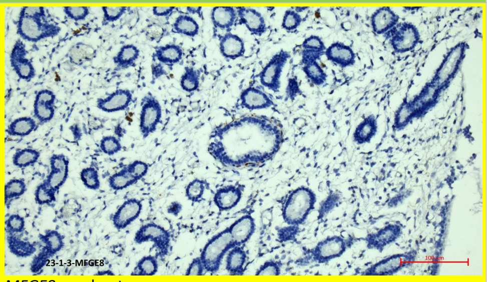 *MFGE8*, moderate	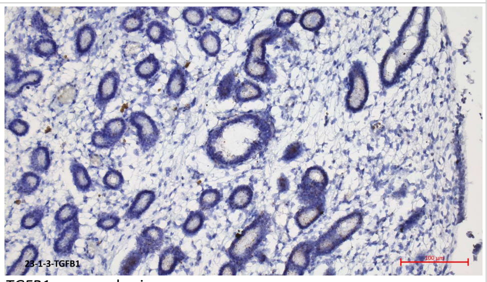 *TGFB1*, none or basic
23-1-4	1 to 3	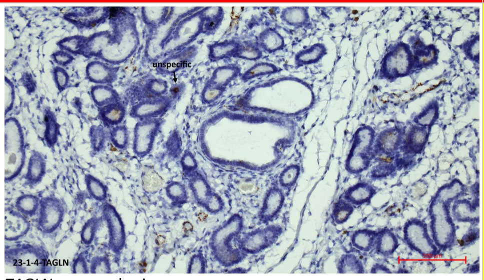 *TAGLN*, none or basic	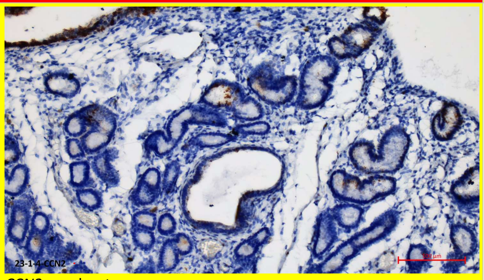 *CCN2*, moderate	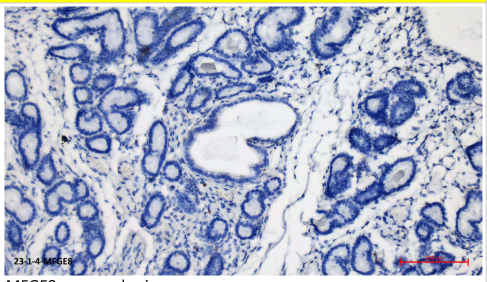 *MFGE8*, none or basic	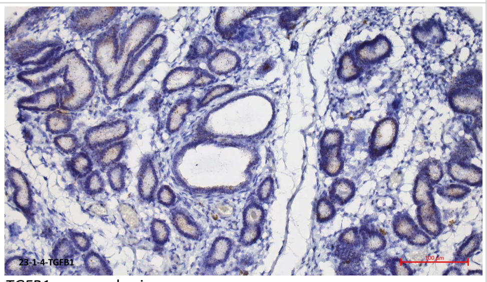 *TGFB1*, none or basic
23-1-5	none	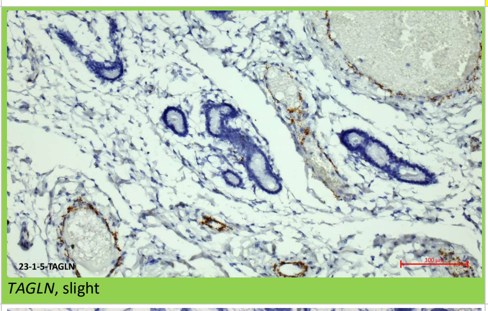 *TAGLN*, slight	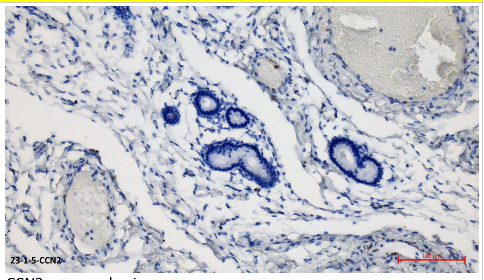 *CCN2*, none or basic	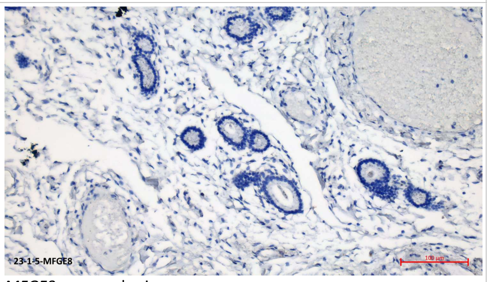 *MFGE8*, none or basic	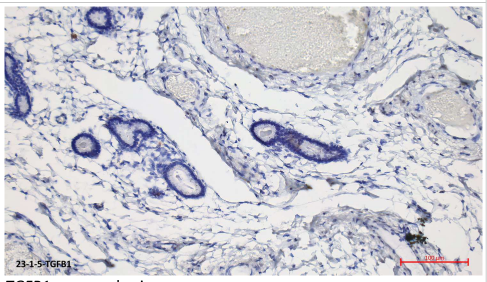 *TGFB1*, none or basic
		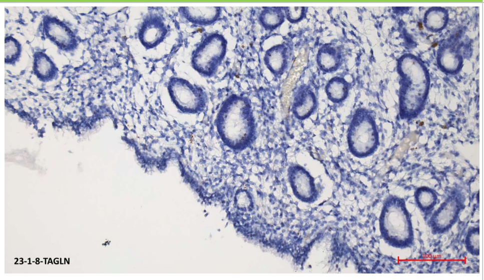	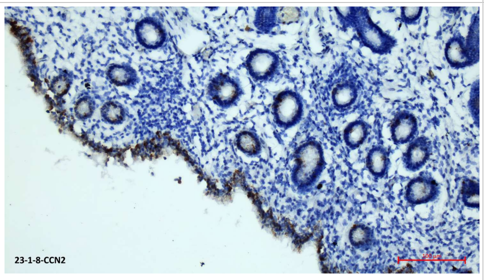	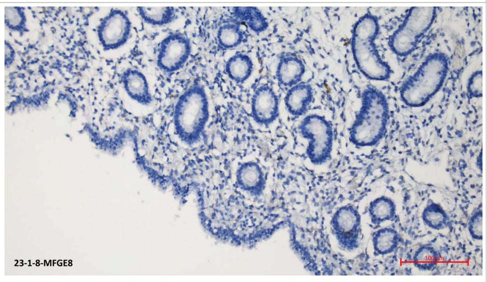	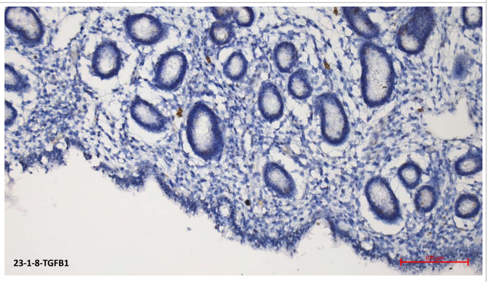

Mare 12-2

	Concentric layers	*TAGLN*	*CCN2*	*MFGE8*
12-2-1	1 to 3	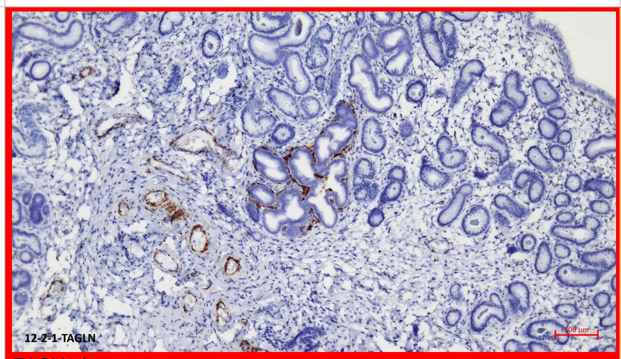 *TAGLN*, intense	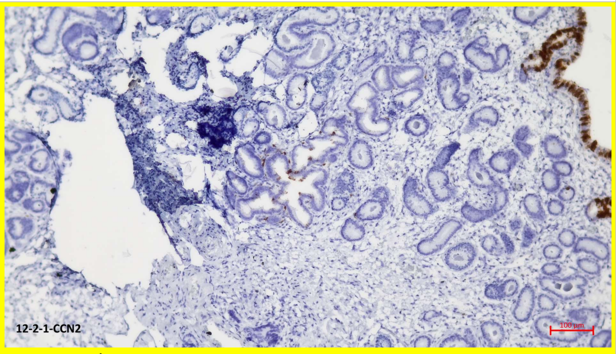 *CCN2*, moderate	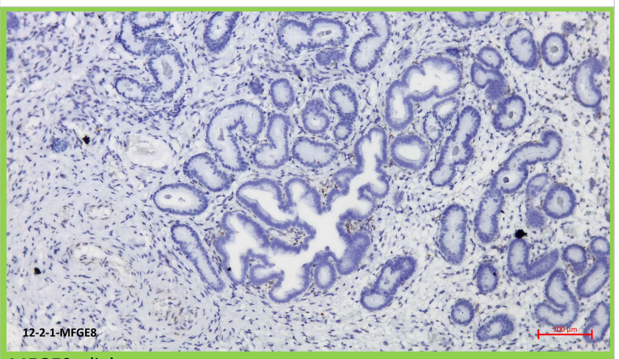 *MFGE8*, slight
12-2-2	1 to 3	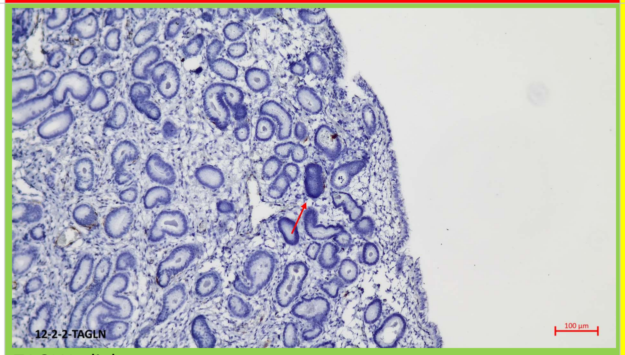 *TAGLN*, slight	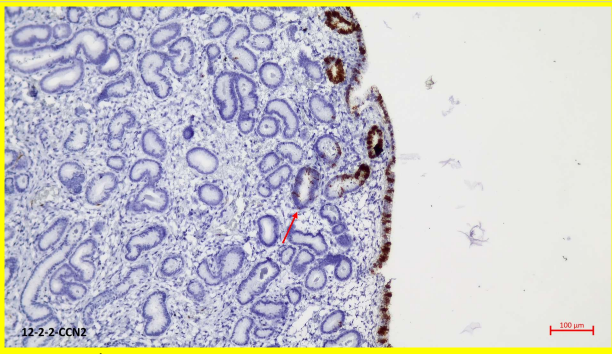 *CCN2*, moderate	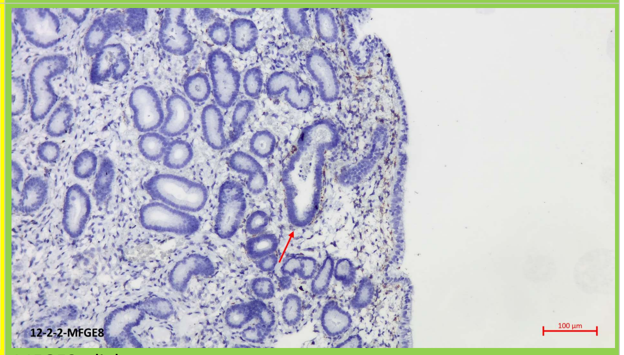 *MFGE8*, slight
12-2-3	4 to 10	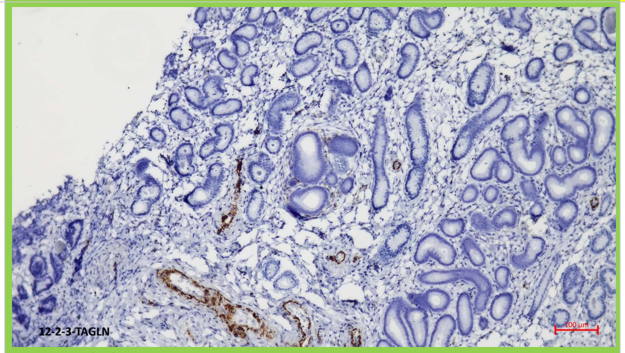 *TAGLN*, slight	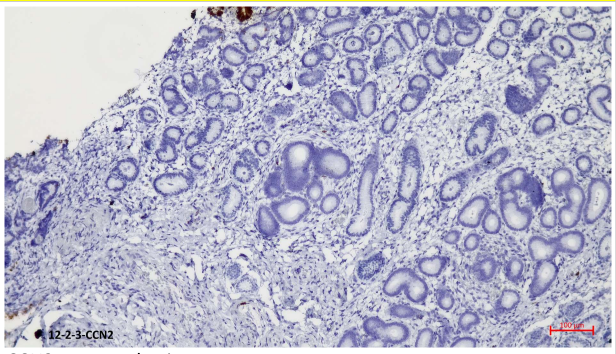 *CCN2*, none or basic	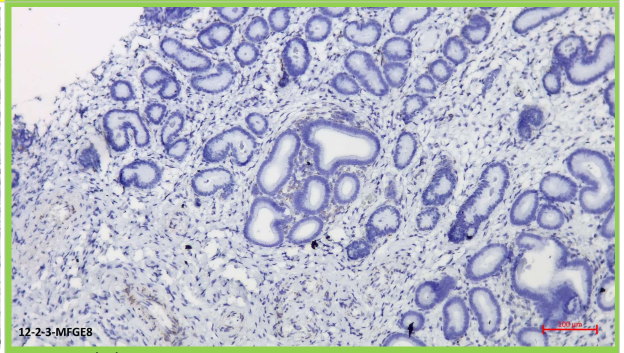 *MFGE8*, slight
12-2-4	4 to 10	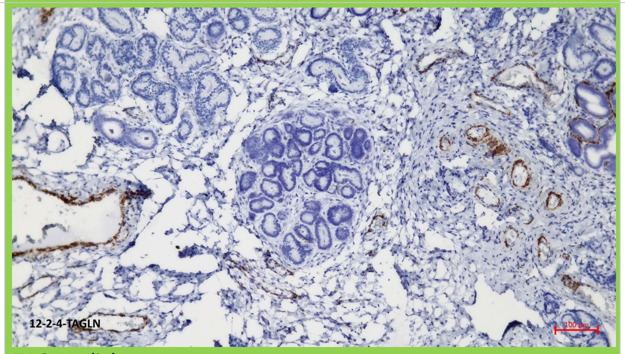 *TAGLN*, slight	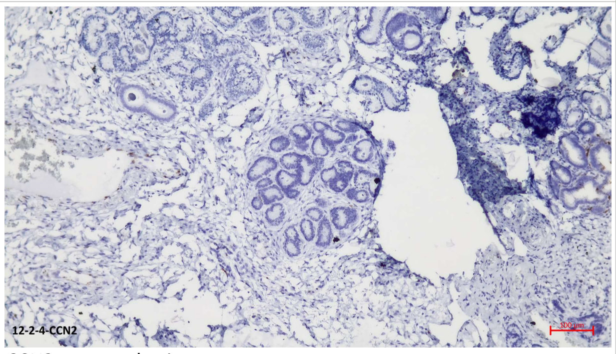 *CCN2*, none or basic	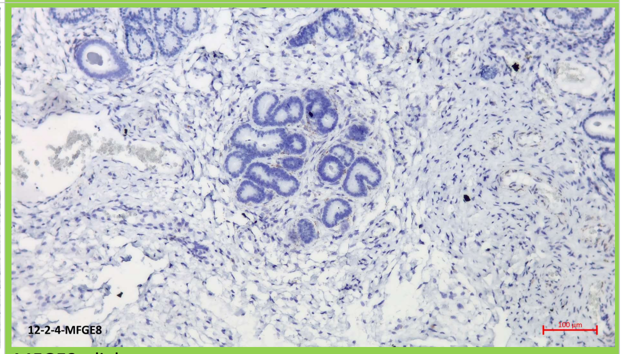 *MFGE8*, slight
12-2-5	1 to 3	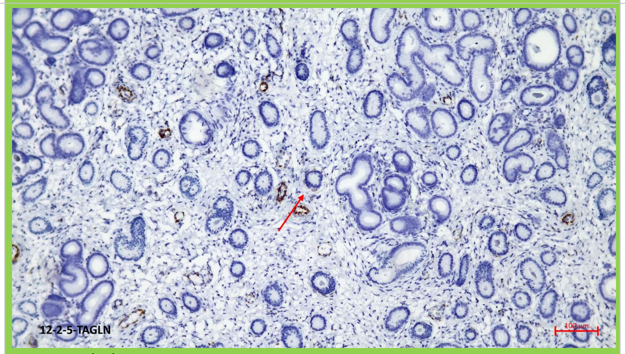 *TAGLN*, slight	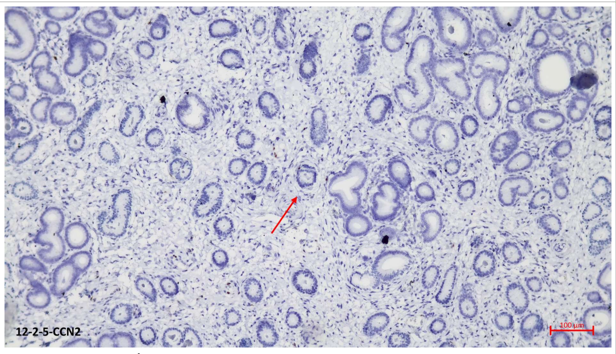 *CCN2*, none or basic	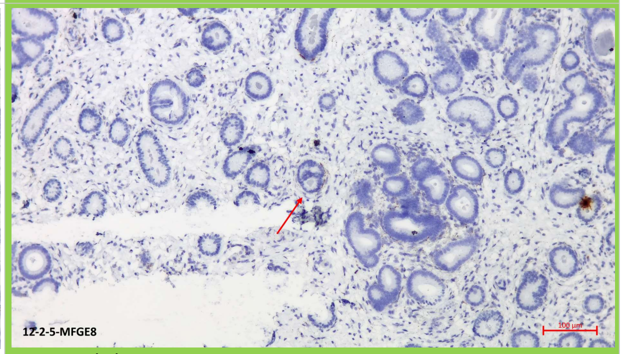 *MFGE8*, slight
12-2-6	none	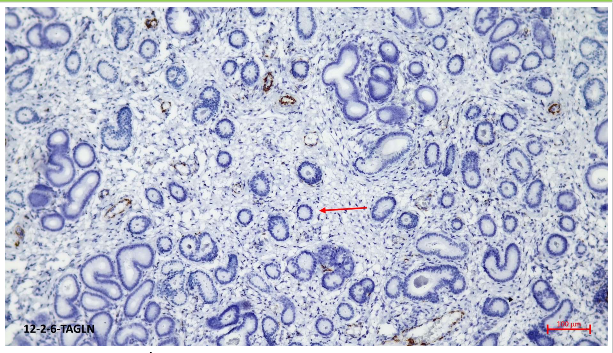 *TAGLN*, none or basic	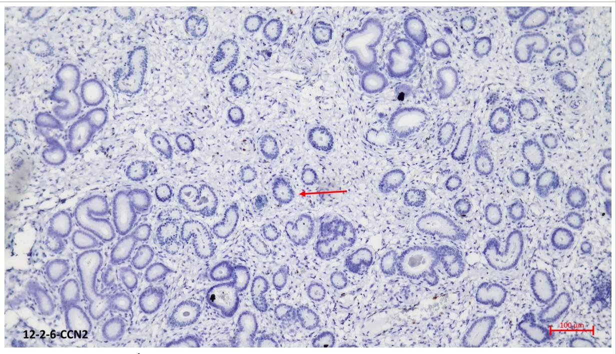 *CCN2*, none or basic	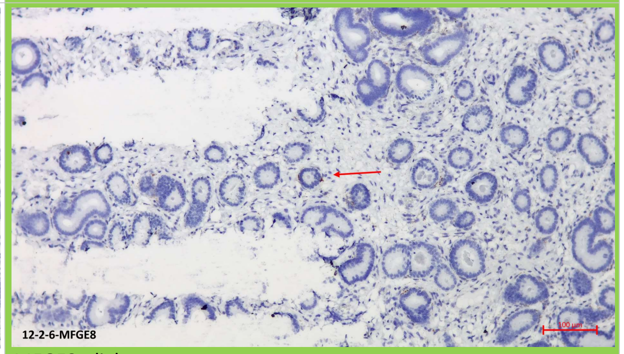 *MFGE8*, slight
12-2-7	1 to 3	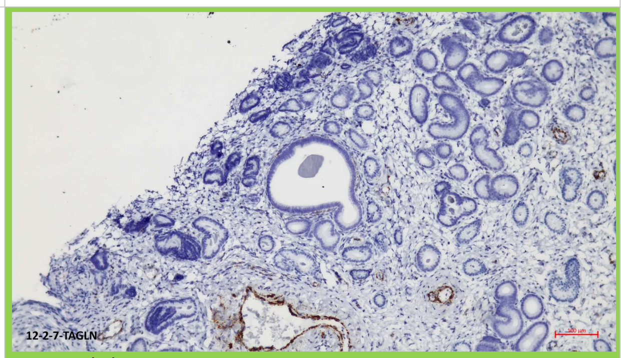 *TAGLN*, slight	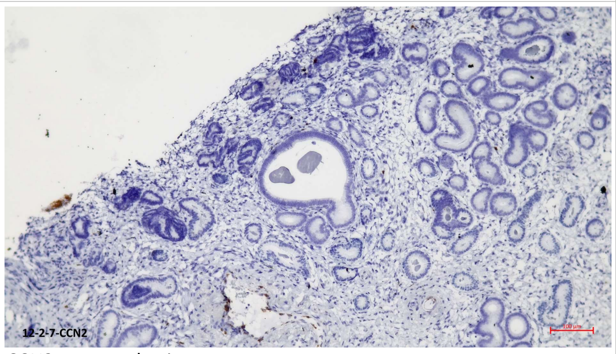 *CCN2*, none or basic	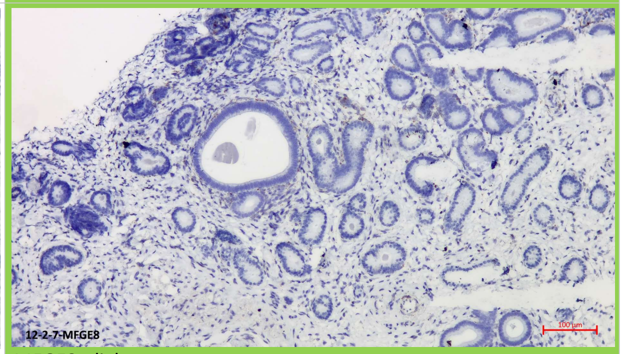 *MFGE8*, slight

12-2-8	1 to 3	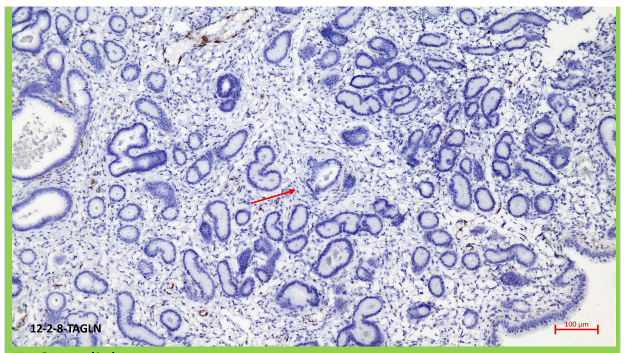 *TAGLN*, slight	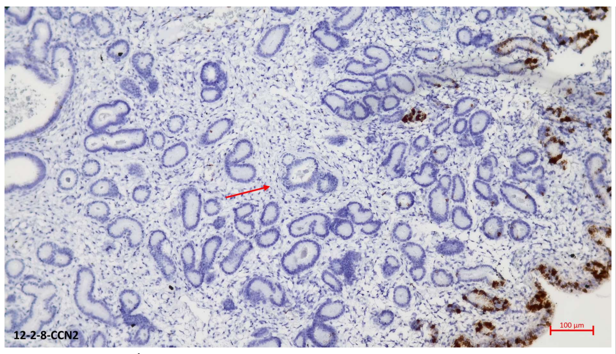 *CCN2*, none or basic	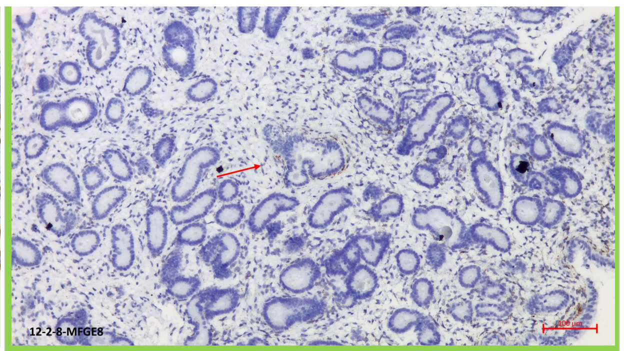 *MFGE8*, slight
12-2-9	4 to 10	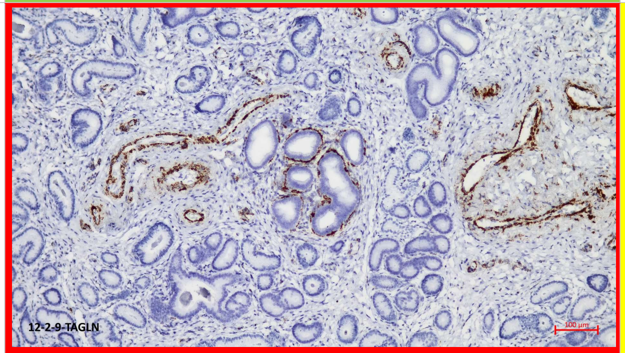 *TAGLN*, intense	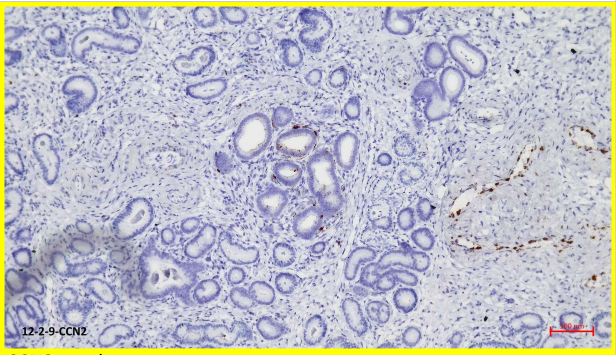 *CCN2*, moderate	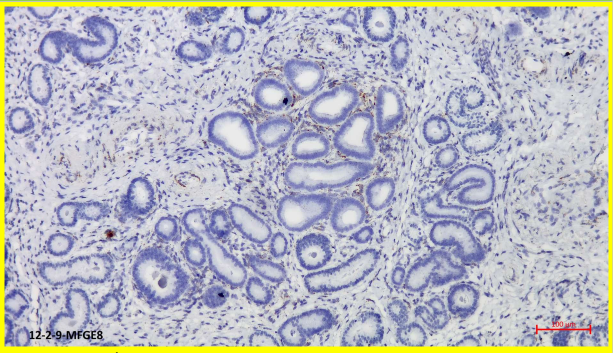 *MFGE8*, moderate
12-2-10	4 to 10	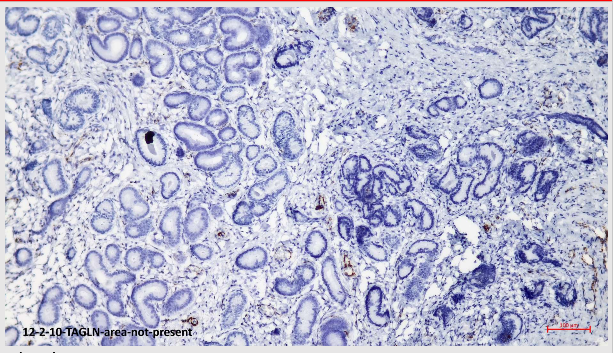 Gland area not present.	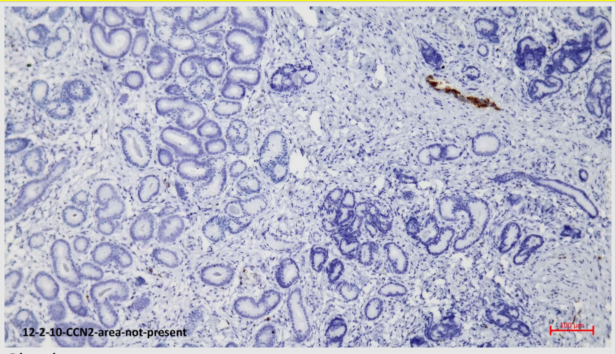 Gland area not present.	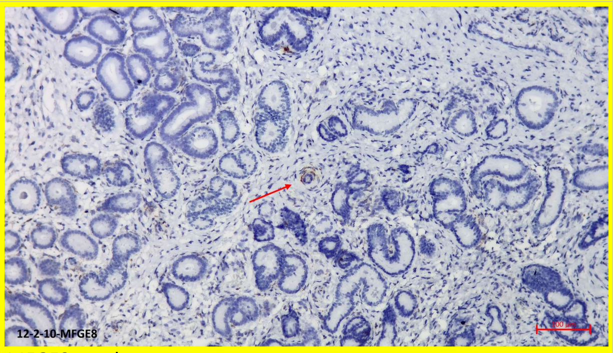 *MFGE8*, moderate

Mare 19-2

	Concentric layers	*TAGLN*	*CCN2*	*MFGE8*	*TGFB1*
19-2-1	4 to 10	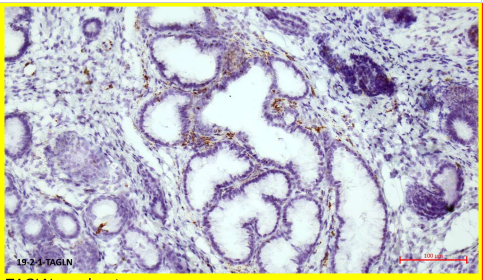<br>*TAGLN*, moderate	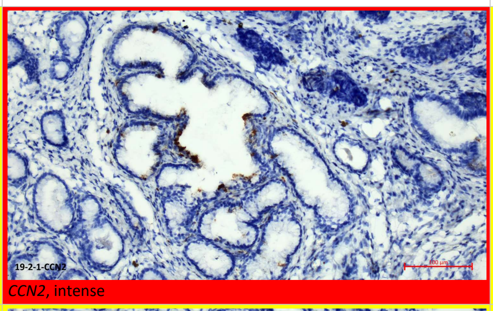<br>*CCN2*, intense	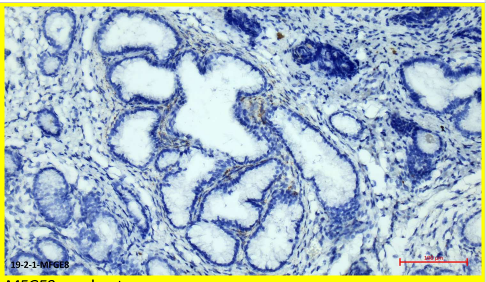<br>*MFGE8*, moderate	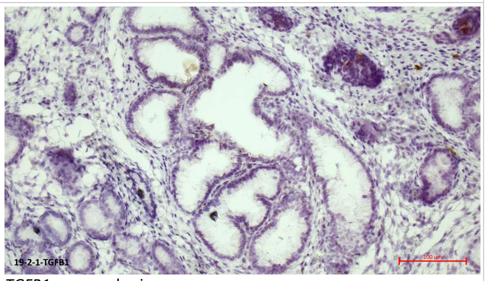<br>*TGFB1*, none or basic
19-2-2	4 to 10	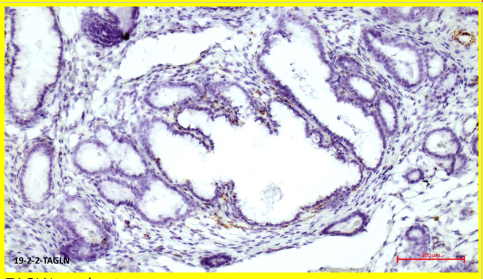<br>*TAGLN*, moderate	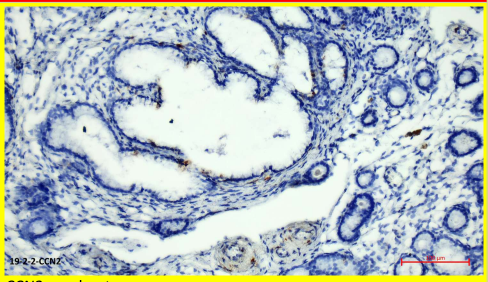<br>*CCN2*, moderate	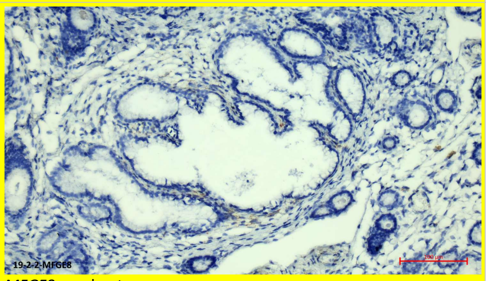<br>*MFGE8*, moderate	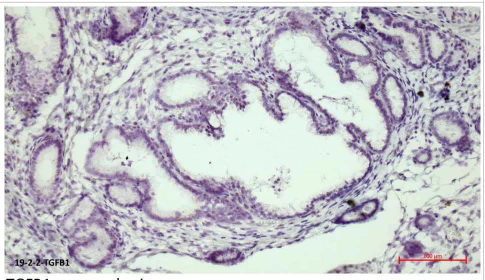<br>*TGFB1*, none or basic
19-2-3	1 to 3	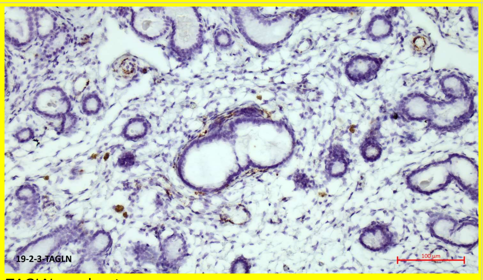<br>*TAGLN*, moderate	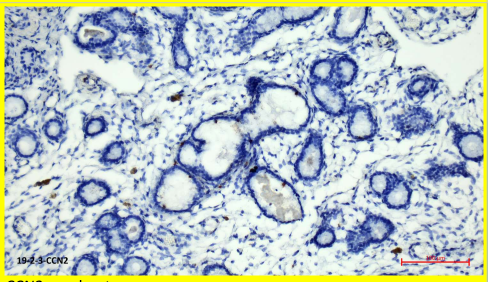<br>*CCN2*, moderate	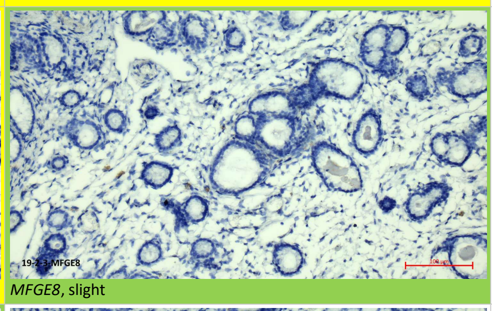<br>*MFGE8*, slight	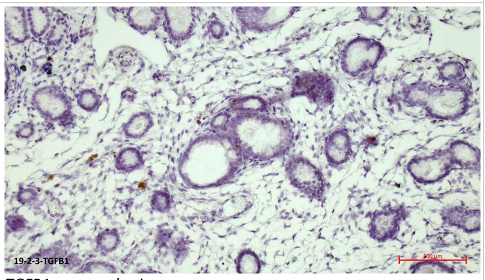<br>*TGFB1*, none or basic
19-2-4	1 to 3	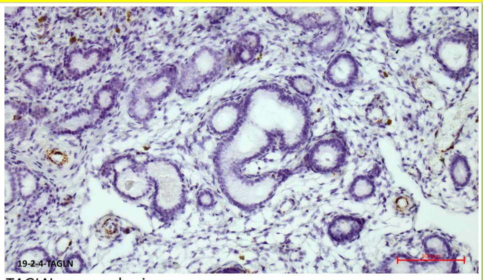<br>*TAGLN*, none or basic	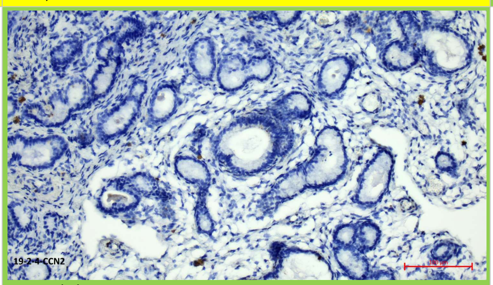<br>*CCN2*, slight	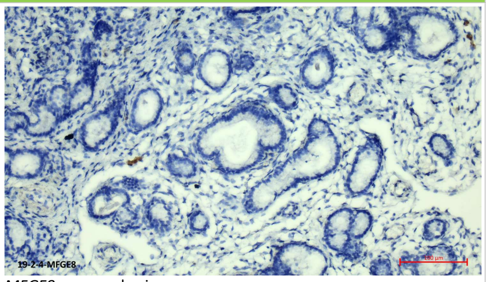<br>*MFGE8*, none or basic	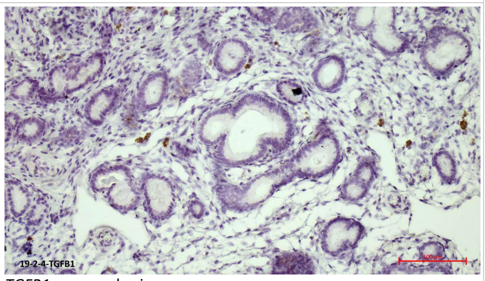<br>*TGFB1*, none or basic
19-2-5	none	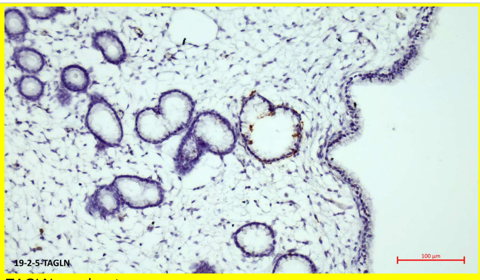<br>*TAGLN*, moderate	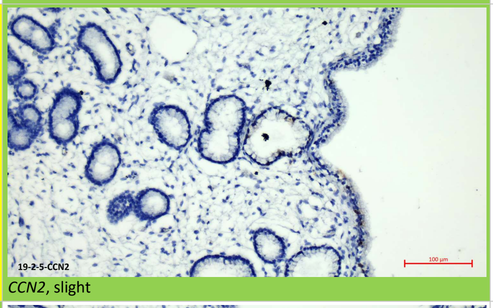<br>*CCN2*, slight	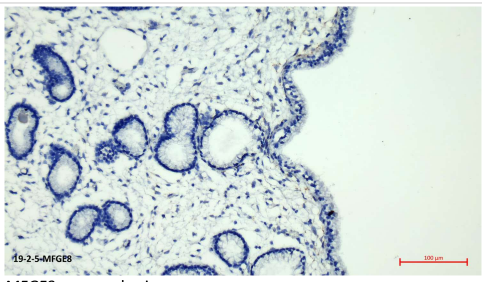<br>*MFGE8*, none or basic	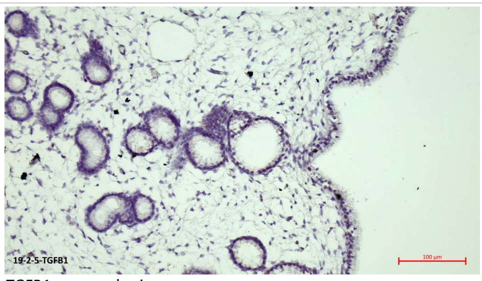<br>*TGFB1*, none or basic
19-2-6	none	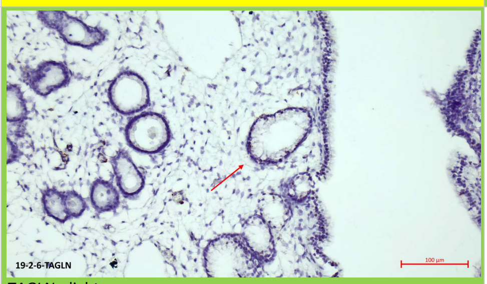<br>*TAGLN*, slight	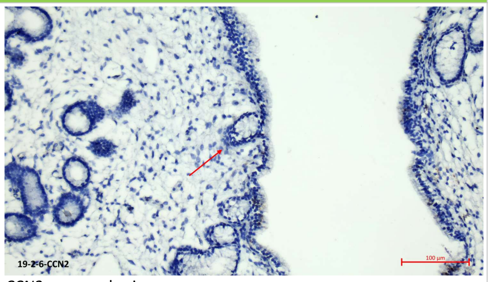<br>*CCN2*, none or basic	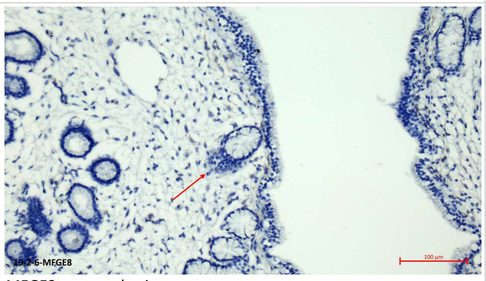<br>*MFGE8*, none or basic	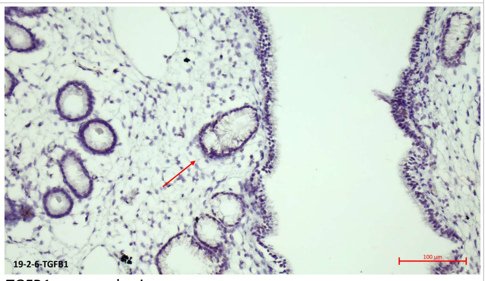<br>*TGFB1*, none or basic
19-2-7	1 to 3	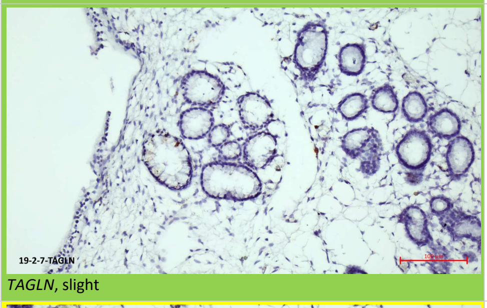<br>*TAGLN*, slight	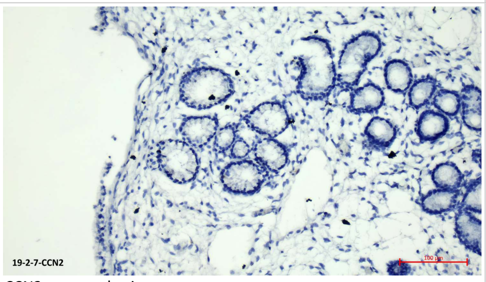<br>*CCN2*, none or basic	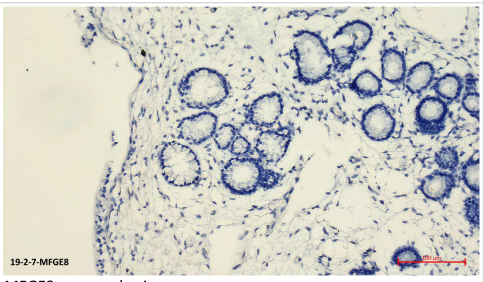<br>*MFGE8*, none or basic	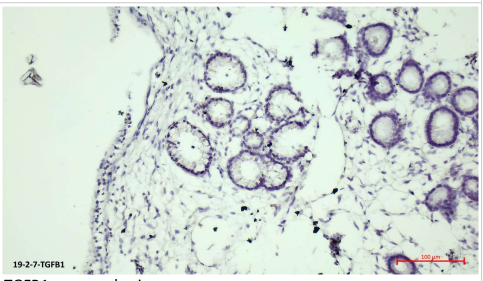<br>*TGFB1*, none or basic
19-2-8	none	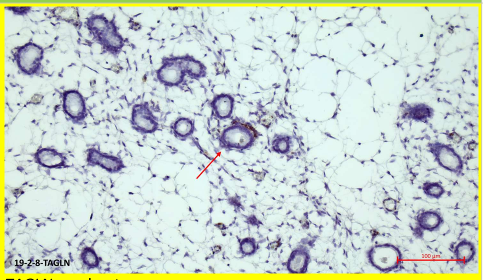<br>*TAGLN*, moderate	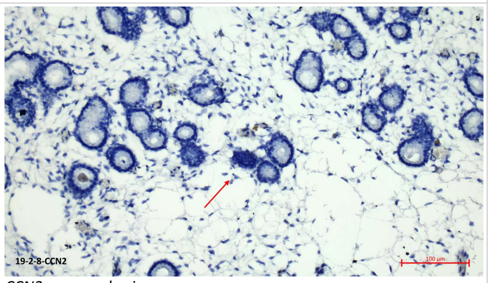<br>*CCN2*, none or basic	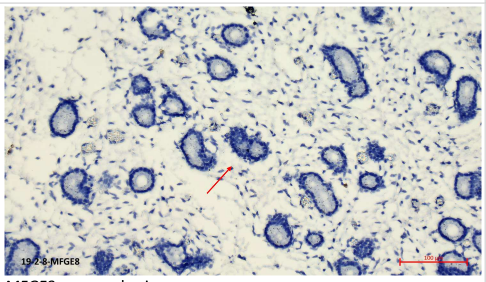<br>*MFGE8*, none or basic	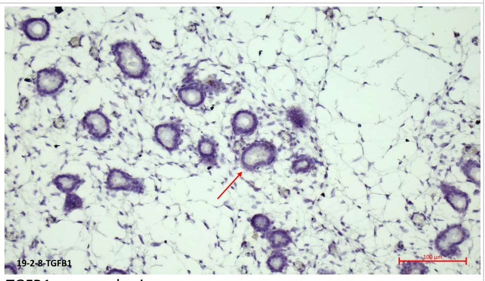<br>*TGFB1*, none or basic
9-2-11	none	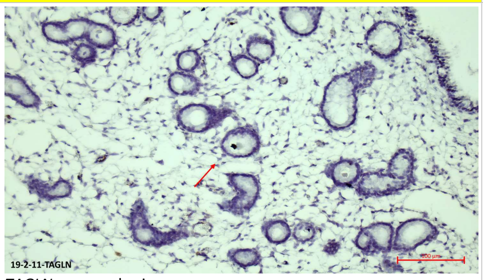<br>*TAGLN*, none or basic	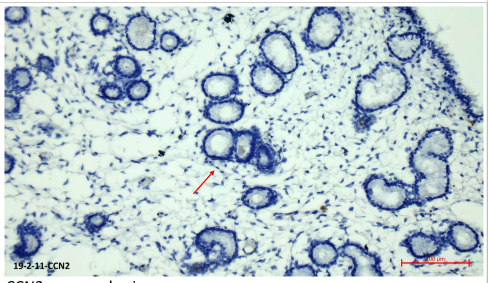<br>*CCN2*, none or basic	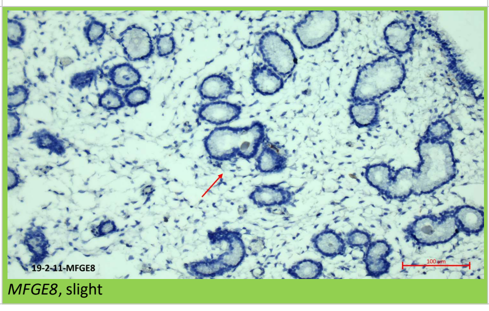<br>*MFGE8*, slight	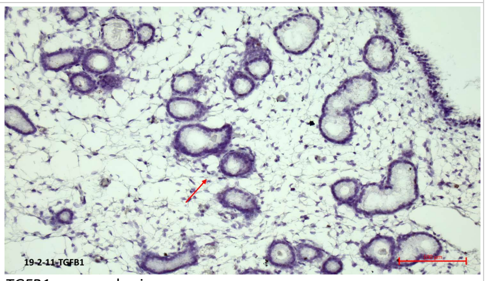<br>*TGFB1*, none or basic

Mare 11-1

	Concentric layers	*TAGLN*	*CCN2*	*MFGE8*
11-1-1	4 to 10	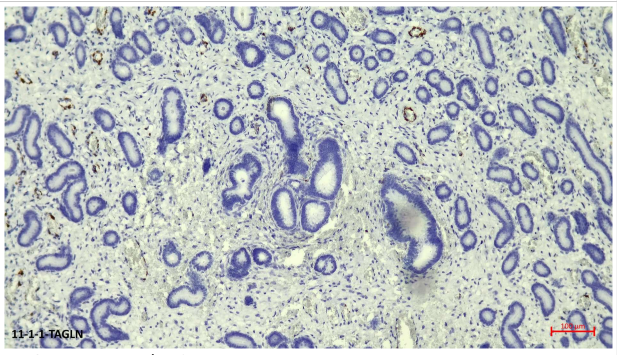 *TAGLN*, none or basic	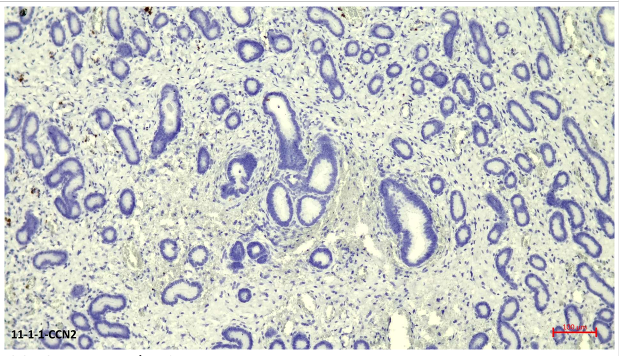 *CCN2*, none or basic	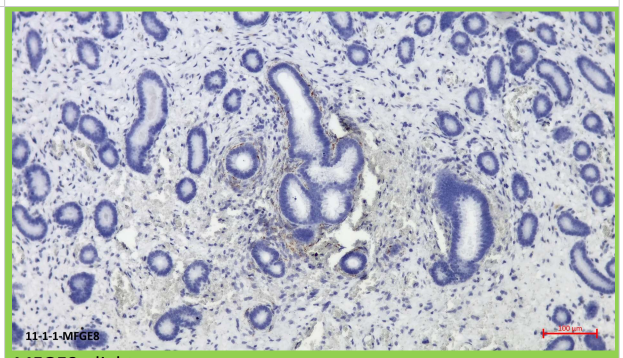 *MFGE8*, slight
11-1-2	4 to 10	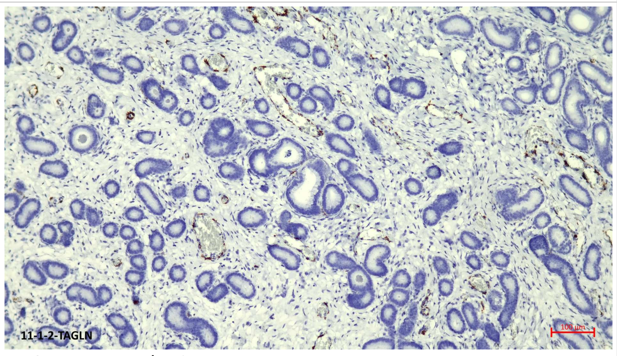 *TAGLN*, none or basic	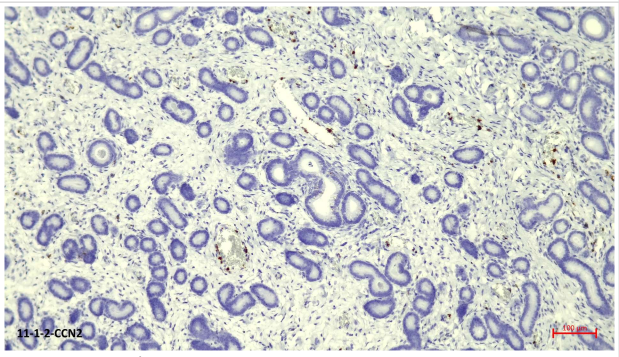 *CCN2*, none or basic	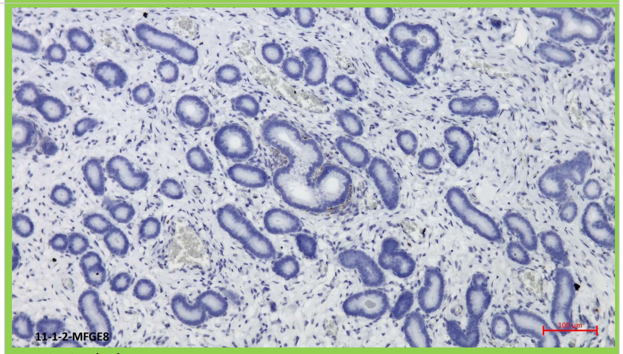 *MFGE8*, slight
11-1-3	4 to 10	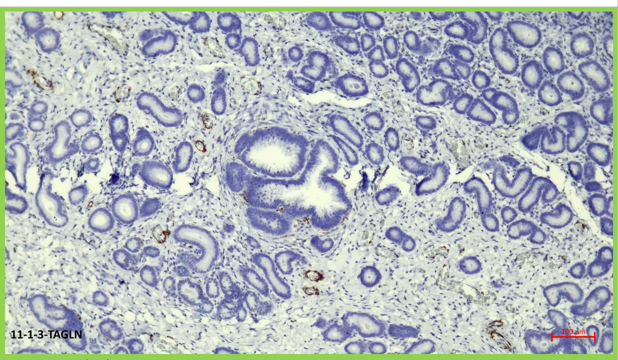 *TAGLN*, slight	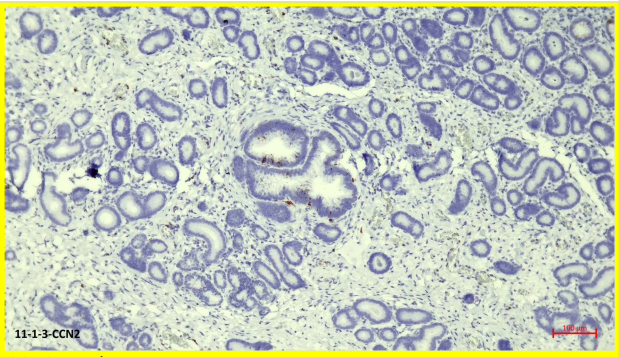 *CCN2*, moderate	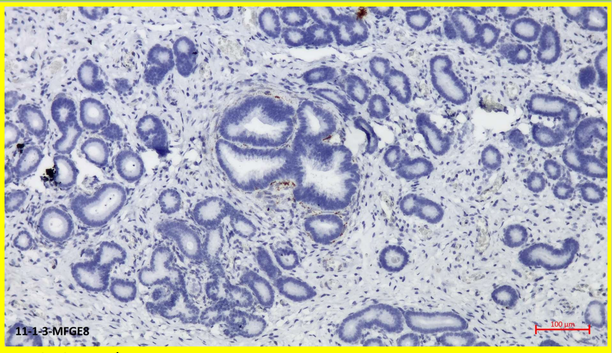 *MFGE8*, moderate
11-1-4	1 to 3	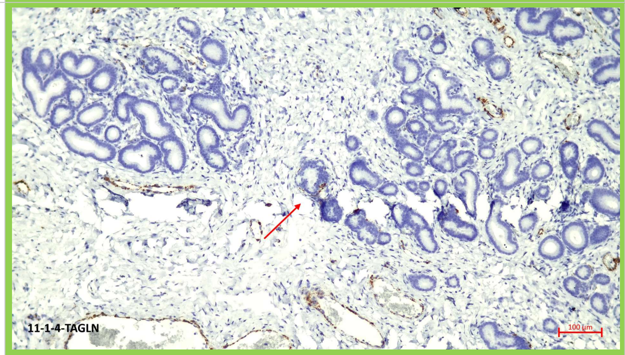 *TAGLN*, slight	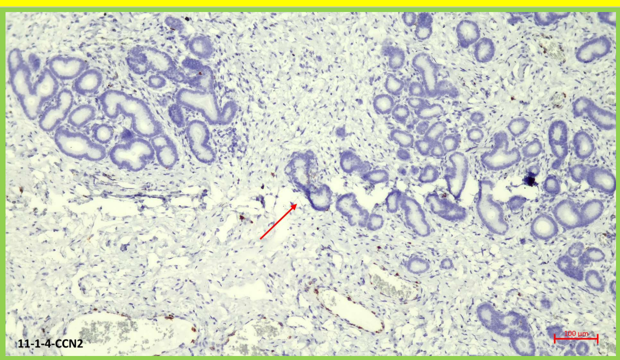 *CCN2*, slight	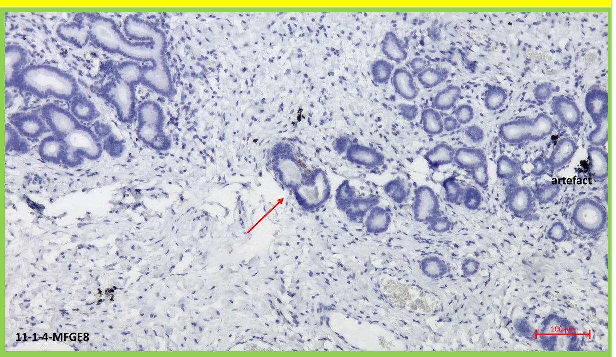 *MFGE8*, slight
11-1-5	4 to 10	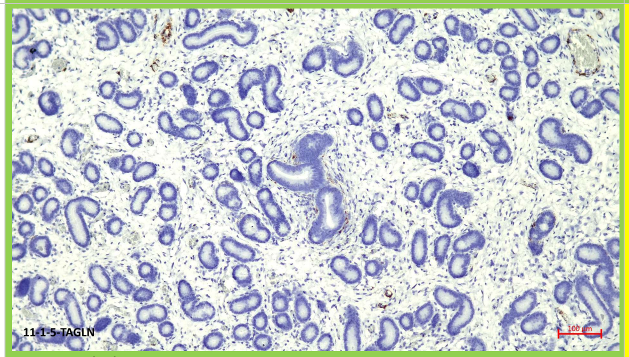 *TAGLN*, slight	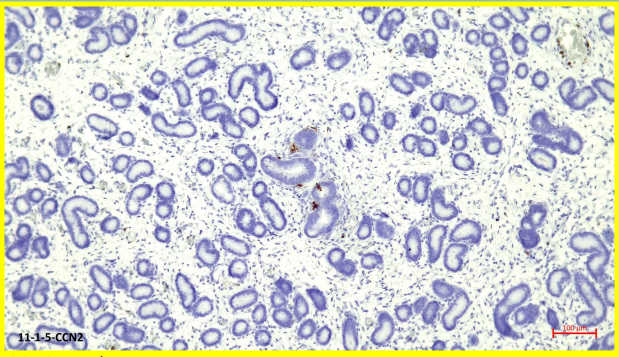 *CCN2*, moderate	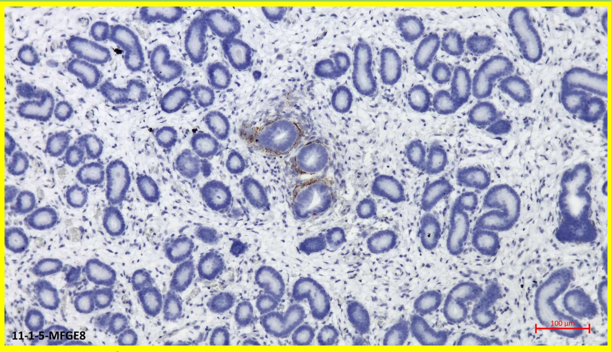 *MFGE8*, moderate
11-1-6	4 to 10	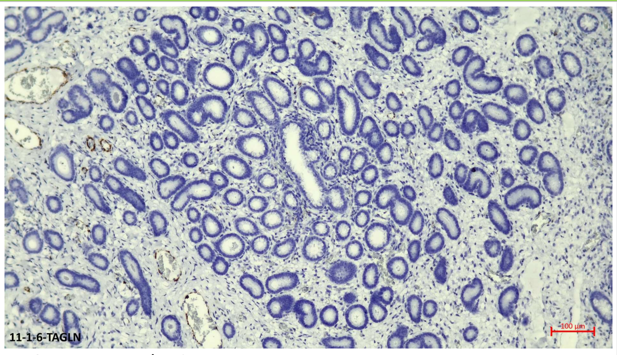 *TAGLN*, none or basic	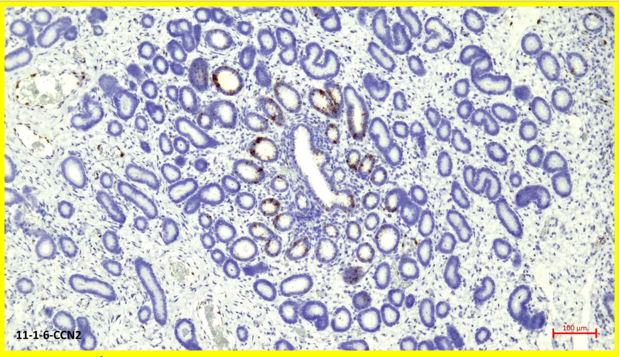 *CCN2*, moderate	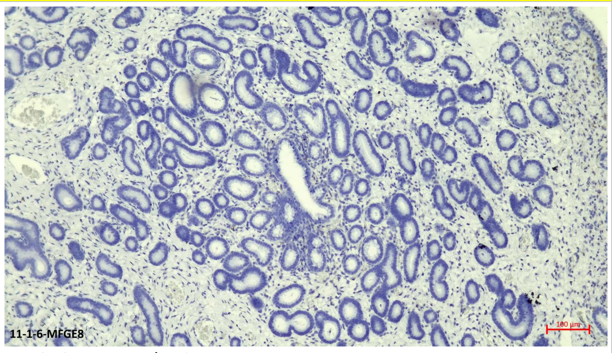 *MFGE8*, none or basic
11-1-7	4 to 10	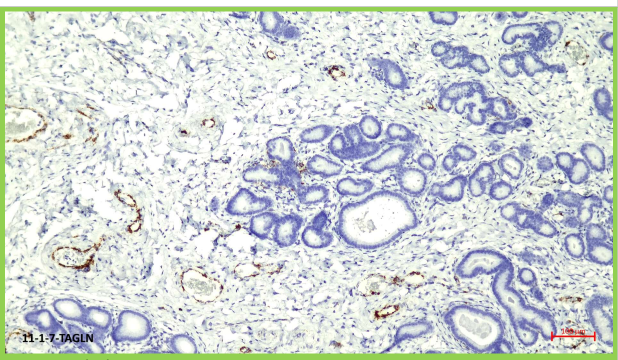 *TAGLN*, slight	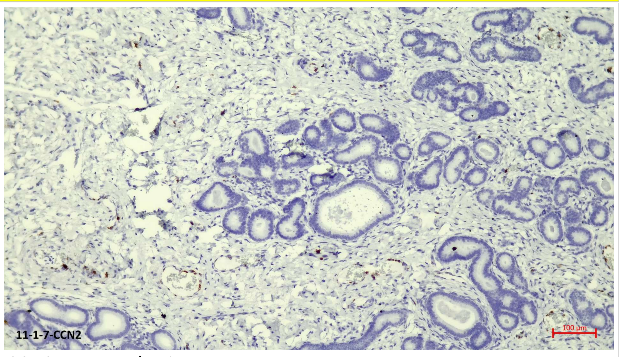 *CCN2*, none or basic	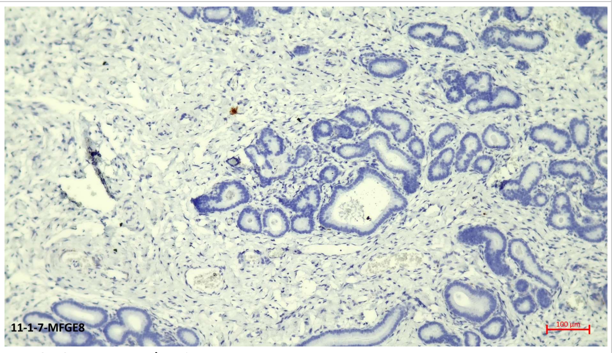 *MFGE8*, none or basic

<table>
<tr><td>11-1-8</td><td>4 to 10</td><td>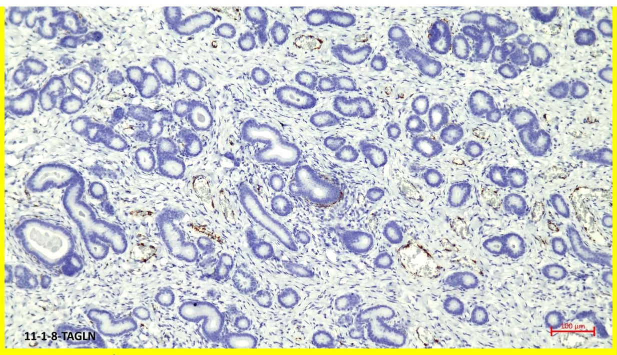
TAGLN, moderate</td><td>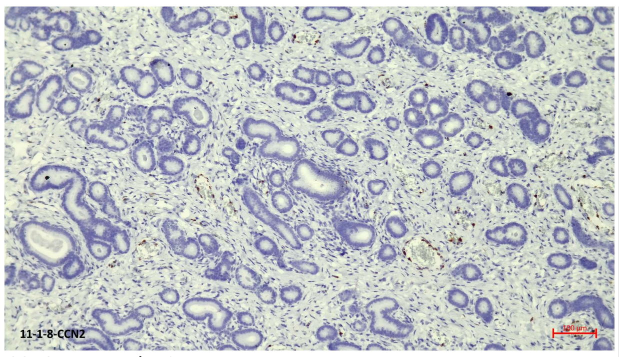
CCN2, none or basic</td><td>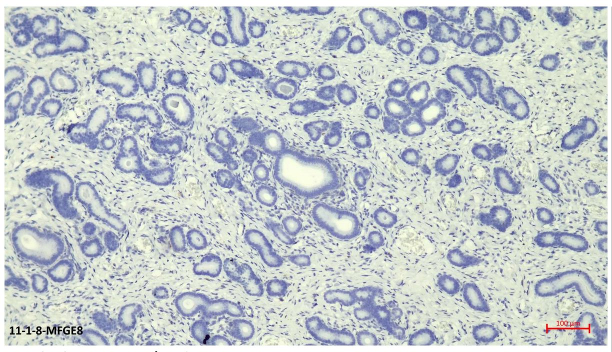
MFGE8, none or basic</td></tr>
<tr><td>11-1-9</td><td>none</td><td>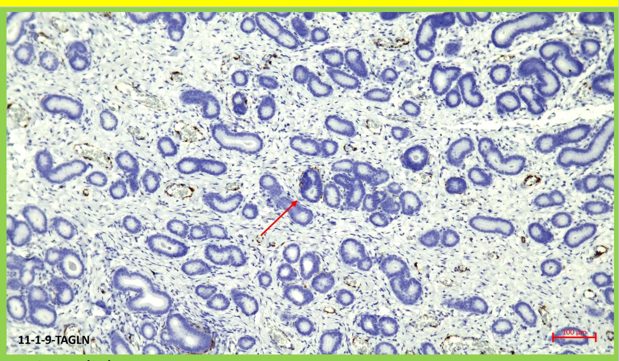
TAGLN, slight</td><td>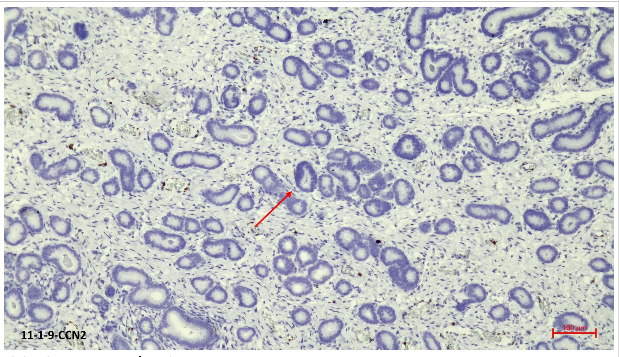
CCN2, none or basic</td><td>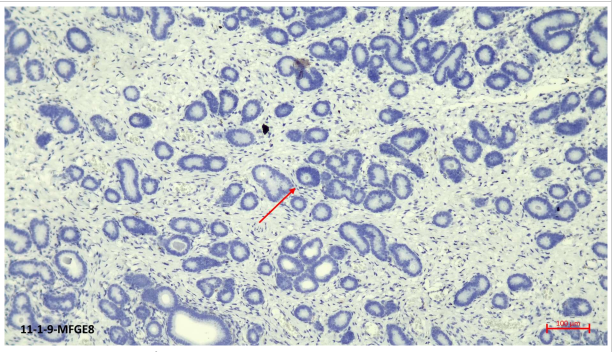
MFGE8, none or ba1sic</td></tr>
<tr><td>11-1-10</td><td>none</td><td>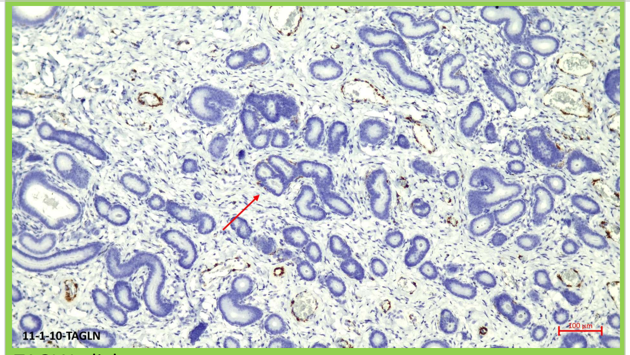
TAGLN, slight</td><td>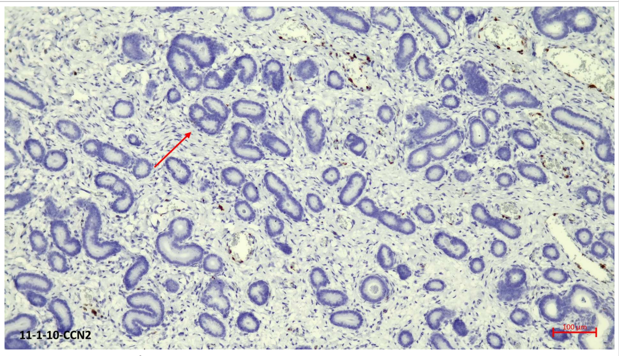
CCN2, none or basic</td><td>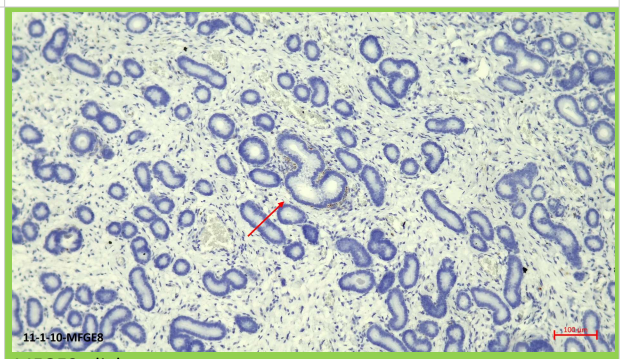
MFGE8, slight</td></tr>
<tr><td></td><td></td><td>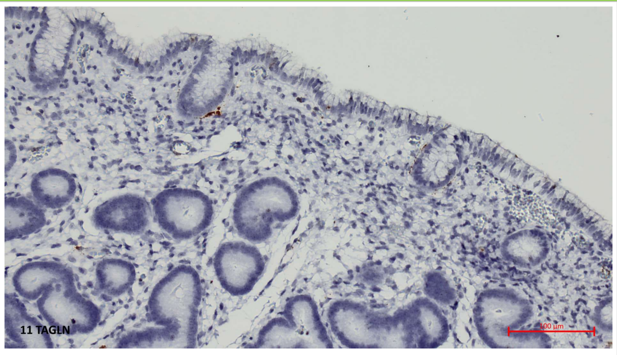</td><td>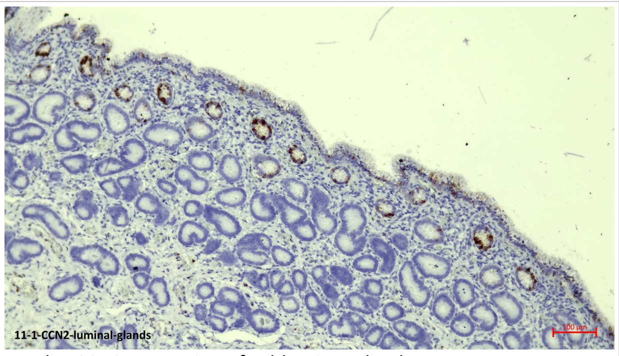
regular *CCN2* expression of subluminar glands</td><td>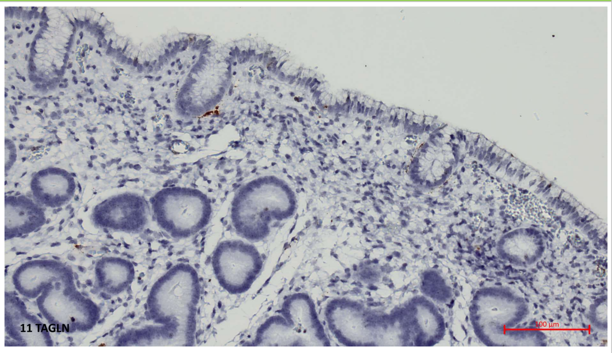</td></tr>
</table>

Mare 22-1

	Concentric layers	*TAGLN*	*CCN2*	*MFGE8*	*TGFB1*
22-1-1	1 to 3	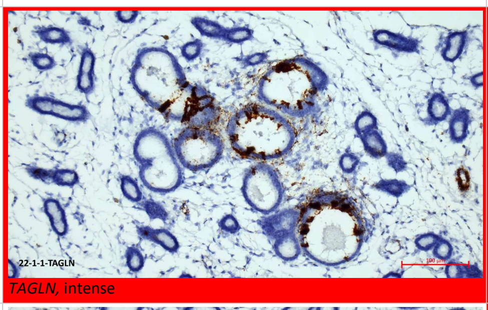 *TAGLN*, intense	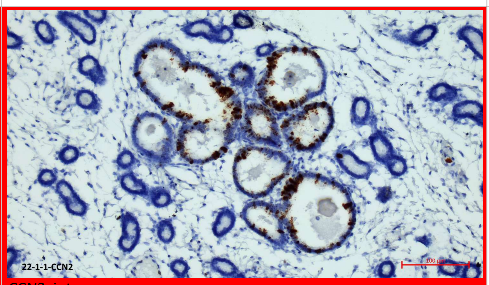 *CCN2*, intense	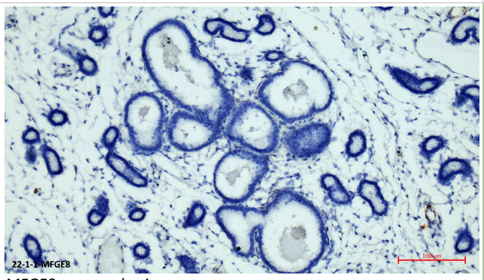 *MFGE8*, none or basic	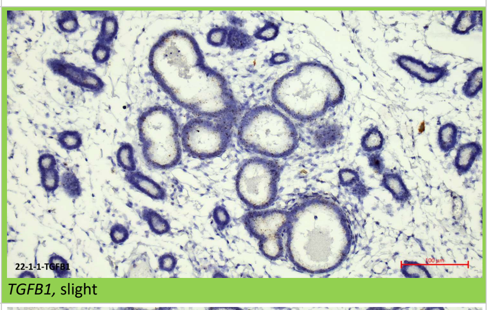 *TGFB1*, slight
22-1-2	1 to 3	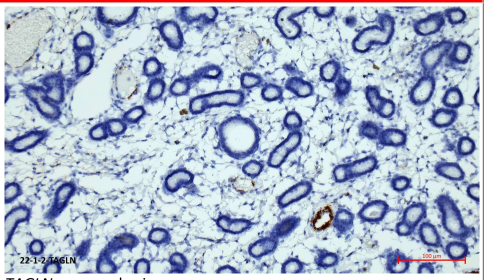 *TAGLN*, none or basic	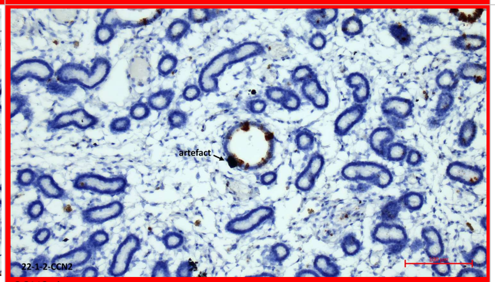 *CCN2*, intense	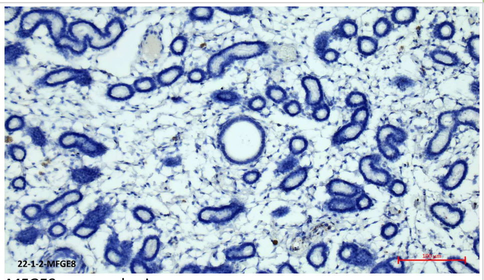 *MFGE8*, none or basic	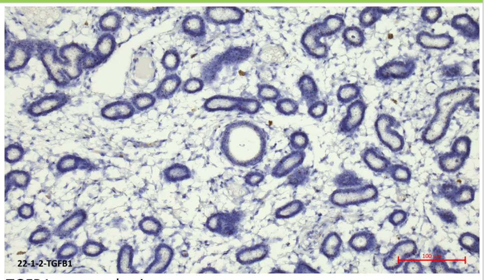 *TGFB1*, none or basic
22-1-4	none	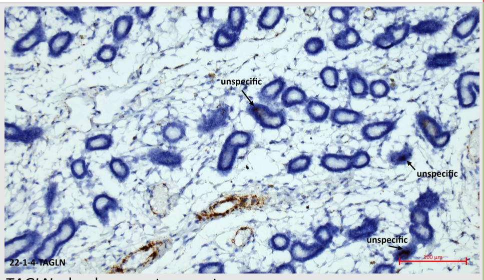 *TAGLN*, gland area not present.	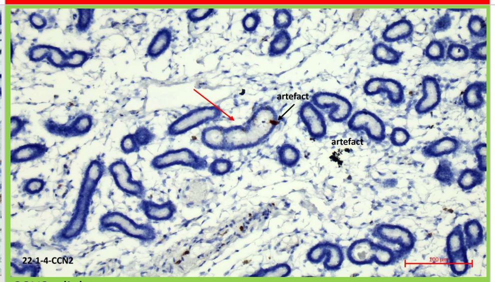 *CCN2*, slight	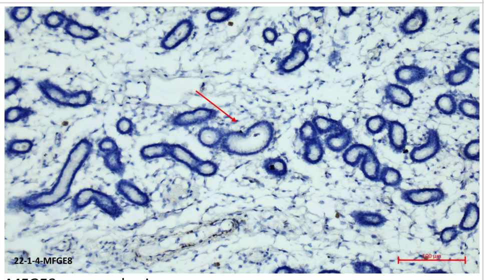 *MFGE8*, none or basic	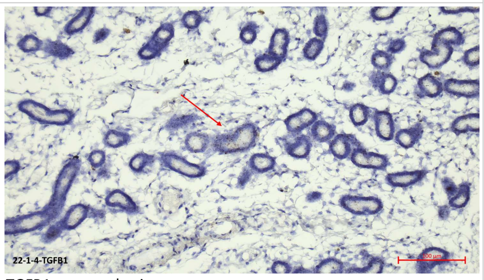 *TGFB1*, none or basic
22-1-6	1 to 3	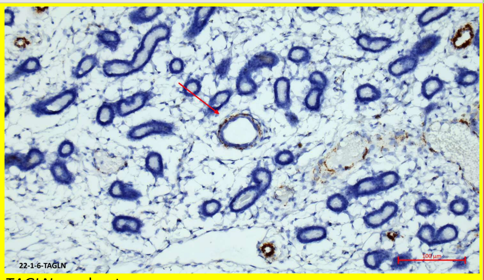 *TAGLN*, moderate	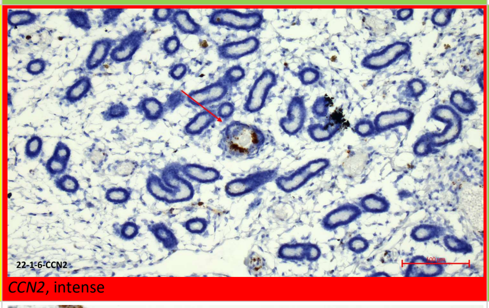 *CCN2*, intense	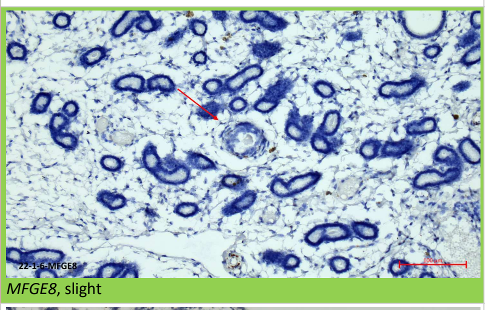 *MFGE8*, slight	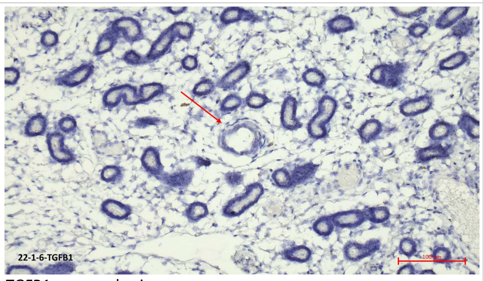 *TGFB1*, none or basic
		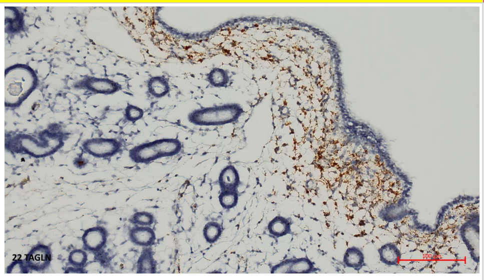	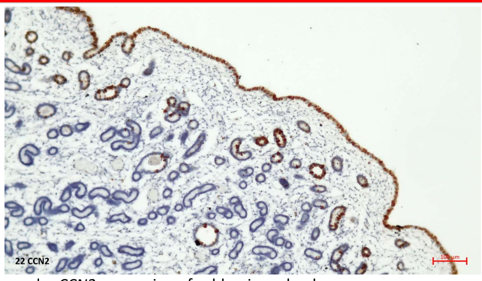 regular *CCN2* expression of subluminar glands	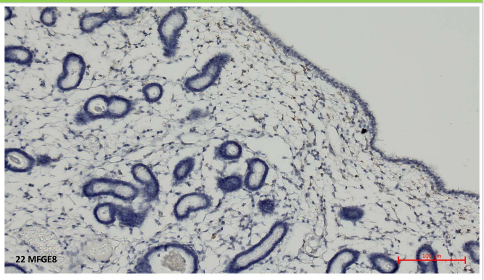	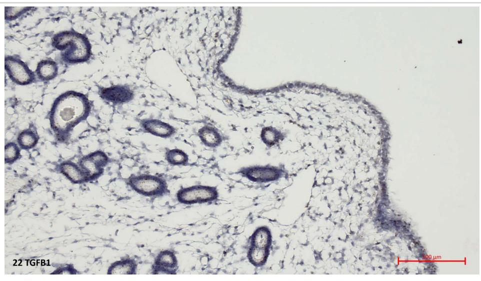

Vessels and subluminal Areas

		TAGLN	*CCN2*	*MFGE8*	*TGFB1*
Mare 1	Vessels	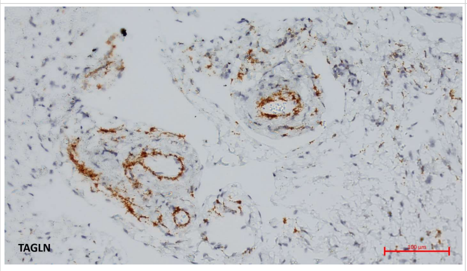	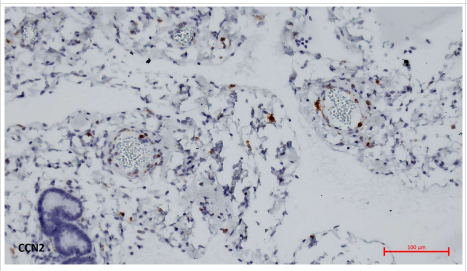	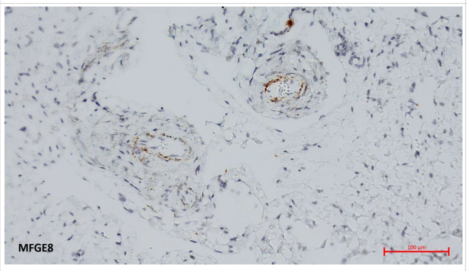	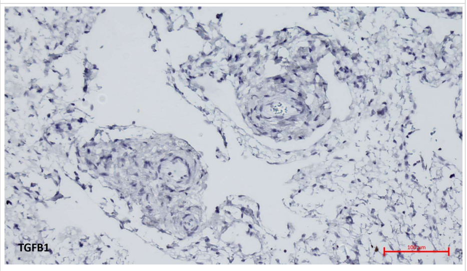
Mare 24	Vessels	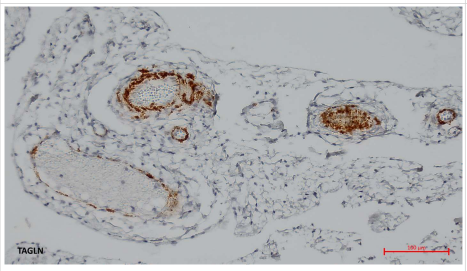	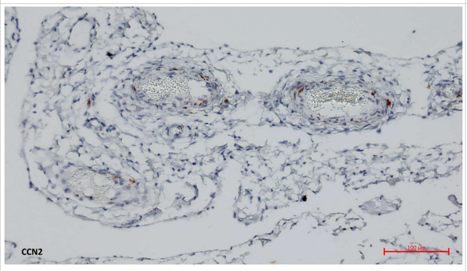	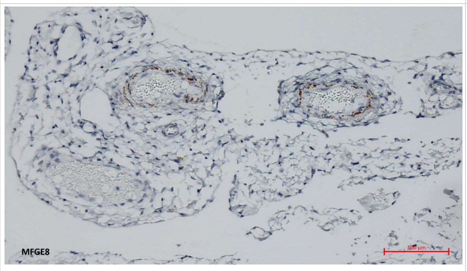	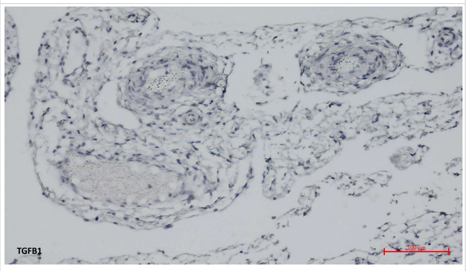
Mare 22	Vessels	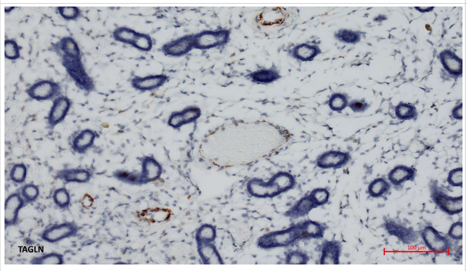	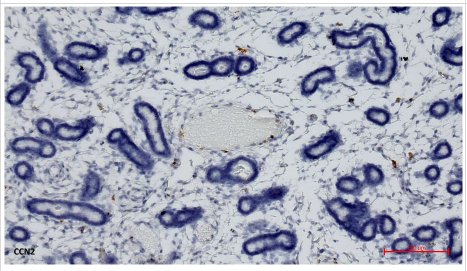	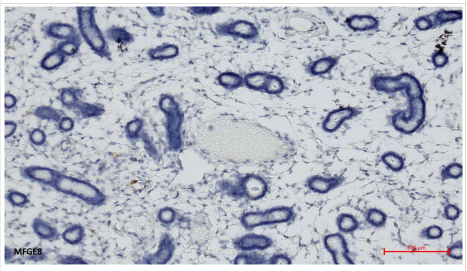	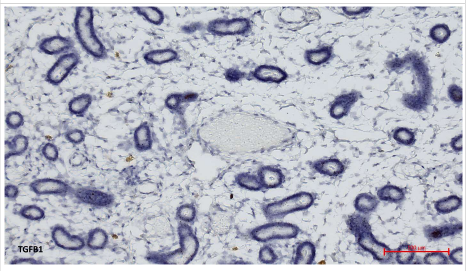
Mare 24	Vessels	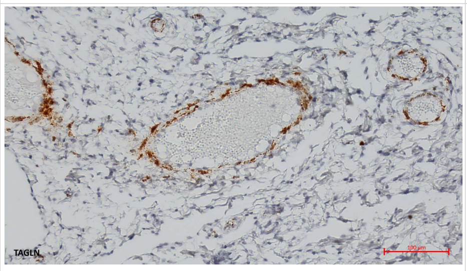	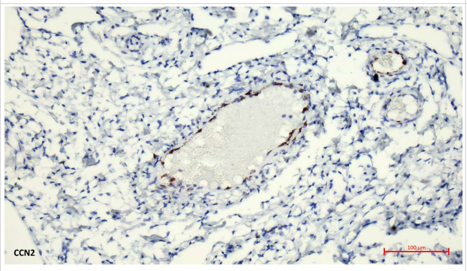	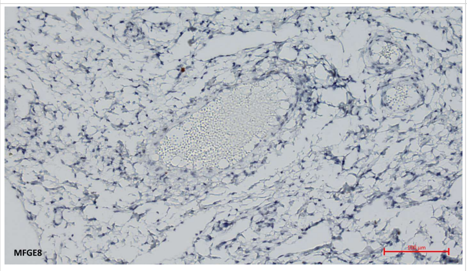	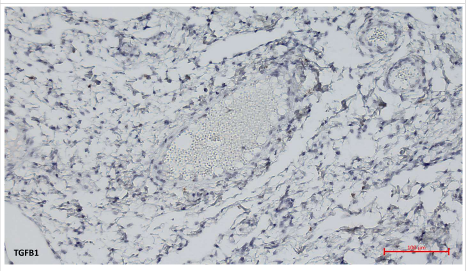
Mare 1	Luminal epithelium and subluminar area.	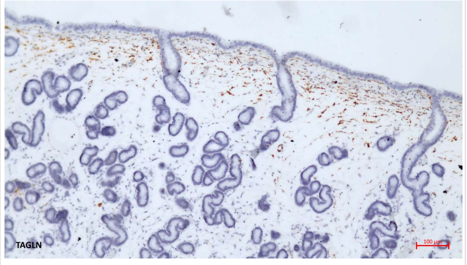	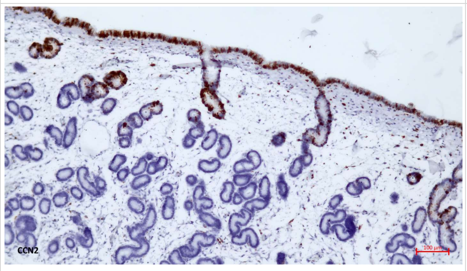	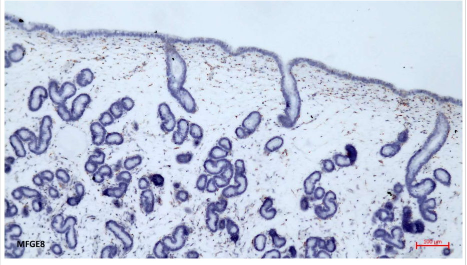	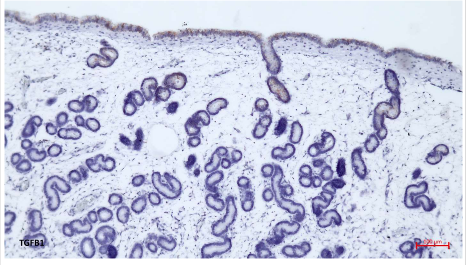
Mare 24	Luminal epithelium and subluminar area.	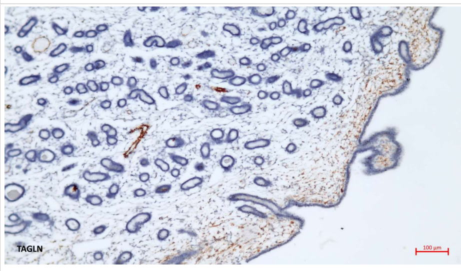	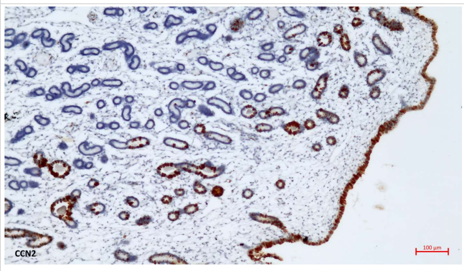	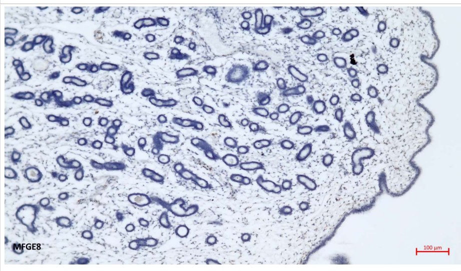	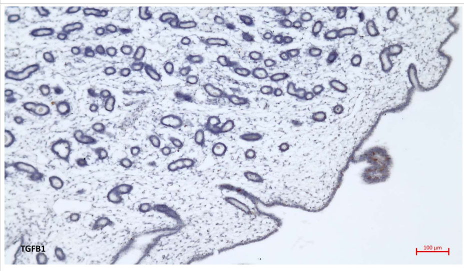
Mare 23	Luminal epithelium and subluminar area.	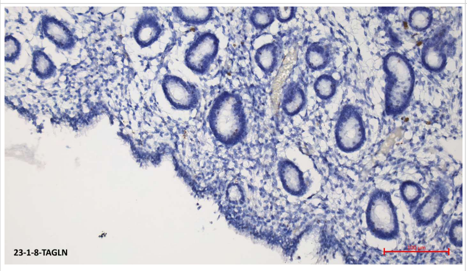	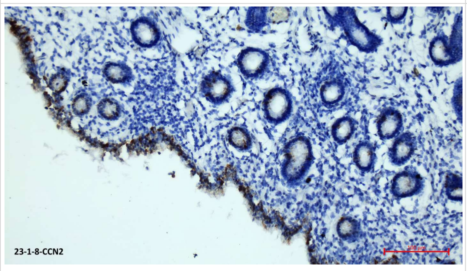	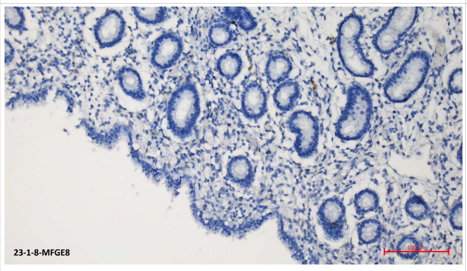	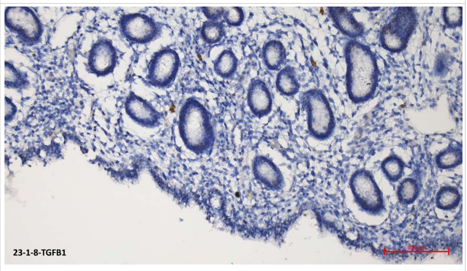
Mare 19	Luminal epithelium and subluminar area.	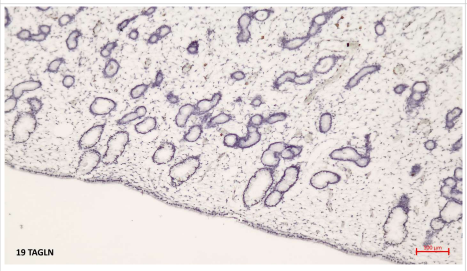	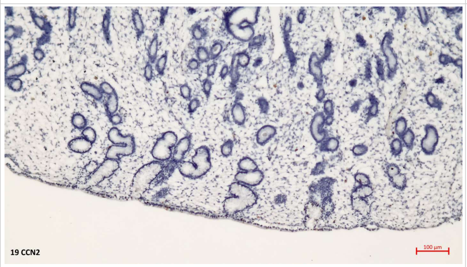	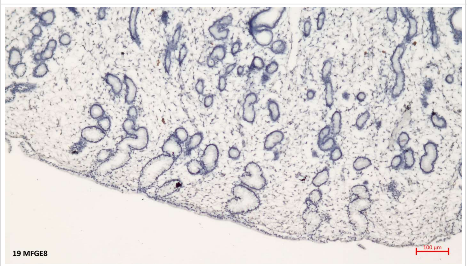	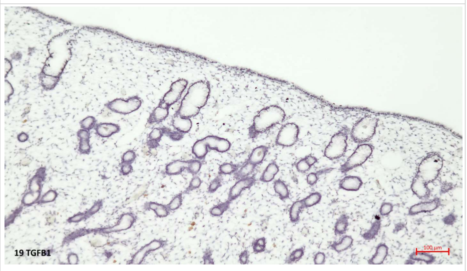

Examples of Fibrotic Glandular Areas without Overexpression of *TAGLN*, *CCN2*, *MFGE8*, or *TGFB1*

		TAGLN	*CCN2*	*MFGE8*	*TGFB1*
19-2-12	1 to 3	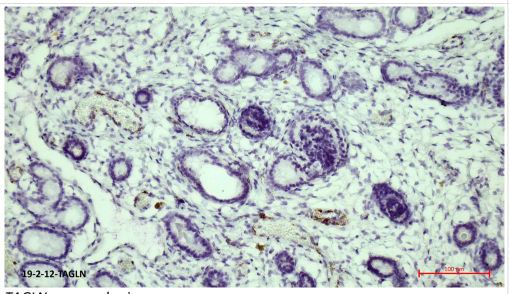<br>*TAGLN*, none or basic	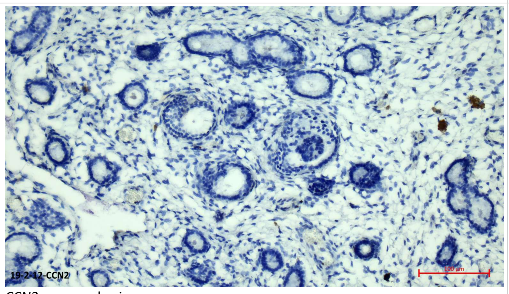<br>*CCN2*, none or basic	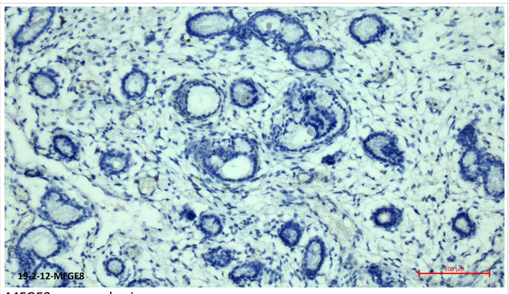<br>*MFGE8*, none or basic	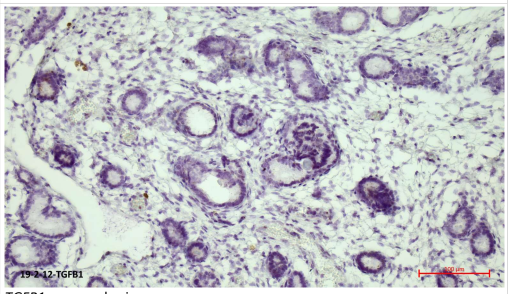<br>*TGFB1*, none or basic
23-1-6	1 to 3	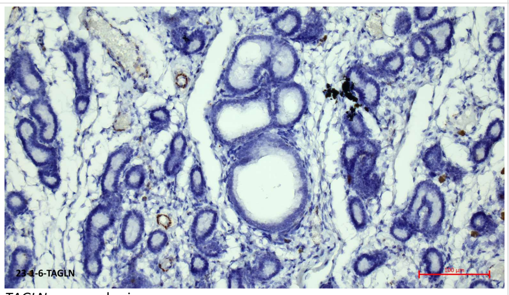<br>*TAGLN*, none or basic	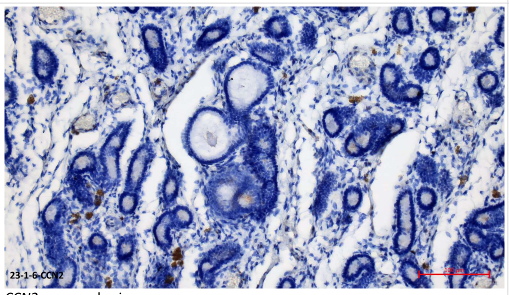<br>*CCN2*, none or basic	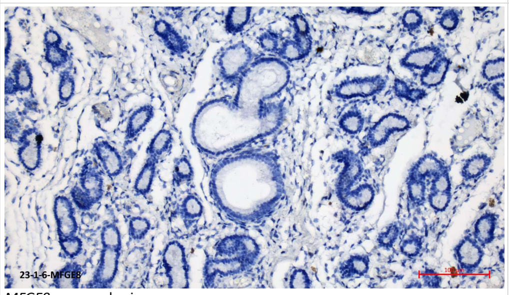<br>*MFGE8*, none or basic	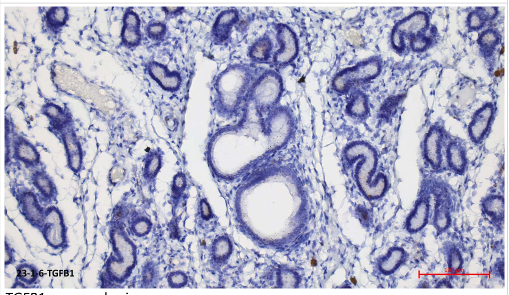<br>*TGFB1*, none or basic
24-1-5	4 to 10	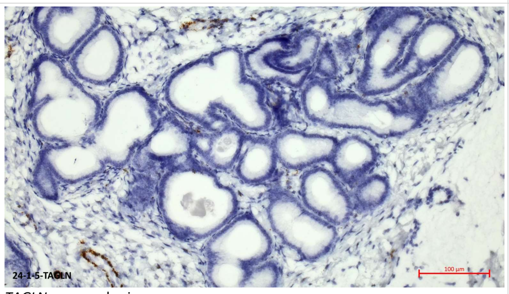<br>*TAGLN*, none or basic	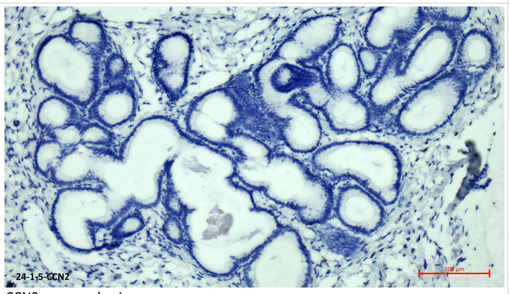<br>*CCN2*, none or basic	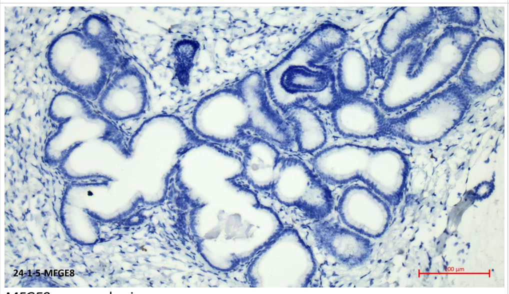<br>*MFGE8*, none or basic	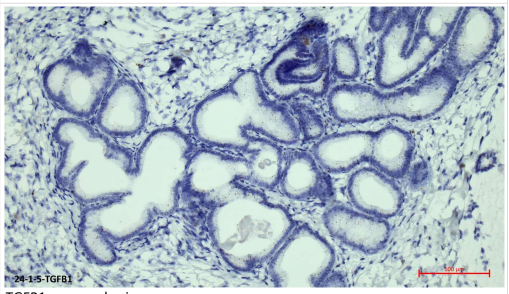<br>*TGFB1*, none or basic
24-1-7	1 to 3	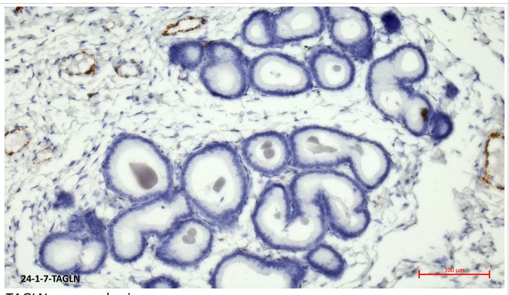<br>*TAGLN*, none or basic	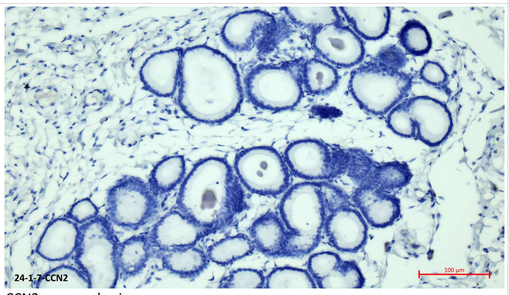<br>*CCN2*, none or basic	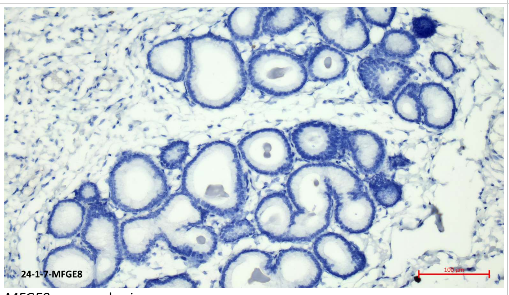<br>*MFGE8*, none or basic	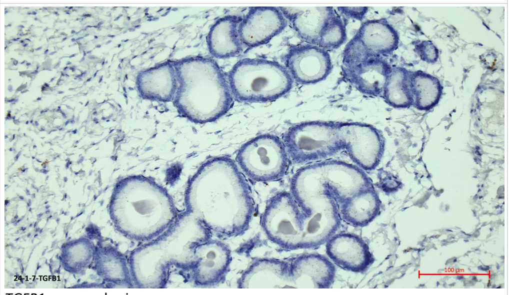<br>*TGFB1*, none or basic
24-1-9	1 to 3	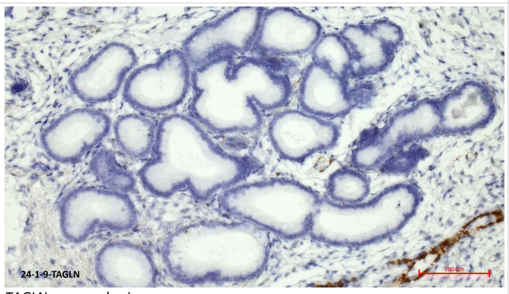<br>*TAGLN*, none or basic	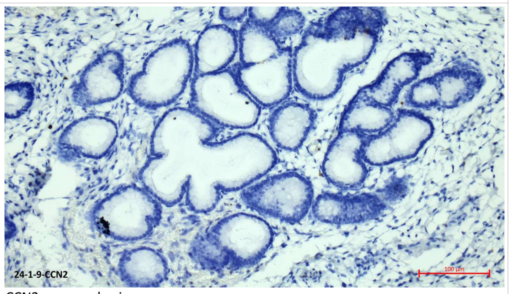<br>*CCN2*, none or basic	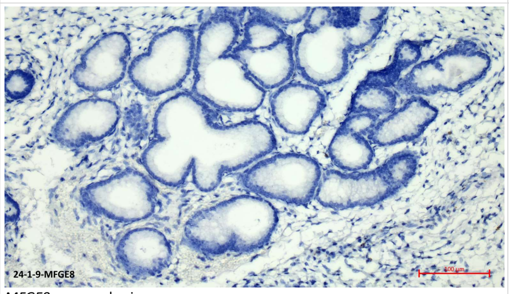<br>*MFGE8*, none or basic	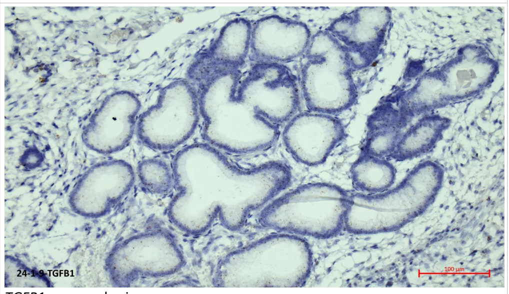<br>*TGFB1*, none or basic

Supplementary Figure M2 UMAP of integrated data with a resolution of 0.5, split by treatment and mare (n = 4).

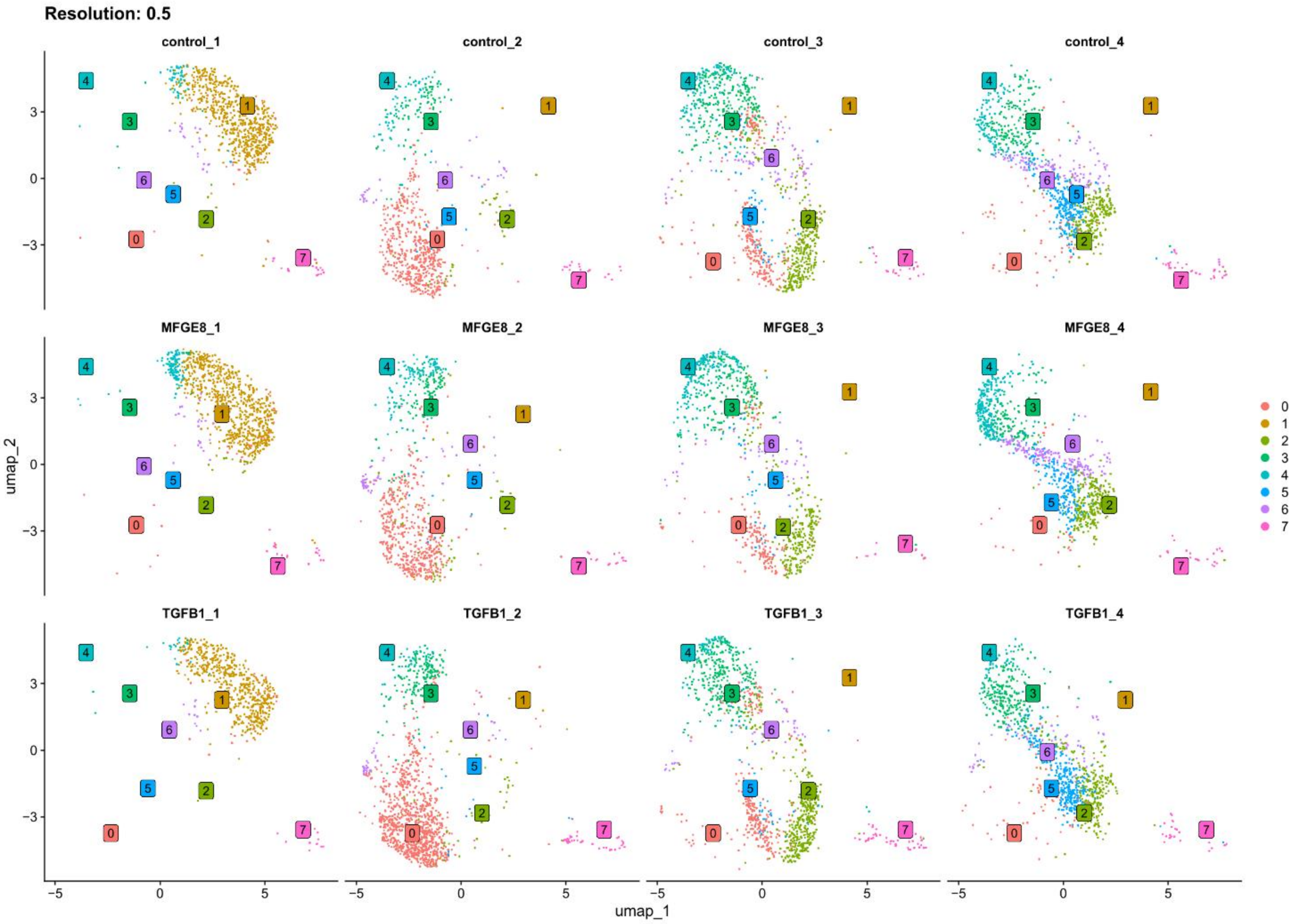

Supplementary Figure M3 PTEN pathway from IPA - Treatments Cluster 4

PTEN Signaling : control_vs_MFGE8 : Expr Fold Change

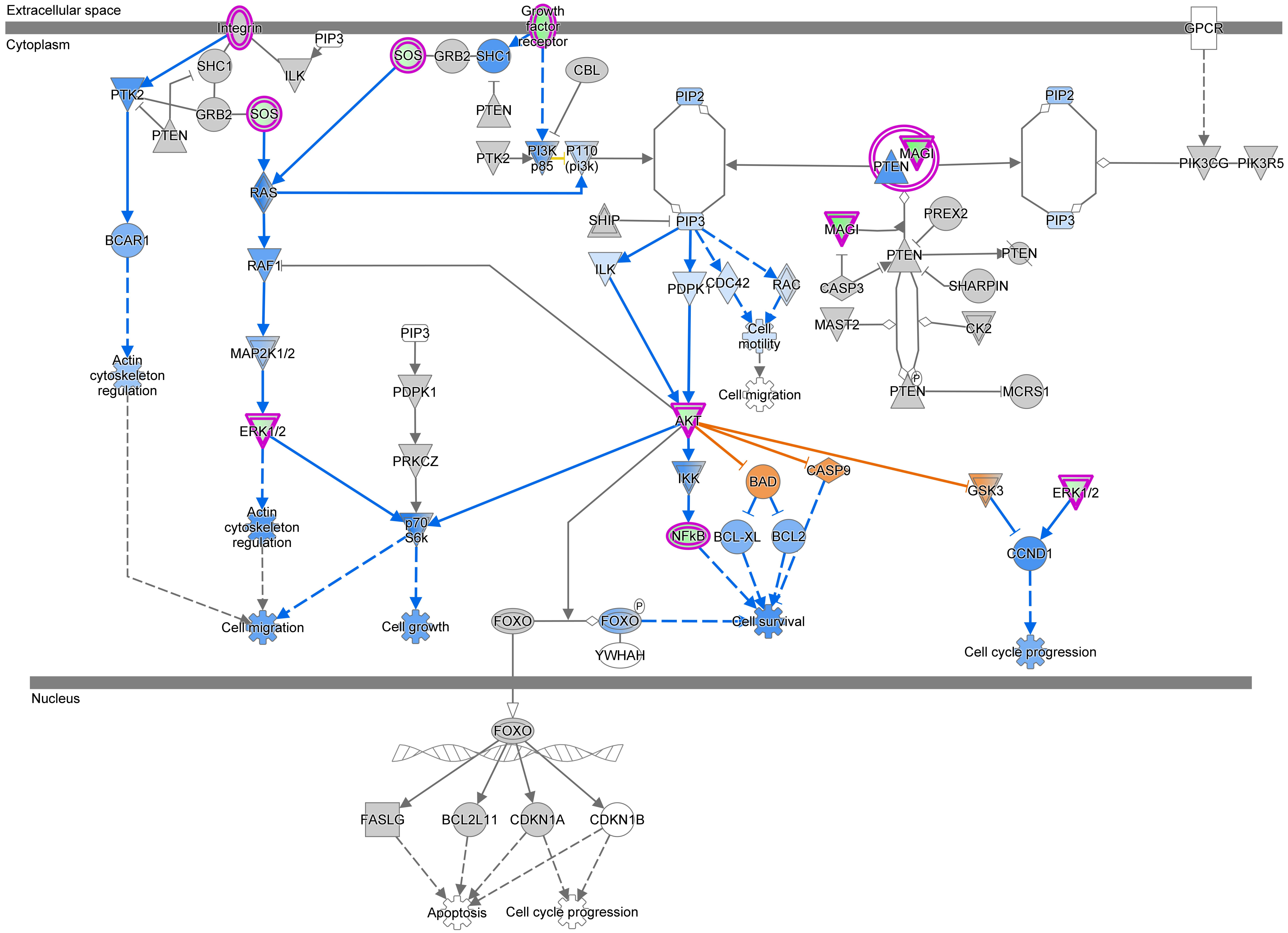

© 2000-2025 QIAGEN. All rights reserved.

PTEN Signaling : control_vs_TGFB1 : Expr Fold Change

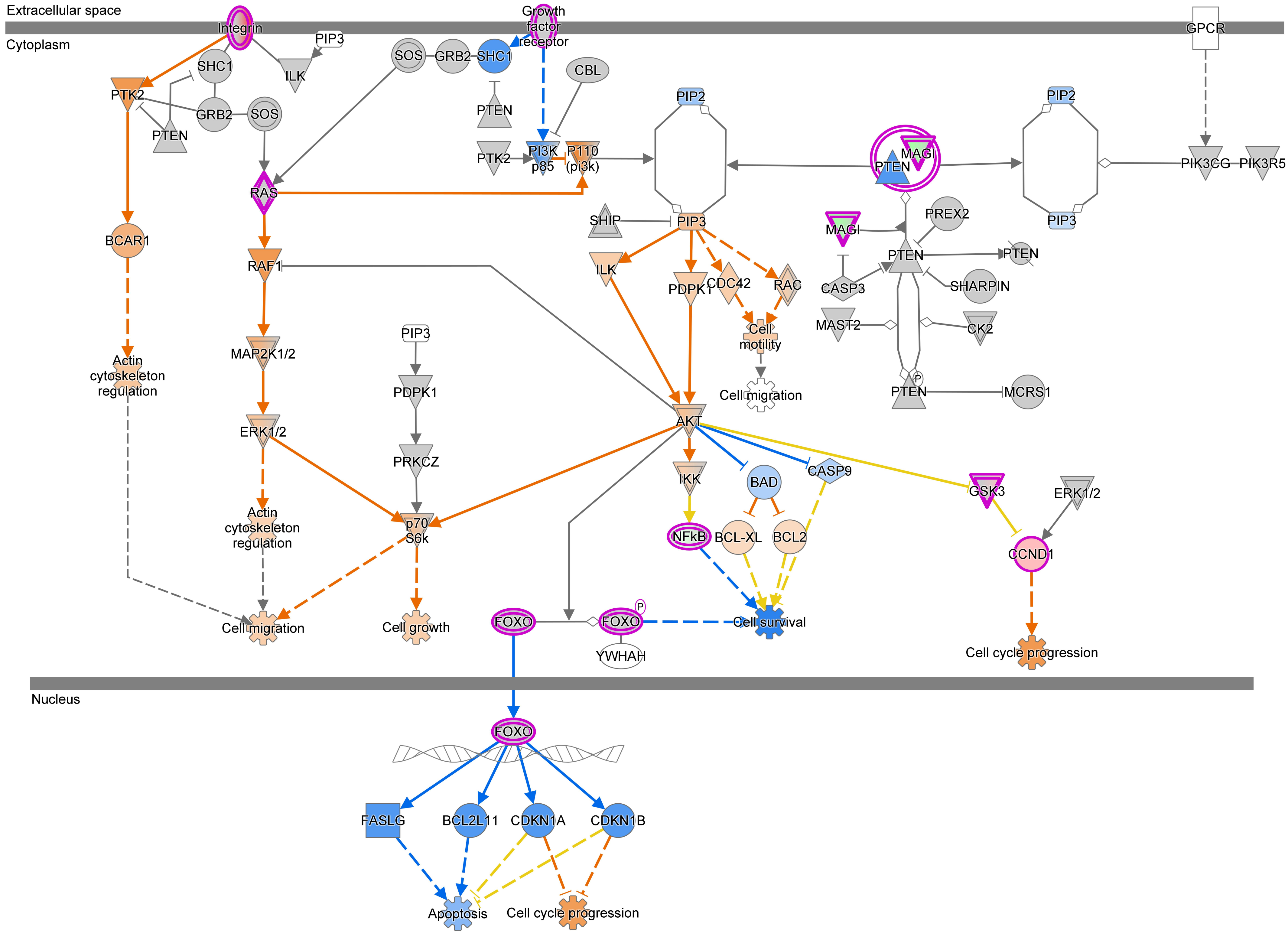

© 2000-2025 QIAGEN. All rights reserved.

IPA Legend

Übersicht Describes the node shapes, tool bar icons, colors, and more in IPA

Answer

IPA Legend

This legend provides a key to the main features of Network Explorer, My Pathways, and Canonical Pathways, including molecule shapes and colors as well as relationship labels and types.

Node Shapes

Network Shapes

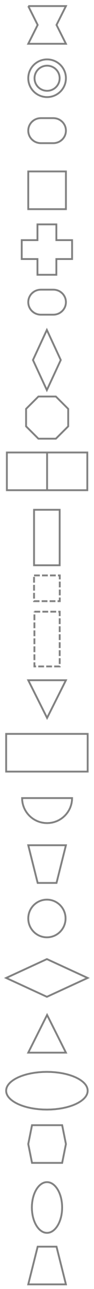

- Canonical Pathway
- Complex/Group
- Chemical/Toxicant
- Cytokine
- Disease
- Drug
- Enzyme
- Function
- Fusion gene/product
- G-Protein Coupled Receptor
- Growth Factor
- Ion Channel
- Kinase
- Ligand-dependent Nuclear Receptor
- Mature microRNA
- microRNA
- Other
- Peptidase
- Phosphatase
- Transcriptional Regulator
- Translational Regulator
- Transmembrane Receptor
- Transporter

Path Designer Shapes

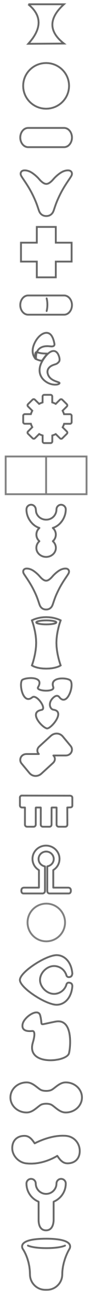

- Canonical Pathway
- Complex/Group
- Chemical/Toxicant
- Cytokine
- Disease
- Drug
- Enzyme
- Function
- Fusion gene/product
- G-Protein Coupled Receptor
- Growth Factor
- Ion Channel
- Kinase
- Ligand-dependent Nuclear Receptor
- Mature microRNA
- microRNA
- Other
- Peptidase
- Phosphatase
- Transcriptional Regulator
- Translational Regulator
- Transmembrane Receptor
- Transporter

A high-resolution version of this image can be found here: Shape legend

Other molecule symbols:

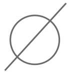
Degraded protein (shown only in Canonical Pathways)

Relationship Types and Labels

**A**	Activation
**B**	Binding
**C**	Causation/Leads to
**CO**	Correlation
**CC**	Chemical-Chemical interaction
**CP**	Chemical-Protein interaction
**E**	Expression (includes metabolism/ synthesis for chemicals)
**EC**	Enzyme Catalysis
**I**	Inhibition
**L**	Molecular Cleavage (includes degradation for Chemicals)
**LO**	Localization
**M**	Biochemical Modification
**miT**	microRNA Targeting
**MB**	Group/complex Membership
**nTRR**	Non-Targeting RNA-RNA Interaction
**P**	Phosphorylation/Dephosphorylation
**PD**	Protein-DNA binding
**PP**	Protein-Protein binding
**PR**	Protein-RNA binding
**PY**	Processing Yields
**RB**	Regulation of Binding
**RE**	Reaction
**RR**	RNA-RNA Binding
**T**	Transcription
**TR**	Translocation
**UB**	Ubiquitination

Relationship line descriptions

Note: The line directionality may change when the Molecule Activity Predictor (MAP) tool is applied.

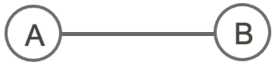

Chemical-chemical interactions, chemical-protein interactions, correlation, protein-protein interactions, RNA-RNA interactions: non-targeting interactions

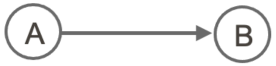

Activation, causation, expression, localization, membership, modification, molecular cleavage, phosphorylation, protein-DNA interactions, protein-RNA interactions, regulation of binding, transcription

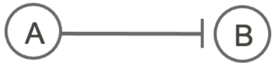

Inhibition, ubiquitination

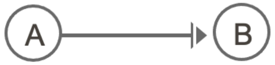

Inhibits and acts on

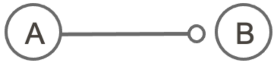

Leads to

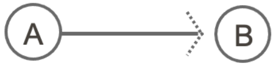

Processing yields

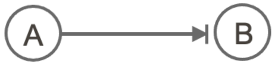

RNA-RNA interactions; microRNA interactions

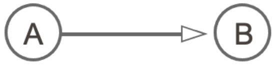

Translocation

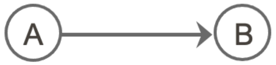

Reaction

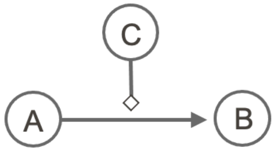

Enzyme catalysis

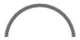

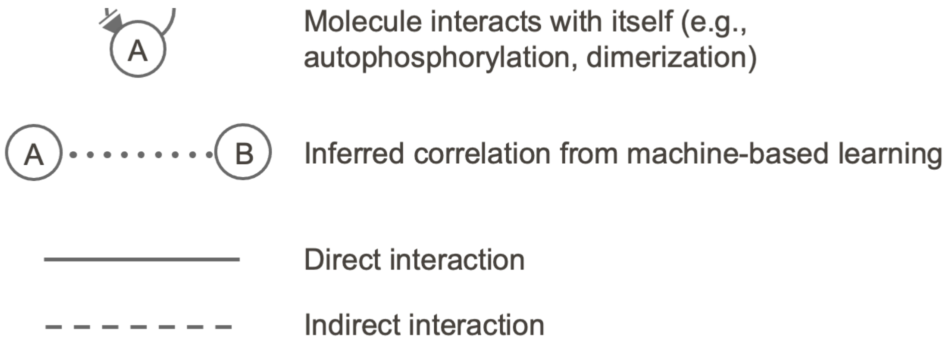

A high-resolution version of this image can be found here: Relationship legend

A relationship with an X over the line indicates that the interaction does not occur. These relationships are only used in disease pathways to indicate an interaction that would normally happen in the absence of the disease but does not happen in the disease context.

Ubiquitination edges are considered inhibitory towards a recipient protein's activity.

An arrow pointing from A to B signifies different actions for different circumstances, as described below:

For signaling pathways:

An arrow pointing from A to B signifies that A causes B to be activated (includes any direct interaction: e.g., binding, phosphorylation, dephosphorylation, etc).

For metabolic pathways:

An arrow pointing from A to B signifies that B is produced from A.

For ligands/receptors:

An arrow pointing from a ligand to a receptor signifies that the ligand binds the receptor and subsequently leads to activation of the receptor. This binding event does not necessarily directly activate the receptor; activation of the receptor could be caused by events secondary to the ligand/receptor binding event.

MAP Prediction Legend

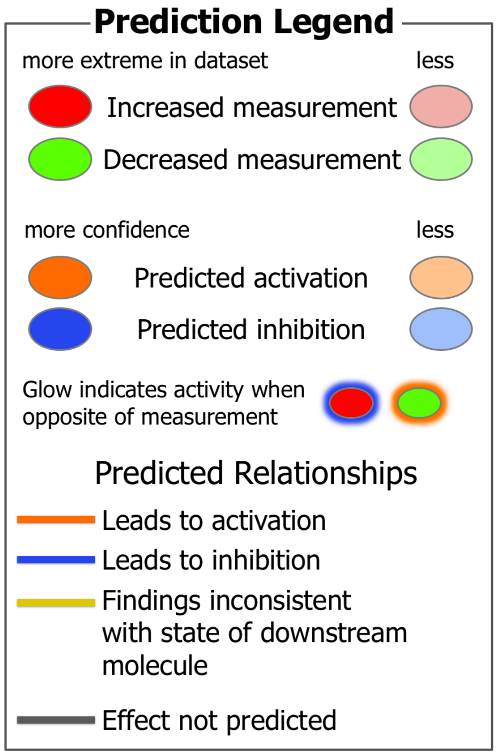

A high-resolution version of this image can be found here: High-resolution MAP legend

MAP Prediction Legend for Graphical Summary networks

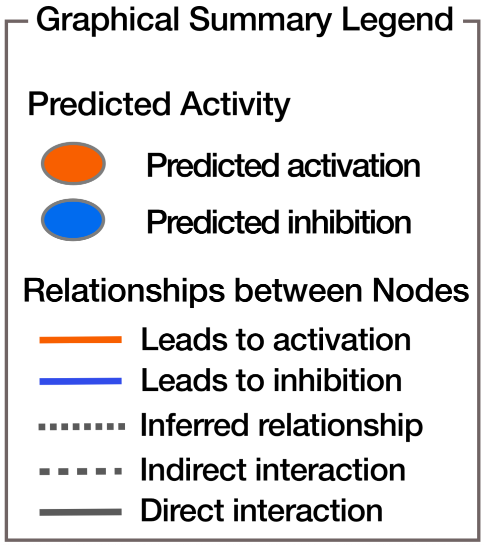

The MAP Prediction Legend for Graphical Summary networks reflects the same information as the MAP Prediction Legend but adds the machine-based learning relationship line (Inferred relationship) that is part of this feature.

A high-resolution version of this image can be found at this link: High-Resolution MAP Graphical Summary Legend

Interpretation of MAP relationship lines in networks/pathways

The coloring and type of relationship lines will often be modified when MAP predictions are applied, as outlined in the table below:

Upstream molecule's predicted activity	Downstream molecule's predicted activity	Consistency of Findings with state of downstream molecule	Relationship line coloring and type
Activated	Increased	Consistent	Orange activation
Inhibited	Decreased	Consistent	Blue activation
Activated	Decreased	Consistent	Blue inhibition
Inhibited	Increased	Consistent	Orange inhibition
Activated	Increased	Inconsistent	Yellow inhibition
Inhibited	Decreased	Inconsistent	Yellow inhibition
Activated	Decreased	Inconsistent	Yellow activation
Inhibited	Increased	Inconsistent	Yellow activation

Overlay tags

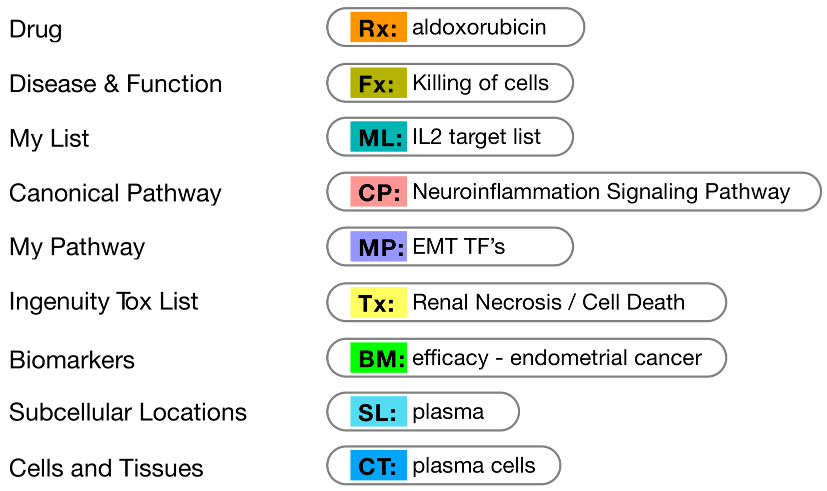

A high-resolution version of this image can be found at this link: Tag legend.

Fonts and Colors

Fonts

**Bold**	Focus molecules. Gene/Protein/ Chemical identifiers that made the user-defined cutoff and map to the Global Molecular Network are displayed with bold text.
Italics	Override molecule - Gene/ Protein/ Chemical identifier designated as Override in the dataset file.
*	Duplicates -Gene/ Protein/ Chemical identifiers marked with an asterisk indicate that multiple identifiers in the dataset file map to a single gene/ chemical in the Global Molecular Network. Please see how to resolve duplicate identifiers in Advanced Settings for more information.
A	Gene/ Protein/ Chemical ID marked as Absent. The gene/ protein/ chemical will not be used as a focus molecule or appear in networks unless you also explicitly override this flag with the override column.

+	Indicates there are other networks from the analysis that contain this gene. Right click on the + sign or on the corresponding molecule to view the related networks.
Δ	Molecules marked with the Δ (delta) have undergone a change from a previous content release. Changes include: (1) the merging of two or more molecules into one (2) the split of one molecules into two or more molecules (3) the deletion of an obsolete molecule name.
†	The † (dagger) symbol indicates custom molecules.
§	Indicates that the molecule was imported from an SMBL pathway and is not mapped in IPA.

Molecule fill colors in networks and pathways

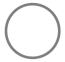
Molecules from the knowledge base – not part of the dataset.

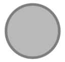
Dataset molecules that did not pass the analysis cutoffs.*

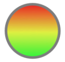
Analysis-ready molecules (i.e. passed analysis cutoffs and filters used in the analysis). The molecule colors can be set in application preferences.

*For datasets that contain only identifiers (i.e., no expression or phosphorylation values), the gray fill color identifies the focus (Analysis-Ready) molecules from that dataset.

Molecule outline colors and decorations on networks and pathways

- Default outline color.
- Selected (click outside of node to de-select).
- Highlighted (right click and choose “reset highlight” to remove highlight).
- Number of isoforms (splice variant transcripts) in overlaid expression dataset. (Hide these badges in the Overlay > Analyses, Datasets & Lists menu)
- Number of phospho sites in overlaid phosphoproteomics dataset.

Molecule color intensity: The intensity of red and green molecule colors indicates the degree of up-or-down-regulation, respectively, in an Expression dataset, and increase or decrease in phosphorylation in a Phosphorylation dataset. If normalized ratio, fold change, or log ratio/log fold change is chosen as the Expression or Phosphorylation Value type, a greater intensity of green represents a higher degree of down-regulation or decrease in phosphorylation, and a greater intensity of red represents a higher degree of up-regulation or increase in phosphorylation. In contrast, for the value types intensity/RPKM/FPKM/ Expr Other, p-value, and False Discovery Rate/ q-value, all Analysis-Ready Molecules are red by default; and a higher color intensity represents a lower (and thus more significant) value.

Molecule coloring can be customized in Application Preferences. For more information on molecule coloring, click here.

Tool Icons

Networks and pathways toolbar

Button	Name and Function
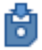	**Save:** Saves the current network diagram.
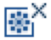	**Delete Selected:** Removes selected (highlighted) molecules from the network diagram.
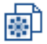	**Copy:** Saves highlighted molecules to the clipboard.
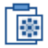	**Paste:** Returns molecules in the clipboard back to a network diagram.
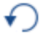	**Undo:** Reverses the last action.
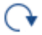	**Redo:** Repeats the last undone action.
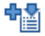	**Add to List**: Selected molecules can be added from a network, pathway, or neighborhood by clicking this icon.
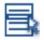	**Open Right Click Menu:** *This icon is only available on Mac computers.* It functions as a tool to open the menu that is available in Pathways, Networks, and Neighborhoods by right-clicking the mouse.
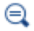	**Find Genes**: Locates a gene within a network, neighborhood, or My Pathway. Once identified, the gene will be identified in blue.
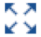	**Full Screen View:** Fill the entire monitor with the current pathway. Hit the Esc key to return to normal view.
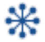	**Layout Options:** Choose from several different layouts, including radial, organic, circular, hierarchical, and subcellular.
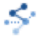	**PathTracer:** Highlight relationships and nodes of interest within networks and pathways by fading more distant nodes. Can be used to fade edges as well.
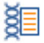	**View annotations:** Gives details of all the selected molecules.
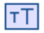	**Font sizes:** Change the font sizes of pathway objects.
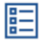	**View/ Edit Preferences:** Click to change the font sizes in network explorer. Changing the font size does not change the corresponding molecule size.
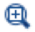	**Magnifying Lens:** Use this loupe tool to enlarge portions of pathways and networks. Click the button, then hover with the mouse over the area you wish to see in more detail.

	**Zoom Selected:** Zooms in on selected area of window.
	**Refresh:** Updates existing connections with the latest content from the Ingenuity Knowledge Base.
	**Edit Notes:** Allows you to change the notes on a saved Pathway or List.
	**Open Report:** Opens a Pathway or List report.
Build	**Build:** Opens a menu at the left of the screen for accessing Grow, Path Explorer, Connect, Trim, and Add Molecules/ Relationships.
Overlay	**Overlay:** Opens a menu at the left of the screen for overlaying Expression values, Functions and Diseases, Lists, Drugs, Canonical Pathways, and Custom Pathways and for Highlight and Species/ Tissue Highlight.
Path Designer	**Path Designer:** Transform your networks and pathways in IPA into publication quality pathway graphics rich with color, customized text and fonts, biological icons, organelles, and custom backdrops.
Pattern Search	**Pattern Search:** Scan for similarity across 100,000 analyses from QIAGEN OmicSoft by querying with any collection of genes in a network or pathway. Requires an IPA Analysis Match License.
	**Export Image:** Export an image of your network up to 600 dpi.
	**Export Data**: Export a list of genes with associated identifiers, expression values and gene details.
	**Print:** Generates a hard copy of the network diagram.
	**Email:** Send interactive pathway to a colleague.
	**Video:** Click to watch a short video tutorial about how to interpret results and use a feature.
	**More Info:** Click to navigate to an article in our online help portal that goes into detail about a feature.

Graph Navigation Tools

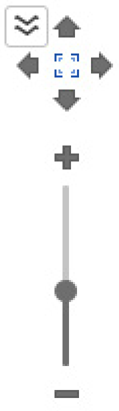

This tool is available from networks and pathways. The arrow keys allow you to move the network left, right, up, and down. The "+" button acts as a Zoom In tool. The "-" button is a Zoom Out tool. Clicking the circle in the center fits the network to the screen.

Clicking the double arrow on the top left side of the screen opens an overview of the network so that you can determine where on the page you are currently focusing.

Path Designer Tools

Button	Name and Function
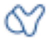	**Molecules:** Opens the Molecules menu for selecting shape style.
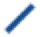	**Lines:** Draw free lines on your Path Designer pathway.
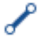	**Relationships (Edges):** Allows you to connect two molecules.
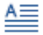	**Text:** Allows you to type free text on your Path Designer pathway.
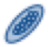	**Cell Art**: Opens the Cell Art menu for selecting organelles and other cellular structures.
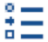	**Legend:** Links to the edit Legend menu that allows you to place a customized legend on your Path Designer pathway.
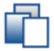	**Background:** Links to the edit Background menu that allows you to choose or upload a custom background for your Path Designer pathway.
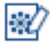	**Edit:** Opens a panel on the right of the screen for accessing the details of the Path Designer tools.
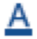	**Text Color**: Changes the color of selected text.
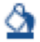	**Fill:** Allows you to choose and modify the color of the background, text background, or molecule.
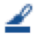	**Line Color:** Allows you to change the color of a free line or relationship.
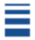	**Line Thickness:** Allows you to change the weight of a free line or relationship.
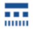	**Line Style:** Allows you to change the style of a free line or relationship.

Titel	IPA Legend
URL-Name	Legend
